# Water Arrangements upon Interaction with a Rigid Solute: Multiconfigurational Fenchone-(H_2_O)_4–7_ Hydrates

**DOI:** 10.1021/jacs.4c01891

**Published:** 2024-04-08

**Authors:** Ecaterina Burevschi, Mhamad Chrayteh, S. Indira Murugachandran, Donatella Loru, Pascal Dréan, M. Eugenia Sanz

**Affiliations:** † Department of Chemistry, King’s College London, London SE1 1DB, U.K.; ‡ PhLAM—Physique des Lasers, Atomes et Molécules, University of Lille, CNRS, UMR 8523, F-59000 Lille, France

## Abstract

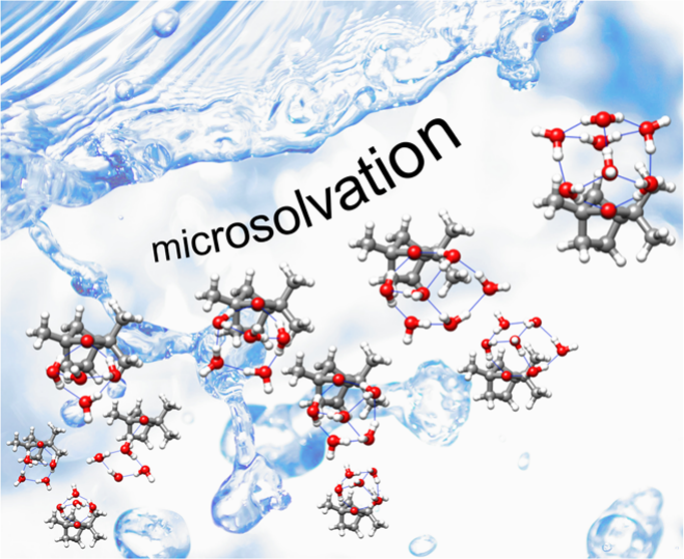

Insight into the arrangements of water molecules around solutes is important to understand how solvation proceeds and to build reliable models to describe water–solute interactions. We report the stepwise solvation of fenchone, a biogenic ketone, with 4–7 water molecules. Multiple hydrates were observed using broadband rotational spectroscopy, and the configurations of four fenchone-(H_2_O)_4_, three fenchone-(H_2_O)_5_, two fenchone-(H_2_O)_6_, and one fenchone-(H_2_O)_7_ complexes were characterized from the analysis of their rotational spectra in combination with quantum-chemical calculations. Interactions with fenchone deeply perturb water configurations compared with the pure water tetramer and pentamer. In two fenchone-(H_2_O)_4_ complexes, the water tetramer adopts completely new arrangements, and in fenchone-(H_2_O)_5_, the water pentamer is no longer close to being planar. The water hexamer interacts with fenchone as the least abundant book isomer, while the water heptamer adopts a distorted prism structure, which forms a water cube when including the fenchone oxygen in the hydrogen bonding network. Differences in hydrogen bonding networks compared with those of pure water clusters show the influence of fenchone’s topology. Specifically, all observed hydrates except one show two water molecules binding to fenchone through each oxygen lone pair. The observation of several water arrangements for fenchone-(H_2_O)_4–7_ complexes highlights water adaptability and provides insight into the solvation process.

## Introduction

Water interactions are tremendously important for life on Earth. Water is the medium where biological activity takes place and is the solvent of choice in many reactions. The unique physicochemical properties of water are related to its capacity to form extensive hydrogen bonding networks.^1,2^ Water can act as a hydrogen bond donor and as an acceptor (through the lone electron pairs of its oxygen atom), and thus, each water molecule can establish up to four hydrogen bonds. Interactions with a solute disrupt water–water hydrogen bonding, introducing competition with water–solute interactions. The latter can involve hydrogen bonding and dispersion. Depending on the balance of forces, water molecules will modify their arrangements and bind differently to various solutes. Understanding how this process happens is one of the great challenges in attaining a microscopic description of solvation at the molecular level. In this context, the study of complexes of organic molecules with increasing numbers of water molecules is essential to identify the water configurations around a solute, the noncovalent interactions involved, and to map the transition to the bulk.

Studies on clusters of microsolvated molecules, where water is added to the solute in a stepwise manner, are usually carried out in supersonic jets, where a significant portion of the complexes formed at the onset of the supersonic expansion survives and can be investigated spectroscopically.^3^ Several spectroscopic techniques across the electromagnetic spectrum have been applied to these investigations including microwave rotational, terahertz vibration–rotation, infrared ion dip, and ultraviolet hole burning, among others.^4−10^ Because of its high sensitivity to the spatial mass distribution, rotational spectroscopy has the unique advantage of being able to distinguish between close configurations (conformers and isomers), which makes it an extremely powerful technique for structural analysis.^11−13^ The development of chirped pulse Fourier transform microwave (CP-FTMW) spectroscopy,^14^ with the capacity of recording large sections of the spectrum at once, has further advanced the study of microsolvated complexes, revealing a wealth of species produced in supersonic jets. Recent reports include complexes with a large number of isomers, such as limonene-(H_2_O)_1,2_,^15,16^ aggregates of various sizes between difluoromethane and water,^17^ and complexes with a large number of water molecules, such as β-propiolactone-(H_2_O)_1–5_,^5^ 3-methylcatechol-(H_2_O)_1–5_,^18^ 3-methyl-3-oxetanemethanol-(H_2_O)_1–6_,^19^ glycolaldehyde-(H_2_O)_1–6_,^20^ benzaldehyde-(H_2_O)_1–6_,^21^ and ethanolamine-(H_2_O)_1–7_.^22^ Usually more than one isomer is observed for complexes with up to three waters. For higher-order hydrates, only one isomer was observed, except for β-propiolactone-(H_2_O)_4_,^5^ for which two isomers were found.

Here we report multiple configurations of the complexes of fenchone with four to seven water molecules, which have been identified in a supersonic jet using broadband rotational spectroscopy aided by quantum-chemical calculations. Fenchone is an abundant terpenoid with a pleasant smell that can be found in essential oils and in household goods.^23^ It is a ketone with a rigid bicyclic structure^24^ that it is not expected to suffer any changes due to its microsolvation, and it has several methyl substituents that can provide additional locations for water binding besides the carbonyl group. Fenchone is thus a good model system for the interactions of water with ketone functional groups, for which no complexes with more than three water molecules have been reported, to the best of our knowledge.

We had previously identified the hydrates of fenchone from one to three water molecules,^25^ where for all of the various isomers observed water forms chains. The first water molecule binds to one of the lone electron pairs of the carbonyl oxygen of fenchone via an O–H···O hydrogen bond, and successive water molecules bind to one another through O–H···O bonds. Further stabilization is achieved from secondary C–H···O hydrogen bonds between the last water molecule in the chain and the hydrogens of the alkyl groups of fenchone. As the number of water molecules increases, it is expected that their behavior evolves from establishing two-dimensional hydrogen bond networks, where each water molecule behaves as a hydrogen bond donor and acceptor, to three-dimensional networks characterized by having one or more water molecules acting as double donors or double acceptors. In pure water clusters, this has been observed to happen for the water hexamer,^26,27^ where water appears as three different isomers, namely, cage, book, and prism, with the cage isomer being the lowest in energy. The presence of a solute may alter this behavior.^19,20^

Our results for fenchone-(H_2_O)_4–7_ show that water adapts to fenchone to maximize favorable interactions, modifying water configurations from those preferred for pure water clusters and rearranging hydrogen bonding networks. Surprisingly, all but one of the observed complexes show two water molecules binding to the oxygen of fenchone through each of its lone electron pairs. Fenchone strongly perturbs configurations with four and five waters, giving rise to the appearance of exotic topologies and new three-dimensional (3D) hydrogen bonding networks. Interactions with six water molecules result in water adopting the configuration of the book water hexamer, the least abundant of the three observed isomers of water hexamer.^26^ In fenchone-(H_2_O)_7_ the seven waters arrange as a prism to include fenchone’s oxygen into the 3D network and overall resemble a cube similar to the global minimum of the pure water octamer.^28^

## Results and Discussion

The broadband rotational spectrum of fenchone hydrates was recorded using a CP-FTMW spectrometer in the 2–8 GHz frequency range^24,29^ (see experimental details in the Supporting Information). After removing the transitions from fenchone,^24^ pure water complexes (dimer,^30^ hexamers,^26,27^ heptamers,^31^ and nonamers^32^), as well as those belonging to fenchone-(H_2_O)_1–3_,^25^ there were still many unidentified lines in the rotational spectrum, which we suspected to arise from complexes with a higher number of water molecules. To aid spectral searches, we first explored the potential energy surface of complexes of fenchone with four to seven water molecules using the program CREST,^33^ and optimized the resulting structures at the B3LYP-D3BJ/6-311++G(d,p) level of theory using Gaussian09.^34^ Those within 6–7 kJ mol^–1^ were confirmed to be local minima by checking the sign of harmonic vibrational frequency calculations. They were further optimized at the MP2/6-311++G(d,p) and B3LYP-D3BJ/def2-TZVP levels of theory. Their rotational constants, dipole moment components, and relative energies are collected in the Supporting Information. Complexes have been labeled indicating first the number of water molecules, namely, 4w, 5w, etc., followed by a number of their position in the energy ordering (considering zero-point corrected energies) at the B3LYP-D3BJ/6-311++G(d,p) level of theory.

A large variety of low-energy isomers and topologies emerges for fenchone-(H_2_O)_4–7_, differently from the limited number of configurations predicted for fenchone-(H_2_O)_1–3_ complexes. For example, for fenchone-(H_2_O)_4_, 20 isomers are predicted within 6 kJ mol^–1^ showing 11 different topologies or arrangements of the water molecules around fenchone without considering the orientations of the hydrogen atoms (Figure S1). We can distinguish two main isomer classes depending on whether one or two water molecules bind to the carbonyl oxygen of fenchone, establishing one or two O–H···O hydrogen bonds, respectively. In fenchone-(H_2_O)_5–7_, all low-energy isomers show two water molecules interacting with fenchone through two O–H···O hydrogen bonds (Figures S4, S6, and S8). Several fenchone-water isomers are very similar to one another, showing only small variations in their theoretical rotational constants that originated by changes in the position of the hydrogen atoms of water. In some cases, this causes significant variations in the dipole moment components, which aids isomer discrimination.

**Figure 1:**
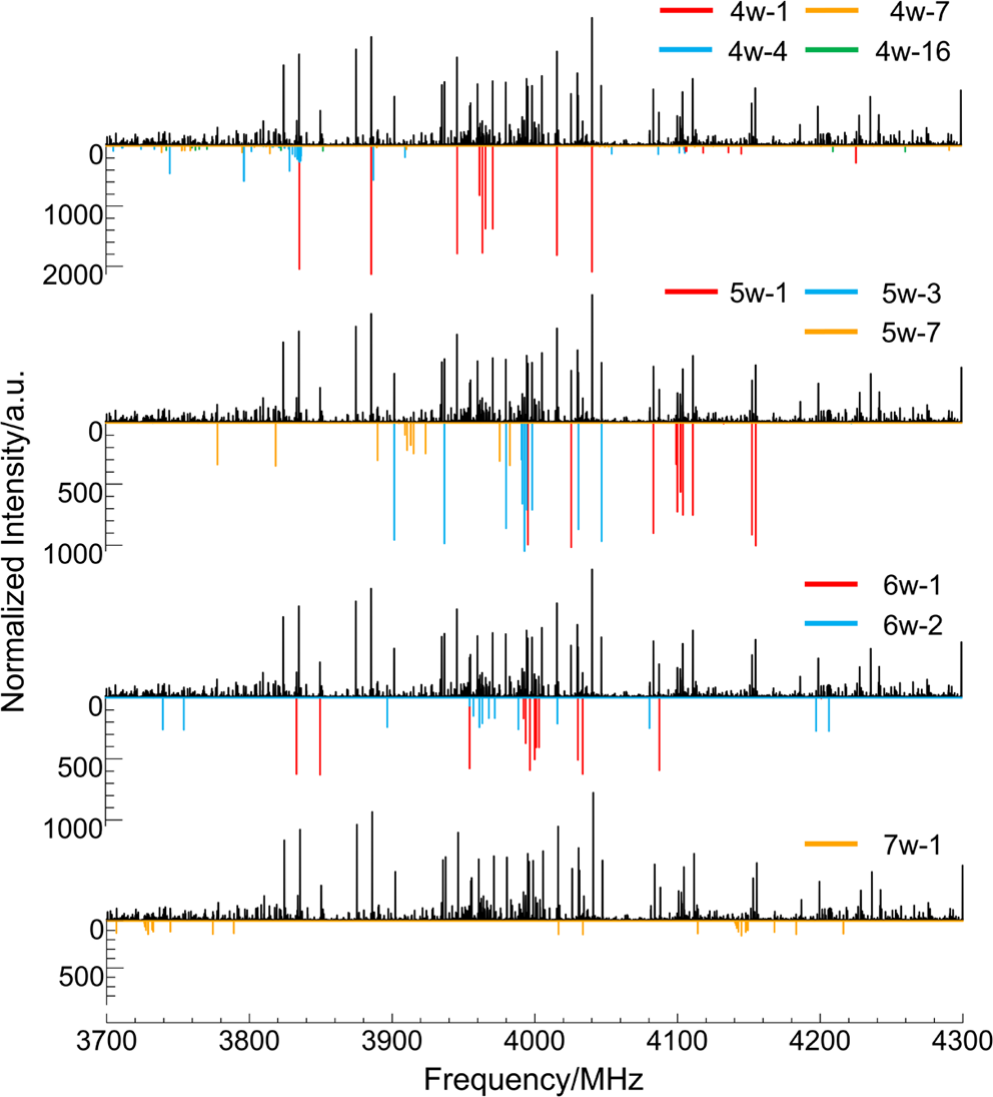
Section of the experimental spectrum of fenchone-water between 3700 and 4300 MHz. Lines arising from fenchone,^24^ fenchone-(H_2_O)_1–3_,^25^ and water clusters (H_2_O)_2,6,7,9_^26,27,30−32^ have been removed. Upper traces in black show the experimental spectrum, and lower traces show the simulated spectrum using the experimentally determined rotational constants, the MP2 theoretical dipole moment components, and experimental abundances.

**Figure 2:**
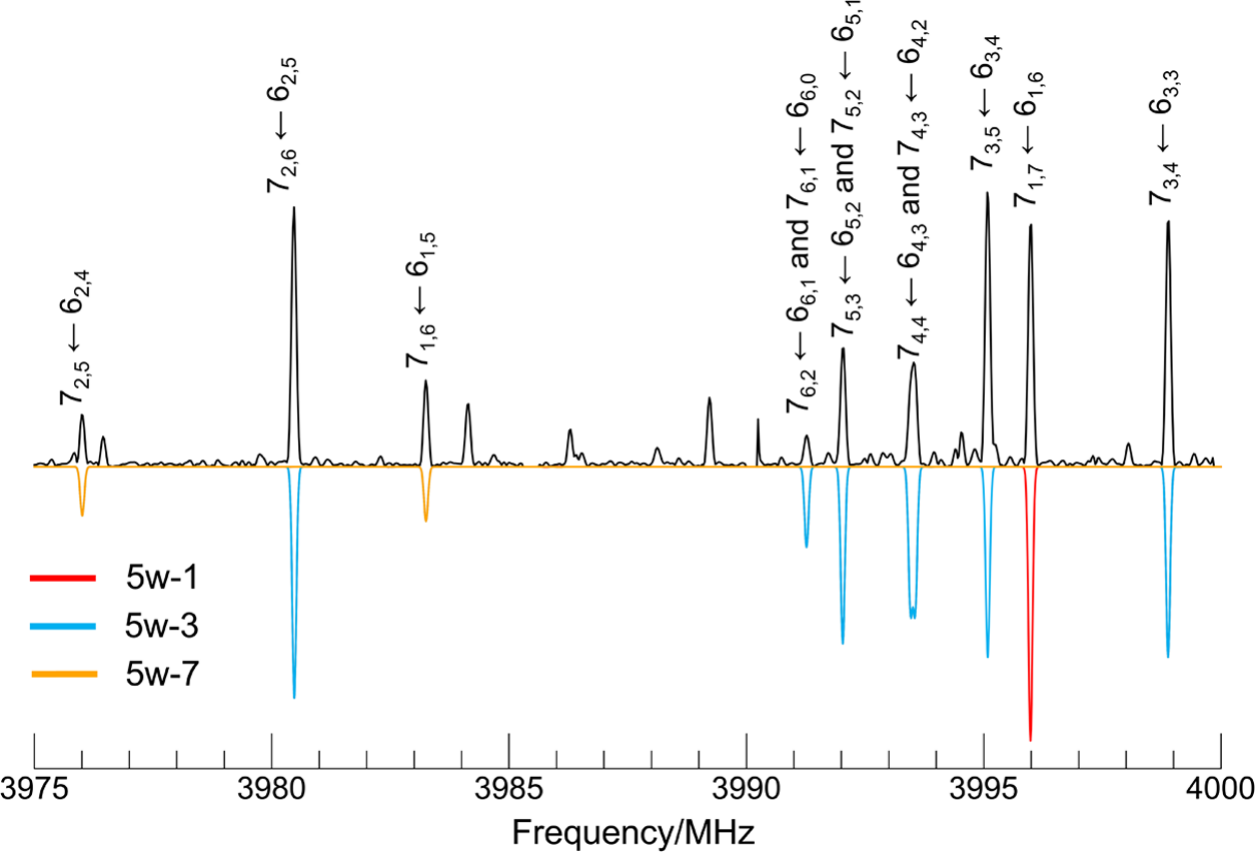
Section of the broadband rotational spectrum of fenchone-water showing rotational transitions from fenchone-(H_2_O)_5_. Upper traces in black show the experimental spectrum, and lower traces show the simulated spectrum using the experimentally determined rotational constants, the MP2 theoretical dipole moment components, and experimental abundances.

With the support of the predicted spectroscopic parameters and using the program PGOPHER,^35,36^ including its automated assignment tool based on AUTOFIT,^37^ we found four isomers of fenchone-(H_2_O)_4_, three of fenchone-(H_2_O)_5_, two of fenchone-(H_2_O)_6_, and one of fenchone-(H_2_O)_7_. Sections of the broadband rotational spectrum showing transitions from these complexes are presented in Figures 1 and 2.

Identification of the isomers was achieved by comparing theoretical and experimental rotational constants as well as comparing the predicted dipole moment components with the observed type and intensity of rotational transitions (Tables 1 and 2, and S1–S3, S6–S14). The experimental rotational constants of each of the isomers are sufficiently different for their assignment to a specific topology (see, for example, the constants for the isomers with four and five water molecules in Table 1). In the cases where one observed species could match more than one theoretical fenchone-water isomer due to their close rotational constants and similar dipole moment components, we have considered the predicted energy ordering and assigned the observed species to the lowest-energy predicted isomer of the same class (see the details in the Supporting Information).

**Table 1:** Experimental Spectroscopic Parameters of the Observed Isomers of Fenchone-(H_2_O)_4,5_

parameters^a^	**4w-1**	**4w-4**	**4w-7**	**4w-16**	**5w-1**	**5w-3**	**5w-7**
*A* (MHz)	739.1539(67)^b^	690.95083(54)	645.53165(71)	792.470(14)	577.5314(48)	616.5244(77)	662.6372(46)
*B* (MHz)	347.23424(28)	349.66403(32)	391.52937(26)	333.09855(34)	304.44236(18)	295.53093(25)	294.14773(19)
*C* (MHz)	312.16194(27)	334.39246(45)	357.69156(26)	292.56087(30)	280.63227(16)	274.245269(24)	263.67254(17)
Δ_J_ (Hz)	25.71(41)	60.5(23)	35.1(15)	20.3(11)	17.05(23)	21.57(28)	18.15(23)
Δ_JK_ (Hz)	16.9(16)		115(13)	60(23)	66.6(13)	8.3(16)	
Δ_K_ (Hz)		72(16)					
δ_J_ (Hz)	2.46(60)	8.8(19)			1.81(28)	2.37(40)	1.87(30)
*a*/*b*/*c*	y/n/n	y/n/y	y/n/y	y/n/n	y/n/n	y/n/n	y/n/n
σ (kHz)	4.8	7.9	7.5	8.6	3.8	5.4	4.0
N	118	61	55	42	124	138	96

a Spectroscopic parameters were determined using Watson’s A-reduced Hamiltonian in the *I*^r^ representation. Rotational constants *A*, *B*, and *C*; quartic centrifugal distortion constants Δ_J_, Δ_JK_, Δ_K_, and δ_J_; type of spectrum observed (*a*-, *b*-, and *c*-type) with *y* being observed and *n* not observed; σ is the rms deviation of the fit; and N is the number of fitted transitions.

b Standard error in parentheses in units of the last digit.

**Table 2:** Experimental Spectroscopic Parameters of the Observed Isomers of Fenchone-(H_2_O)_6,7_^b^

parameters^a^	**6w-1**	**6w-2**	**7w-1**
*A* (MHz)	498.0622(27)	522.0874(32)	493.6455(88)
*B* (MHz)	263.50251(18)	265.31885(24)	216.17808(30)
*C* (MHz)	234.63357(16)	227.56417(21)	197.50864(27)
Δ_J_ (Hz)	16.22(20)	16.20(32)	9.14(28)
Δ_JK_ (Hz)	18.6(13)		–16.6(21)
δ_J_ (Hz)	2.84(25)	3.39(38)	0.93(34)
*a*/*b*/*c*	y/n/n	y/n/n	y/n/n
σ (kHz)	4.4	5.3	7.5
*N*	128	84	121

a Spectroscopic parameters were determined using Watson’s A-reduced Hamiltonian in the *I*^r^ representation. Rotational constants *A*, *B*, and *C*; quartic centrifugal distortion constants Δ_J_, Δ_JK_, and δ_J_; type of spectrum observed (*a*-, *b*-, and *c*-type) with *y* being observed and *n* not observed; σ is the rms deviation of the fit; and N is the number of fitted transitions.

b Standard error in parentheses in units of the last digit.

**Figure 3:**
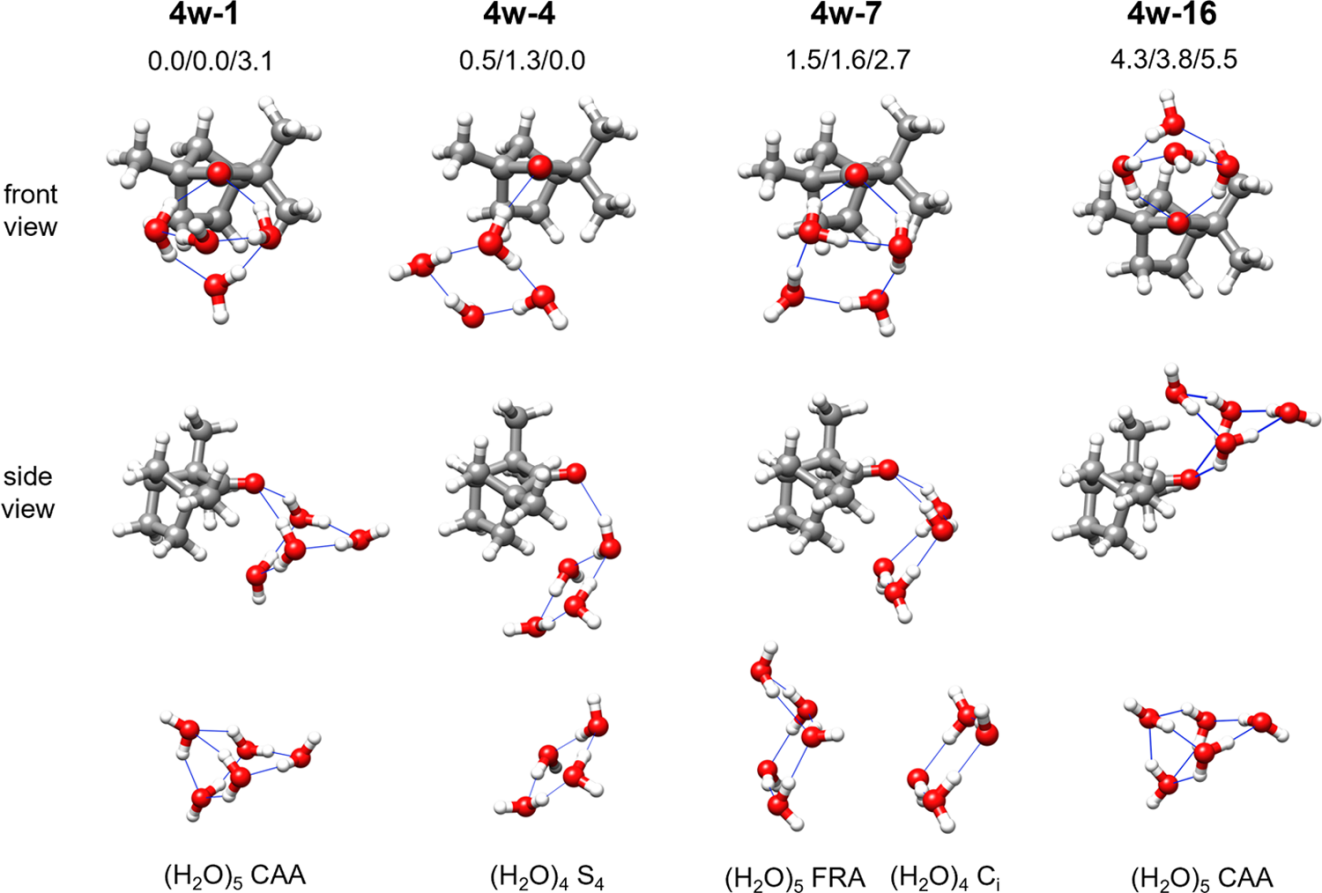
Observed isomers of fenchone-(H_2_O)_4_, their relative energies (B3-6311/B3-def2/MP2) and their comparison with pure water clusters. The labels of pure water clusters (bottom of the figure) follow those of the literature: tetramers of *S*_4_(38) and *C*_*i*_(40) symmetry, pentamer CAA^44^ (cage-A, in two different views), and pentamer FRA^44^ (fused-ring A).

The four observed isomers of fenchone-(H_2_O)_4_, **4w-1**, **4w-4**, **4w-7**, and **4w-16** (Figure 3), display three different topologies. **4w-4** is the only isomer where only one water molecule binds to the fenchone oxygen. It has the four water molecules forming a near-planar ring, with sequential O–H···O hydrogen bonds between them in a clockwise configuration and the hydrogens not participating in hydrogen bonding in an up–down–up–down (udud) arrangement like that shown by the lowest-energy water tetramer of *S*_4_ symmetry.^38^ All of the other observed isomers display two water molecules binding to fenchone, where the carbonyl oxygen acts as a double hydrogen acceptor, thus showing an antidromic hydrogen bonding arrangement.^2,39^ In these structures, the four water molecules also close a ring where sequential cooperative hydrogen bonds (homodromic cycles) are established. However, the configuration of the ring varies. In **4w-7**, the four water molecules form a near-planar ring comprising all oxygen atoms and an up–up–down–down (uudd) arrangement of the hydrogens not involved in hydrogen bonding, analogous to the pure water tetramer of *C_i_* symmetry that is second in the energy ordering.^40^ In **4w-1** and **4w-16**, the water tetramer forms a puckered ring with the nonbonding hydrogens adopting a uuud configuration. For **4w-1**, we were able to observe all ^18^O singly substituted isotopologues of the water molecules, thus making it possible to determine the oxygens’ coordinates using Kraitchman’s equations^41^ and calculate the ring puckering angle τ(OOOO) = 127(2)°. This is an entirely new arrangement of water molecules, not predicted nor previously observed for the structures of the pure water tetramer.^42^

**Figure 4:**
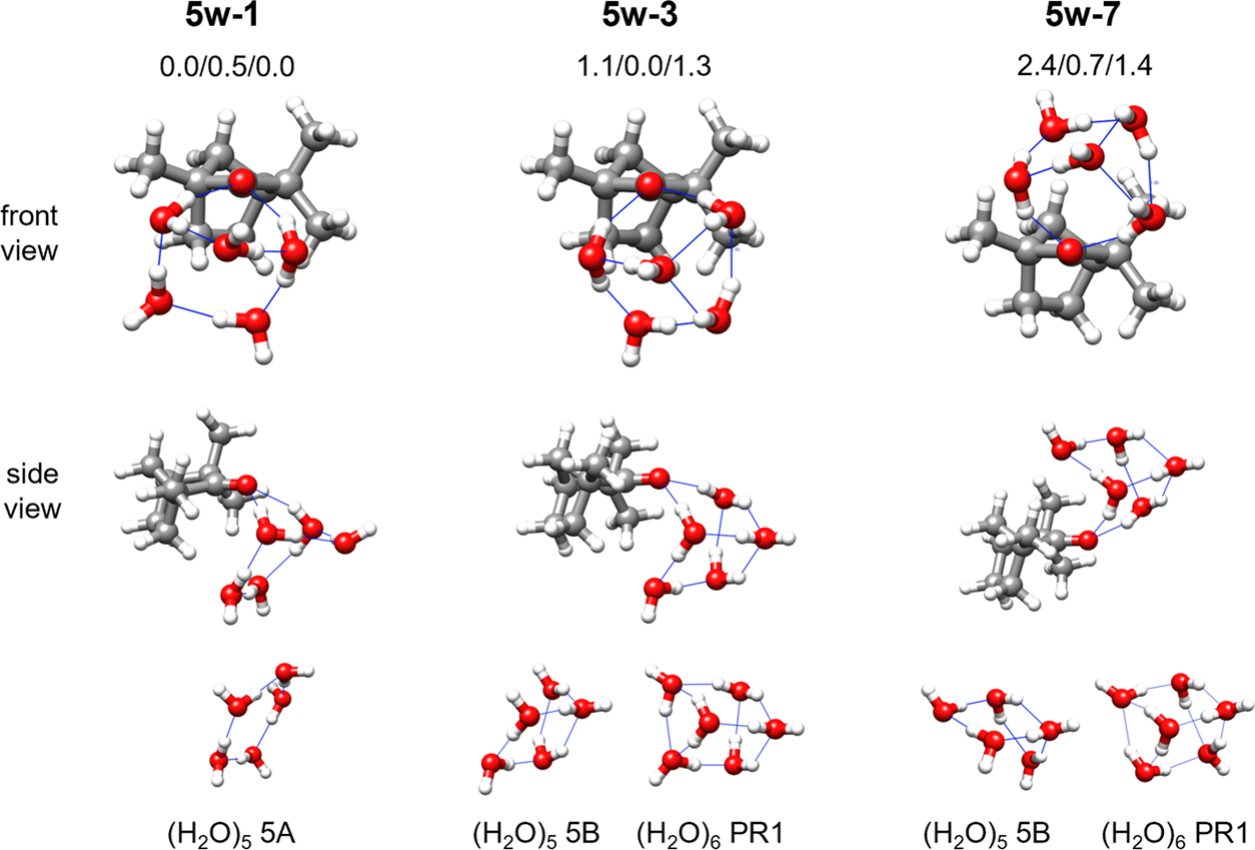
Observed isomers of fenchone-(H_2_O)_5_, their relative energies (B3-6311/B3-def2/MP2), and their comparison with pure water clusters. The labels of pure water clusters (bottom) follow those of the literature: pentamers 5A^8^ and 5B^8^ (the latter in two different views), and the lowest-energy prism hexamer PR1.^26^

All observed fenchone-(H_2_O)_5_ complexes show two water molecules binding to the carbonyl oxygen, each establishing an O–H···O hydrogen bond (Figure 4), and two different topologies. The five water molecules form a distorted puckered pentamer, where the oxygens of four waters form a near-planar ring, and that of the fifth water is at a puckering angle of about 120° (**5w-1**) or 95° (**5w-3** and **5w-7**). Accordingly, the configuration of the five waters in **5w-1** could be considered as a very distorted cyclic water pentamer, puckered from its lowest-energy configuration.^8,43^ The five waters’ arrangement in **5w-3** and **5w-7** is significantly different, as they both show, in addition to a sharper puckering angle of the fifth oxygen atom, a further hydrogen bond that closes one three- and one four-membered cycle. Their topologies are similar to that of the noncyclic pentamer 5B, recently observed in the gas phase.^8^ They differ from 5B in that the water molecule off the plane of the tetramer is rotated to interact with the carbonyl oxygen, and its angle with the plane of the tetramer is reduced. In **5w-7**, the arrangement is the specular image of the water pentamer 5B.

**Figure 5:**
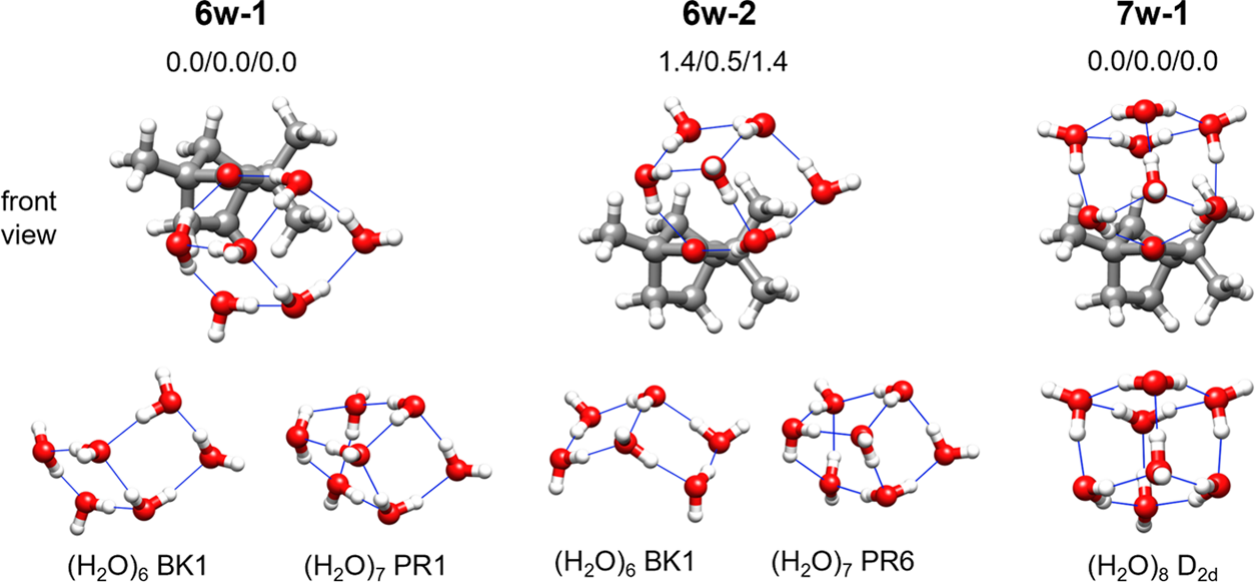
Observed isomers of fenchone-(H_2_O)_6_ and fenchone-(H_2_O)_7_, their relative energies (B3-6311/B3-def2/MP2), and their comparison with pure water clusters. The labels of pure water clusters (bottom) follow those of the literature: the lowest-energy book hexamer BK1,^26^ the prism heptamers PR1 and PR6,^31^ and the cube octamer of *D*_2*d*_ symmetry.^28^

In both observed fenchone-(H_2_O)_6_ clusters, the six water molecules display a configuration similar to the book isomer of the pure water hexamer^26^ (Figure 5). Their topologies are the same. They differ only in the relative position of the water hexamer with respect to fenchone. As in fenchone-(H_2_O)_5_, two water molecules bind to the carbonyl oxygen through O–H···O hydrogen bonds. In both isomers, the majority of the water molecules are closer to the α-carbon of fenchone, which has two methyl groups as substituents.

In fenchone-(H_2_O)_7_, the seven water molecules form a distorted water heptamer, with a prism-like structure, and also two water molecules interact with the oxygen of fenchone via O–H···O hydrogen bonds (Figure 5).

Relative abundances of the observed isomers were estimated by considering common *a*-type transitions. Line intensity in our experiment is proportional to the number density of each isomer and to the square of the corresponding dipole moment component; that is, in this case, *I*_i_ ∝ *N*_i_μ_a_^2^. We thus estimated the relative abundances as **4w-1**/**4w-4**/**4w-7**/**4w-16** = 16.1:8.9:1.4:1.0. The abundances are consistent with predictions using B3LYP-D3BJ. However, MP2 predicts **4w-4** as the global minimum and **4w-1** as the third in energy of those observed. Of the four observed isomers, **4w-1** and **4w-16** show similar topologies for the water molecules, although located differently on fenchone.

While all methods predict **4w-1** as being lower in energy than **4w-16**, the variety of topologies for fenchone-(H_2_O)_4_ may be behind the discrepancies in the prediction of energy ordering. SAPT2+/aug-cc-pVDZ calculations (see Table S15), which provide a crude approximation of the binding energies assuming that all water molecules act as one moiety and fenchone as the other, predict **4w-1** as the isomer with the highest binding energy, followed by **4w-16**, **4w-7**, and **4w-4**. Predictions closely follow the electrostatics contribution to the overall energy. **4w-4**, where a 2D rather than a 3D hydrogen bonding network is established and four O–H···O bonds rather than five, has a significantly lower electrostatic contribution than the other isomers. BSSE calculations yield complexation energies in agreement with experimental abundancies (Table S15).

Following the same procedure and also considering common *a*-type transitions, the relative abundances of isomers of fenchone-(H_2_O)_5,6_ were estimated as **5w-1**/**5w-3**/**5w-7** = 2.9:2.3:1.0 and **6w-1**/**6w-2** = 2.0:1.0. The abundances of fenchone-(H_2_O)_6_ are in agreement with relative energy predictions by the three computational methods used, and their SAPT2+ binding energies and BSSE complexation energies are also consistent with the experimental observations. Those of the complexes with five water molecules are consistent with the energy ordering predicted by B3LYP-D3BJ/6-311++G(d,p) and MP2/6-311++G(d,p). However, B3LYP-D3BJ/def2-pVTZ predicts **5w-3** lower in energy than **5w-1**. SAPT2+ calculations yield the lowest binding energy to **5w-1**, which may be related to its lower electrostatic contribution as it has one fewer hydrogen bond than **5w-3** and **5w-7**. The SAPT2+ binding energies for **5w-3** and **5w-7**, with water prism topologies comparable to those of the water molecules, are not consistent with their experimental relative abundances. **5w-7** is predicted to have a more sizable dispersion contribution and the highest binding energy. Complexation energies for **5w-3** and **5w-7** from BSSE agree with experimental abundances, but they predict a higher complexation energy for **5w-3** rather than **5w-1**.

The zero-point corrected relative energies of the observed isomers are all very close, with maximum differences of about 3 kJ mol^–1^ for **4w-1**, **4w-3**, and **4w-7**; 2 kJ mol^–1^ for **5w-1**, **5w-3**, and **5w-7**; and 1.5 kJ mol^–1^ for **6w-1** and **6w-3**. It is thus not surprising that there are some discrepancies in the energy ordering.

All observed isomers display an O–H···O as well as C–H···O hydrogen bonds. The latter typically involve the −CH_3_ of the carbons in α to the carbonyl group of fenchone and in some cases the −CH_2_ groups. These interactions can be visualized using the noncovalent interaction (NCI) analysis,^45,46^ which is based on examining the electron density and its derivatives (see Figures S3, S5, S7, and S9). Strong O–H···O bonds appear as blue pills, while weaker C–H···O interactions appear as green isosurfaces.

Since MP2 rotational constants show ∼1.9% average differences with the experimental ones, we can consider MP2 structures as very close to the actual ones and examine any possible trends in hydrogen bonding. The average differences for B3LYP-D3BJ/6-311++G(d,p) and B3LYP-D3BJ/def2-TZVP were 2.4 and 3.0%, respectively. It should be noted that we are comparing the experimental rotational constants in the ground vibrational state (*A*_0_, *B*_0_, *C*_0_) with the theoretical equilibrium values (*A*_e_, *B*_e_, *C*_e_), due to the high computational cost of including vibrational corrections for clusters of the size of fenchone-(H_2_O)_4–7_. The O–H···O bonds of water with the carbonyl oxygen become shorter with increasing numbers of water molecules, decreasing from 2.12 Å (4w), to 2.00 Å (5w), 1.98 (6w), and 1.90 Å (7w). The O–H···O bond lengths between water molecules span a larger range, typically from 1.70 to 2.10 Å. **5w-3** and **5w-7** show the largest variations with values between 1.74–2.10 Å (**5w-3**) and 1.75–2.14 Å (**5w-7**). **7w-1** has the three shortest hydrogen bonds connecting the top and bottom of the cube, with values of 1.70, 1.72, and 1.73 Å, while the other O–H···O between waters range between 1.91 and 1.99 Å.

Considering O···O distances, we determined experimentally those between the water oxygens in the **4w-1** cluster as 2.74(9), 2.80(6), 2.83(6), and 2.93(10) Å (Figure S2). These values show very clearly the disruption induced by fenchone in the hydrogen bonding network of the water tetramer, for which empirical equal O···O distances of 2.81 Å were derived.^43^ Our values can be compared with those determined for β-propiolactone-(H_2_O)_4_,^5^ of 2.68(7), 2.75(1), 2.75(2), and 2.94(8) Å, showing similar structural changes even if water molecules have a different arrangement. The MP2 O···O distances for **4w-1** are 2.75, 2.78, 2.80, and 2.85 Å, which follow the experimental trend, thus lending confidence to the use of MP2 as a proxy for the actual structures.

All species show O–H···O hydrogen bonding networks where the oxygen of fenchone is included in place of a water oxygen. Considering fenchone’s oxygen, the overall topologies mostly reproduce those displayed by pure water clusters, but interestingly, many of them do not align with those of the global minimum of the corresponding water cluster. For instance, the lowest-energy water pentamer corresponds to a cyclic planar structure. However, none of the fenchone-(H_2_O)_4_ complexes display it. In contrast, **4w-7** looks like the predicted fused-ring water pentamer FRA^44^ with one of the water molecules flipped to establish a hydrogen bond to fenchone’s oxygen. **4w-1** and **4w-16** look like the predicted cage water pentamer CAA (cage A).^44^ Similarly, the global minimum of the water hexamer is the cage isomer,^26^ but it is not exhibited by any fenchone-(H_2_O)_5_ complexes. The water molecules in **5w-3** and **5w-7** form a prism with the carbonyl oxygen similar to the prism isomer of the pure water hexamer,^26^ while their arrangement in **5w-1** does not resemble any of the observed and predicted pure water hexamers, including the bag isomers^40^ (see Figure 3). Fenchone-(H_2_O)_6_ isomers adopt prism-like structures such as those of the water heptamer.^31^**6w-1** shows an arrangement similar to the global minimum 7-PR1 of (H_2_O)_7_, while **6w-2** is similar to 7-PR6 predicted to be 4.69 kJ mol^–1^ higher in energy. Fenchone-(H_2_O)_7_ displays a cube arrangement with a hydrogen bonding network resembling that of the lowest-energy water octamer *D*_2*d*_(28) (see Figure 4).

Interactions with fenchone disrupt the transition from 2D to 3D hydrogen bonding networks of pure water clusters. The 2D → 3D transition in pure water clusters occurs when the number of water molecules is six and above. However, we observed 2D and 3D structures coexisting in fenchone-(H_2_O)_4_. 2D configurations like those of the *S*_4_ (udud) and *C*_*i*_ (uudd) pure water tetramers^38,40^ are preserved in **4w-4** and **4w-7**, respectively, while **4w-1** and **4w-16** display new 3D networks not previously observed. In all observed fenchone-(H_2_O)_5–7_ complexes, the water molecules adopt 3D hydrogen bonding networks. Those observed for fenchone-(H_2_O)_5_ differ between isomers, with one showing an unusual network probably high in energy, as it has not been predicted so far.

The observation of several isomers for the clusters with *n* = 4–6 highlights the extraordinary plasticity of water upon binding to a solute, especially considering the variation in water topologies among isomers. To our knowledge, different isomers of complexes with such a large number of water molecules have been only reported for β-propiolactone-(H_2_O)_4_,^5^ where the configuration of the waters is the same but the direction of the hydrogen bonding varies, adopting clockwise or anticlockwise arrangements. The changes between isomers in fenchone-(H_2_O)_4,5_ are more drastic and show strong departures from the lowest-energy configurations of pure water clusters. This suggests that water molecules adapt their shape and modify their hydrogen bonding to match with the solute and maximize water–solute as well as water–water interactions. The variety of water arrangements observed for other complexes of water with organic molecules seems to support this hypothesis. For example, udud water tetramer arrangements were observed for 3-methyl-3-oxetanemethanol-(H_2_O)_4_^19^ and benzaldehyde-(H_2_O)_4_,^21^ while the uudd arrangement was reported for β-propiolactone-(H_2_O)_4_.^5^ The nonplanar water pentamer arrangement in **5w-3** has been observed in the β-propiolactone-(H_2_O)_5_ complex,^5^ but benzaldehyde-(H_2_O)_5_^21^ displayed a planar ring structure. The book water hexamer observed in fenchone-(H_2_O)_6_ is also the isomer of choice in benzene-(H_2_O)_6_^47^ and benzaldehyde-(H_2_O)_6._^21^ However, a prism-like structure is preferred for glycolaldehyde-(H_2_O)_6_^20^ and 3-methyl-3-oxetanemethanol-(H_2_O)_6_. Different configurations were observed in 3-methylcatechol-(H_2_O)_4,5_.^18^ Overall, the versatility of water and the different configurations of its clusters with fenchone can provide insight into the dynamic configurations of water in the liquid phase and help model their behavior.

## Conclusions

The microsolvation of fenchone with four to seven water molecules has been characterized by broadband rotational spectroscopy supported by quantum chemistry calculations. Four, three, and two isomers have been observed for fenchone-(H_2_O)_4_, fenchone-(H_2_O)_5_, and fenchone-(H_2_O)_6_ complexes, respectively, which underlies the tremendous adaptability of water to a solute.

Water molecules arrange on fenchone driven by the O–H···O as well as C–H···O hydrogen bonds. The network of interactions is shaped by the participation of fenchone’s oxygen, which typically acts as a double hydrogen bond acceptor leading to the formation of 3D hydrogen bonding networks. Some of these show exotic configurations observed for the first time, specifically for fenchone-(H_2_O)_4_ and fenchone-(H_2_O)_5_ clusters. Overall, hydrogen bonding networks in fenchone evolve from forming chains in fenchone-(H_2_O)_1–3_,^25^ to 2D cycles and then to 3D structures involving several cycles in fenchone-(H_2_O)_4–7_.

Our observations provide details on the configurations and interactions of a large number of water molecules with a rigid ketone for the first time and can serve as a basis to improve our understanding of the initial steps of solvation.

## Supporting Information Available

Details on the computational and experimental methods; assignment; tables of spectroscopic constants; NCI plots; SAPT and BSSE calculations; lists of measured transitions; cartesian coordinates; and additional references^48−50^ (PDF)
