# Supplementary material for: Water Arrangements upon Interaction with a Rigid Solute: Multiconfigurational Fenchone-(H2O)4–7 Hydrates
Source: J Am Chem Soc. 2024 Apr 8;146(15):10925–33. doi: 10.1021/jacs.4c01891 (PMC11027134; doi:10.1021/jacs.4c01891)
Supplement: Supplementary file 1 — ja4c01891_si_001.pdf [file ja4c01891_si_001.pdf]

The Water Arrangements upon Interaction with a Rigid Solute: Multiconfigurational Fenchone-$(H_2O)_{4-7}$ Hydrates

Ecaterina Burevschi,[a] Mhamad Chrayteh,[b],† S. Indira Murugachandran,[a] Donatella Loru,[a],‡ Pascal Dréan,[b] M. Eugenia Sanz*[a]

[a] Dr. E. Burevschi, Dr. I. Murugachandran, Dr. D. Loru, Dr. M. E. Sanz
Department of Chemistry
King's College London
London, SE1 1DB, United Kingdom
E-mail: maria.sanz@kcl.ac.uk

[b] Dr. M. Chrayteh, Dr. P. Dréan
PhLAM - Physique des Lasers, Atomes et Molécules
University of Lille
CNRS, UMR 8523, F-59000 Lille, France

† Present address: CEA, French Commission for Atomic and Alternative Energies, DES, ISEC, DPME, SEIP, LDPS Univ Montpellier, Marcoule, F-30207 Bagnols-sur-Cèze, France.

‡ Present address: Deutsches Elektronen-Synchrotron DESY, Notkestraße 85, D-22607 Hamburg, Germany.

Supporting Information

Table of Contents

1. **Methods** 5

1.1. **Computational** 5

1.2. **Experimental** 5

2. **Assignment** 5

2.1. **Fenchone-(H_2O)$_4$** 5

2.2. **Fenchone-(H_2O)$_5$** 6

2.3. **Fenchone-(H_2O)$_6$** 6

2.4. **Fenchone-(H_2O)$_7$** 6

2.5. **References** 6

3. **Fenchone-(H_2O)$_4$** 8

Figure S1. The lower-energy isomers of fenchone-(H_2O)$_4$ within 6 kJ mol^{-1}. 8

Table S1. Predicted spectroscopic rotational parameters and relative energies for fenchone-(H_2O)$_4$ at the B3LYP-D3BJ/6-311++G(d,p) level of theory. 9

Table S2. Predicted spectroscopic rotational parameters and relative energies for fenchone-(H_2O)$_4$ at the B3LYP-D3BJ/def2-TZVP level of theory. 9

Table S3. Predicted spectroscopic rotational parameters and relative energies for fenchone-(H_2O)$_4$ at the MP2/6-311++G(d,p) level of theory. 10

Table S4. Experimental spectroscopic parameters of the observed isotopic species of isomer **4w-1** of fenchone-(H_2O)$_4$. Oxygen numbering is given in Figure S2. 12

Figure S2. Overlay of the MP2/6-311++G(d,p) structure of the **4w-1** isomer of fenchone-(H_2O)$_4$ with the experimental substitution coordinates of the water oxygens, represented as yellow spheres. The O···O distances are shown on the left figure in blue (experimental) and black (MP2), while the MP2 O–H···O hydrogen bonds are shown on the right figure in black. 12

Table S5. Experimental r_s coordinates (in Å) in the principal axis frame of the water oxygen atoms of the fenchone **4w-1** complex, along with those obtained by *ab initio* calculations. 12

Figure S3. Overlays of the MP2/6-311++G(d,p) structures of the observed isomers of fenchone-(H_2O)$_4$ with NCI plots. The NCI isosurfaces (s = 0.5) are shown, for values of sign(λ2)ρ ranging from −0.025 to +0.025 au. Blue indicates strong attractive interaction; green indicates weak attractive interaction; red indicates strong repulsive interaction. 13

4. **Fenchone-(H_2O)$_5$** 14

Figure S4. The lower-energy isomers of fenchone-(H_2O)$_5$ within 6 kJ mol^{-1}. 14

Table S6. Predicted spectroscopic rotational parameters and relative energies for fenchone-(H_2O)$_5$ at the B3LYP-D3BJ/6-311++G(d,p) level of theory. 15

Table S7. Predicted spectroscopic rotational parameters and relative energies for fenchone-(H_2O)$_5$ at the B3LYP-D3BJ/def2-TZVP level of theory. 15

Table S8. Predicted spectroscopic rotational parameters and relative energies for fenchone-(H_2O)$_5$ at the MP2/6-311++G(d,p) level of theory. 16

Figure S5. Overlays of the MP2/6-311++G(d,p) structures of the observed isomers of fenchone-(H_2O)$_5$ with NCI plots. The NCI isosurfaces (s = 0.5) are shown, for values of sign(λ2)ρ ranging from −0.025 to +0.025 au. Blue indicates strong attractive interaction; green indicates weak attractive interaction; red indicates strong repulsive interaction. 17

5. **Fenchone-(H_2O)$_6$** 18

Figure S6. The lower-energy isomers of fenchone-(H_2O)$_6$ within 7 kJ mol^{-1}. 18

Table S9. Predicted spectroscopic rotational parameters and relative energies for fenchone-(H_2O)$_6$ at the B3LYP-D3BJ/6-311++G(d,p) level of theory. 19

Table S10. Predicted spectroscopic rotational parameters and relative energies for fenchone-(H_2O)$_6$ at the B3LYP-D3BJ/def2-TZVP level of theory. 20

Table S11. Predicted spectroscopic rotational parameters and relative energies for fenchone-(H_2O)$_6$ at the MP2/6-311++G(d,p) level of theory. 21

Figure S7. Overlays of the MP2/6-311++G(d,p) structures of the observed isomers of fenchone-(H_2O)$_6$ with NCI plots. The NCI isosurfaces (s = 0.5) are shown, for values of sign(λ2)ρ ranging from −0.025 to +0.025 au. Blue indicates strong attractive interaction; green indicates weak attractive interaction; red indicates strong repulsive interaction. 22

6. Fenchone-(H_2O)$_7$ 23

Figure S8. The lower-energy isomers of fenchone-(H_2O)$_7$ within 7 kJ mol^{-1}. 23

Table S12. Predicted spectroscopic rotational parameters and relative energies for fenchone-(H_2O)$_7$ at the B3LYP-D3BJ/6-311++G(d,p) level of theory. 24

Table S13. Predicted spectroscopic rotational parameters and relative energies for fenchone-(H_2O)$_7$ at the B3LYP-D3BJ/def2-TZVP level of theory. 24

Table S14. Predicted spectroscopic rotational parameters and relative energies for fenchone-(H_2O)$_7$ at the MP2/6-311++G(d,p) level of theory. 25

Figure S9. Overlays of the MP2/6-311++G(d,p) structures of the observed isomers of fenchone(H_2O)$_7$ with NCI plots. The NCI isosurfaces (s = 0.5) are shown, for values of sign(λ2)ρ ranging from −0.025 to +0.025 au. Blue indicates strong attractive interaction; green indicates weak attractive interaction; red indicates strong repulsive interaction. 26

7. SAPT and BSSE energy calculations 27

8. Measured rotational transitions 28

8.1. Fenchone-(H_2O)$_4$ 28

Table S16. Measured frequencies (ν_{obs}) and residuals ($\nu_{obs} - \nu_{cal}$) of the rotational transitions of isomer **4w-1** of fenchone-(H_2O)$_4$. 28

Table S17. Measured frequencies (ν_{obs}) and residuals ($\nu_{obs} - \nu_{cal}$) of the rotational transitions of the $^{18}O_1$ isotopologue of isomer **4w-1** of fenchone-(H_2O)$_4$. 31

Table S18. Measured frequencies (ν_{obs}) and residuals ($\nu_{obs} - \nu_{cal}$) of the rotational transitions of the $^{18}O_2$ isotopologue of isomer **4w-1** of fenchone-(H_2O)$_4$. 32

Table S19. Measured frequencies (ν_{obs}) and residuals ($\nu_{obs} - \nu_{cal}$) of the rotational transitions of the $^{18}O_3$ isotopologue of isomer **4w-1** of fenchone-(H_2O)$_4$. 33

Table S20. Measured frequencies (ν_{obs}) and residuals ($\nu_{obs} - \nu_{cal}$) of the rotational transitions of the $^{18}O_4$ isotopologue of isomer **4w-1** of fenchone-(H_2O)$_4$. 34

Table S21. Measured frequencies (ν_{obs}) and residuals ($\nu_{obs} - \nu_{cal}$) of the rotational transitions of isomer **4w-4** of fenchone-(H_2O)$_4$. 35

Table S21 (cont.). Measured frequencies (ν_{obs}) and residuals ($\nu_{obs} - \nu_{cal}$) of the rotational transitions of isomer **4w-4** of fenchone-(H_2O)$_4$. 36

Table S22. Measured frequencies (ν_{obs}) and residuals ($\nu_{obs} - \nu_{cal}$) of the rotational transitions of isomer **4w-7** of fenchone-(H_2O)$_4$. 37

Table S23. Measured frequencies (ν_{obs}) and residuals ($\nu_{obs} - \nu_{cal}$) of the rotational transitions of isomer **4w-16** of fenchone-(H_2O)$_4$. 38

8.2. Fenchone-(H_2O)$_5$ 39

Table S24. Measured frequencies (ν_{obs}) and residuals ($\nu_{obs} - \nu_{cal}$) of the rotational transitions of isomer **5w-3** of fenchone-(H_2O)$_5$. 39

Table S24 (cont.). Measured frequencies (ν_{obs}) and residuals ($\nu_{obs} - \nu_{cal}$) of the rotational transitions of isomer **5w-3** of fenchone-(H_2O)$_5$. 40

Table S24 (cont.). Measured frequencies (ν_{obs}) and residuals ($\nu_{obs} - \nu_{cal}$) of the rotational transitions of isomer **5w-3** of fenchone-(H_2O)$_5$. 41

Table S25. Measured frequencies (ν_{obs}) and residuals ($\nu_{obs} - \nu_{cal}$) of the rotational transitions of isomer **5w-1** of fenchone-(H_2O)$_5$. 42

Table S25 (cont.). Measured frequencies (ν_{obs}) and residuals ($\nu_{obs} - \nu_{cal}$) of the rotational transitions of isomer **5w-1** of fenchone-(H_2O)$_5$. 43

Table S25 (cont.). Measured frequencies (ν_{obs}) and residuals ($\nu_{obs} - \nu_{cal}$) of the rotational transitions of isomer **5w-1** of fenchone-(H_2O)$_5$. 44

Table S26. Measured frequencies (ν_{obs}) and residuals ($\nu_{obs} - \nu_{cal}$) of the rotational transitions of isomer **5w-7** of fenchone-(H_2O)$_5$. 44

Table S26 (cont.). Measured frequencies (ν_{obs}) and residuals ($\nu_{obs} - \nu_{cal}$) of the rotational transitions of isomer **5w-7** of fenchone-(H_2O)$_5$. 45

8.3. Fenchone-(H_2O)$_6$ 46

Table S27. Measured frequencies (ν_{obs}) and residuals ($\nu_{obs} - \nu_{cal}$) of the rotational transitions of isomer **6w-1** of fenchone-(H_2O)$_6$. 46

Table S27 (cont.). Measured frequencies (ν_{obs}) and residuals ($\nu_{obs} - \nu_{cal}$) of the rotational transitions of isomer **6w-1** of fenchone-(H_2O)$_6$. 47

Table S27 (cont.). Measured frequencies (ν_{obs}) and residuals ($\nu_{obs} - \nu_{cal}$) of the rotational transitions of isomer **6w-1** of fenchone-(H_2O)$_6$. 48

Table S28. Measured frequencies (ν_{obs}) and residuals ($\nu_{obs} - \nu_{cal}$) of the rotational transitions of isomer **6w-2** of fenchone-(H_2O)$_6$. 48

Table S28 (cont.). Measured frequencies (ν_{obs}) and residuals ($\nu_{obs} - \nu_{cal}$) of the rotational transitions of isomer **6w-2** of fenchone-(H_2O)$_6$. 49

8.4. Fenchone-(H_2O)$_7$ 50

Table S29. Measured frequencies (ν_{obs}) and residuals ($\nu_{obs} - \nu_{cal}$) of the rotational transitions of isomer **7w-1** of fenchone-(H_2O)$_7$. 50

Table S29 (cont.). Measured frequencies (ν_{obs}) and residuals ($\nu_{obs} - \nu_{cal}$) of the rotational transitions of isomer **7w-1** of fenchone-(H_2O)$_7$. 51

Table S29 (cont.). Measured frequencies (ν_{obs}) and residuals ($\nu_{obs} - \nu_{cal}$) of the rotational transitions of isomer **7w-1** of fenchone-(H_2O)$_7$. 52

9. Cartesian coordinates of observed species 53

Table S30. Cartesian coordinates of isomer 4w-1 of fenchone-(H_2O)$_4$ from MP2/6-311++G(d,p) level of theory. 53

Table S31. Cartesian coordinates of isomer 4w-4 of fenchone-(H_2O)$_4$ from MP2/6-311++G(d,p) level of theory. 54

Table S32. Cartesian coordinates of isomer 4w-7 of fenchone-(H_2O)$_4$ from MP2/6-311++G(d,p) level of theory. 55

Table S33. Cartesian coordinates of isomer 4w-16 of fenchone-(H_2O)$_4$ from MP2/6-311++G(d,p) level of theory. 56

Table S34. Cartesian coordinates of isomer 5w-3 of fenchone-(H_2O)$_5$ from MP2/6-311++G(d,p) level of theory. 57

Table S35. Cartesian coordinates of isomer 5w-1 of fenchone-(H_2O)$_5$ from MP2/6-311++G(d,p) level of theory. 58

Table S36. Cartesian coordinates of isomer 5w-7 of fenchone-(H_2O)$_5$ from MP2/6-311++G(d,p) level of theory. 59

Table S37. Cartesian coordinates of isomer 6w-1 of fenchone-(H_2O)$_6$ from MP2/6-311++G(d,p) level of theory. 60

Table S38. Cartesian coordinates of isomer 6w-2 of fenchone-(H_2O)$_6$ from MP2/6-311++G(d,p) level of theory. 61

Table S39. Cartesian coordinates of isomer 7w-1 of fenchone-(H_2O)$_7$ from MP2/6-311++G(d,p) level of theory. 62

1. Methods

1.1. Computational

The potential energy surface of fenchone-$(H_2O)_{4-7}$ was explored using the GFN-xTB method in CREST[1], a program that uses semiempirical tight-binding methods in combination with metadynamics for fast sampling. The returned structures were then further optimized using B3LYP-D3BJ with the 6-311++G(d,p) and def2-TZVP basis sets, and MP2/6-311++G(d,p), within Gaussian09[2]. Harmonic vibrational calculations were performed on the lower-energy structures to confirm that they were local minima in the potential energy surface and to obtain their zero-point energies. For fenchone-$(H_2O)_7$ no zero-point corrected energies were obtained at MP2 level due to the high computational cost. The rotational constants, dipole moment components and relative energies for the lower-energy complexes are collected in tables below.

The non-covalent interaction (NCI) analysis[3] in combination with Multiwfn[4] was used to visualize the non-covalent interactions established between fenchone and the water molecules.

1.2. Experimental

The 2-8 GHz broadband rotational spectrometer at King's College London[5,6] was used to record the spectrum of fenchone-water. (*R*)-fenchone (Sigma-Aldrich, 98%) was placed in a bespoke heating receptacle attached to the nozzle valve and kept at a temperature of 373 K, which was previously found to yield optimal signals. Water was placed in an external receptacle and kept at room temperature. Both compounds were seeded in neon as carrier gas at a pressure of 5 bar, and conducted to the vacuum chamber, where they form a supersonic jet that was interrogated with microwave radiation. Our instrument operates in a cyclic manner. Each molecular pulse, of 1000 μs length, was polarized with four chirped microwave pulses of 4 μs duration, applied 100 μs after the end of the molecular pulse, and spaced 30 μs. An arbitrary waveform generator was used to generate the chirped microwave pulses, which were then amplified using a travelling wave tube amplifier with peak power of 200 W.[5,6] The free induction decay of the polarized species was collected for 20 μs, stored in a fast oscilloscope in the time domain and transformed to the frequency domain using a fast Fourier transform. Our lines have typically FWHM of about 110 kHz, and the frequency accuracy is better than 10 kHz.

2. Assignment

Measured transitions for each of the observed isomers were fit to the semirigid rotor Hamiltonian of Watson[7] in the I^r representation and the A reduction, using Pickett's programs.[8]

The experimental rotational constants of the observed isomers of fenchone-$(H_2O)_{4-7}$ are sufficiently different to one another as to be able to match them to different isomer topologies (see Tables 1-2 and S1-S3, S6-S14). Typically, there are a few isomers predicted to have the same water arrangements around fenchone that differ only in the positions of the hydrogen atoms of the water molecules (see Figs. S1, S4, S6, S8). In some cases, this results in significant changes in the dipole moment components, which helps with isomer discrimination. Where one observed species could match more than one theoretical isomer due to their close rotational constants and similar dipole moment components, we have considered the predicted energy ordering to make a tentative assignment. We describe below how we proceed to identify observed species.

2.1. Fenchone-$(H_2O)_4$

The rotational constants of the first observed isomer of fenchone-$(H_2O)_4$ (first column in Table 1) could match those of 1, 2, 3 and 5. Considering the non-observation of *c*-type transitions, isomers 3 and 5 can be ruled out. The rotational constants and dipole moment components of 1 and 2 are very much alike, because they only differ in the position of the hydrogen atoms of the water molecules. They have the same predicted dipole moment components, so it is not possible to discriminate them on this basis. Since 1 is consistently predicted to be lower in energy than 2, we assign the first observed isomer to isomer **4w-1**.

Similar arguments can be applied to the assignment of the next fenchone-$(H_2O)_4$ species. Its rotational constants (second column in Table 1) match those of isomers 4 and 6, which only differ in the clockwise (isomer 4) or anticlockwise (isomer 6) orientation of the hydrogen bonds between the water molecules. The observation of weaker *a*- and stronger *c*-type lines is also in agreement with the predicted $\mu_a < \mu_c$ for isomers 4 and 6. No *b*-type lines were observed but it is not possible to distinguish between isomers 4 and 6 due to this, as even for isomer 6 the predicted intensity for *b*-type transitions would have been within our baseline.

Isomer 4 is predicted to be lower in energy by all computational methods, and therefore we assigned this species to isomer **4w-4**.

The next observed species, third column in Table 1, could correspond to isomers 7 or 12, considering both the experimental rotational constants and the observed rotational transitions. 7 and 12 only difference is in the clockwise (7) or anticlockwise (12) direction of the hydrogen bonding. We assign this species to isomer **4w-7**, because 7 is consistently predicted to be lower in energy.

For the fourth observed fenchone-$(H_2O)_4$ species, fourth column in Table 1, possible matches are 16, 17,19 and 20. Isomer 16 is the lowest energy one at B3LYP-D3BJ level and 20 is the lowest at MP2 level. Considering that the relative energy ordering predicted by B3LYP-D3BJ is in better agreement with the estimated relative abundances (see text), we assigned this isomer to **4w-16**.

There are isomers lower in energy than **4w-16** which have not been observed. We have carefully looked for transitions that could correspond to other isomers, and especially to **4w-8**, **4w-9** and **4w-10**, but we have not been able to find any suitable patterns in the spectrum. The non-observation of several isomers can be rationalized in terms of their relaxation to lower-energy ones due to collisions with the carrier gas at the onset of the supersonic expansion. This can occur if the barriers for relaxation are lower than 400 cm^{-1} [9] for processes involving one degree of freedom, or lower than 1000 cm^{-1} [10,11] for processes involving multiple degrees of freedom. Modelling these relaxations is difficult because they involved the concerted motions of several atoms.

2.2. Fenchone-$(H_2O)_5$

The rotational constants of the isomer of fenchone-$(H_2O)_5$ in the fifth column in Table 1 could match those of 1, 2, 6, 8 or 9. Only *a*-type transitions are observed, which is consistent with the higher value of μ_a for all possible isomers, and rules out 6 and 9 as they have large values of μ_c and we should have detected *c*-type transitions if our observed species would correspond to one of them. 1 is lower in energy considering B3LYP-D3BJ calculations, 2 slightly lower than 1 by MP2 calculations, but by an almost negligible energy difference of 1.5 cm^{-1}, so we have assigned this species to **5w-1**. Likewise, the species in the sixth column of column 1 could be 3, 10 or 18. All these isomers are predicted to have similar rotational constants and dipole moment components. Since all computational methods predict 3 to be the lowest in energy, we assigned this species as **5w-3**. The third observed isomer of fenchone-$(H_2O)_5$, seventh column in Table 1, comparing its experimental rotational constants with the theoretical ones, could correspond to 7 or 17. 7 is consistently predicted to be lower in energy, so we assigned this species to **5w-7**.

2.3. Fenchone-$(H_2O)_6$

The rotational constants of the fenchone-$(H_2O)_6$ in the first column in Table 2 are only consistent with those of isomer 1, so we identify this species as **6w-1**. The second isomer, in the second column in Table 2, can match the theoretical constants and dipole moment components of isomers 2 and 3, and because isomer 2 is predicted as lower in energy by all computational methods used, we assigned this species as isomer **6w-2**.

2.4. Fenchone-$(H_2O)_7$

The only observed isomer of fenchone-$(H_2O)_7$ has rotational constants (third column in Table 2) that could correspond to isomers 1, 4, 5, 6 or 7. Given that isomer 1 is predicted to be lowest in energy by all methods, we assign this species to isomer **7w-1**.

2.5. References

[1] P. Pracht, F. Bohle, S. Grimme, *Phys. Chem. Chem. Phys.* **2020**, *22*, 7169–7192.

[2] M. J. Frisch, G. W. Trucks, H. B. Schlegel, G. E. Scuseria, M. A. Robb, J. R. Cheeseman, G. Scalmani, V. Barone, B. Mennucci, G. A. Petersson, H. Nakatsuji, M. Caricato, X. Li, H. P. Hratchian, A. F. Izmaylov, J. Bloino, G. Zheng, J. L. Sonnenberg, M. Hada, M. Ehara, K. Toyota, R. Fukuda, J. Hasegawa, M. Ishida, T. Nakajima, Y. Honda, O. Kitao, H. Nakai, T. Vreven, J. Montgomery, J. A., J. E. Peralta, F. Ogliaro, M. Bearpark, J. J. Heyd, E. Brothers, K. N. Kudin, V. N. Staroverov, R. Kobayashi, J. Normand, K. Raghavachari, A. Rendell, J. C. Burant, S. S. Iyengar, J. Tomasi, M. Cossi, N. Rega, J. M. Millam, M. Klene, J. E. Knox, J. B. Cross, V. Bakken, C. Adamo, J. Jaramillo, R. Gomperts, R. E. Stratmann, O. Yazyev, A. J. Austin, R. Cammi, C. Pomelli, J. W. Ochterski, R. L. Martin, K. Morokuma, V. G. Zakrzewski, G. A. Voth, P. Salvador, J. J. Dannenberg, S. Dapprich, A. D. Daniels, Ö. Farkas, J. B. Foresman, J. V. Ortiz, J. Cioslowski, D. J. Fox, *Gaussian 09, Revision E. 01; Gaussian*, **2009**.

[3] E. R. Johnson, S. Keinan, P. Mori-Sánchez, J. Contreras-García, A. J. Cohen, W. Yang, *J. Am.*

Chem. Soc. **2010**, *132*, 6498–6506.
[4] T. Lu, F. Chen, *J. Comput. Chem.* **2012**, *33*, 580–592.
[5] D. Loru, M. A. Bermúdez, M. E. Sanz, *J. Chem. Phys.* **2016**, *145*, 074311–8.
[6] D. Loru, I. Peña, M. E. Sanz, *J. Mol. Spectrosc.* **2017**, *335*, 93–101.
[7] J. K. G. Watson, in *Vib. Spectra Struct. Vol.6. A Ser. Adv.* (Ed.: J.R. Durig), **1977**, pp. 1–89.
[8] H. M. Pickett, *J. Mol. Spectrosc.* **1991**, *148*, 371–377.
[9] R. S. Ruoff, T. D. Klots, T. Emilsson, H. S. Gutowsky, *J. Chem. Phys.* **1990**, *93*, 3142–3150.
[10] G. M. Florio, R. A. Christie, K. D. Jordan, T. S. Zwier, *J. Am. Chem. Soc.* **2002**, *124*, 10236–10247.
[11] M. E. Sanz, A. Lesarri, M. I. Peña, V. Vaquero, V. Cortijo, J. C. López, J. L. Alonso, *J. Am. Chem. Soc.* **2006**, *128*, 3812–3817.

3. Fenchone-(H_2O)$_4$

Figure S1. The lower-energy isomers of fenchone-(H_2O)$_4$ within 6 kJ mol^{-1}.

**4w-1** 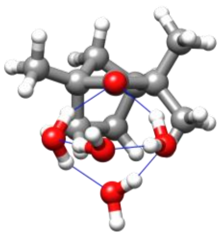	**4w-2** 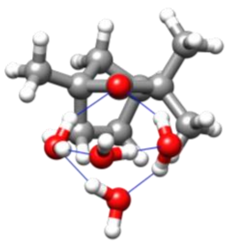	**4w-3** 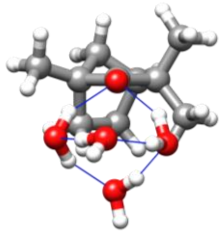	**4w-4** 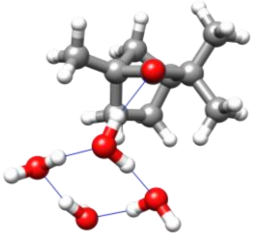
**4w-5** 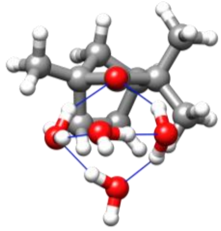	**4w-6** 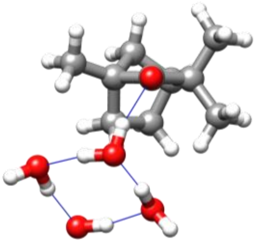	**4w-7** 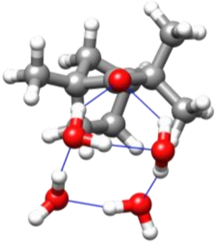	**4w-8** 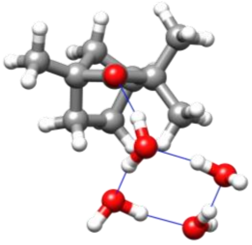
**4w-9** 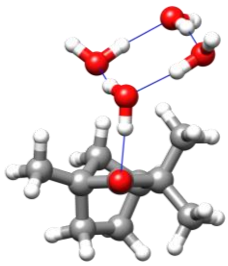	**4w-10** 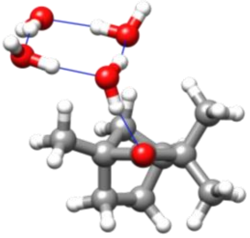	**4w-11** 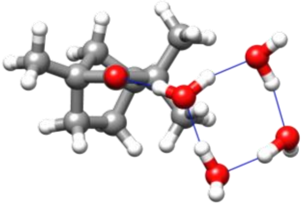	**4w-12** 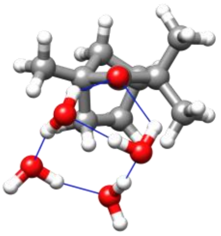
**4w-13** 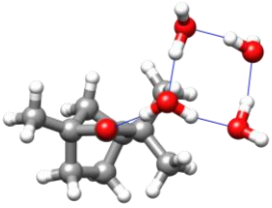	**4w-14** 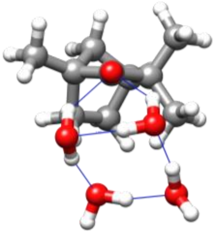	**4w-15** 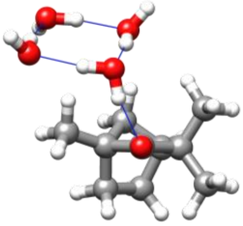	**4w-16** 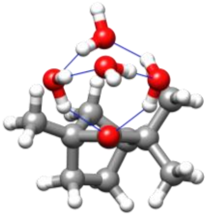
**4w-17** 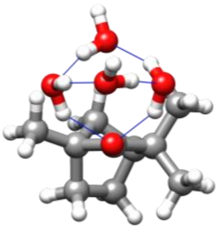	**4w-18** 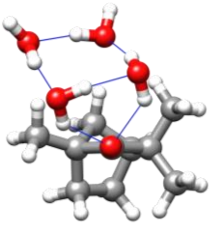	**4w-19** 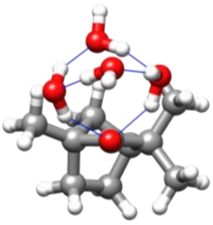	**4w-20** 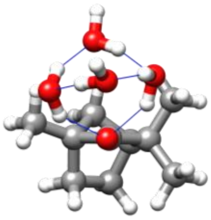

Table S1. Predicted spectroscopic rotational parameters and relative energies for fenchone-$(H_2O)_4$ at the B3LYP-D3BJ/6-311++G(d,p) level of theory.

Parameter	1	2	3	4	5	6	7	8	9	10
A^a (MHz)	747.6	750.4	743.0	699.7	745.4	696.1	649.1	712.0	738.9	776.9
B (MHz)	359.3	357.8	359.9	358.0	358.3	360.2	404.9	328.6	337.0	322.7
C (MHz)	321.7	319.9	323.0	343.9	321.1	345.0	368.4	321.9	314.2	303.9
$\|\mu_a\|^b$ (D)	3.9	3.8	3.3	1.3	3.3	1.4	3.2	1.8	1.7	1.5
$\|\mu_b\|$	0.3	0.1	0.3	0.3	0.0	0.8	1.5	0.4	0.7	1.7
$\|\mu_c\|$	0.2	0.2	1.4	3.0	1.4	3.0	2.9	2.8	2.7	2.2
ΔE^c (cm^{-1})	0.0	47.5	54	283.1	106.8	316.8	278.4	385.8	451.5	366.9
ΔE_0^d (cm^{-1})	0.0	30.3	42.4	45.9	56.0	69.1	129.3	140.5	162.4	171.8

[a] *A*, *B*, *C* are the rotational constants. [b] $|\mu_a|$, $|\mu_b|$, $|\mu_c|$ are the absolute values of the electric dipole moment components along the principal inertial axes *a*, *b* and *c*. [c] ΔE are the energies relative to the lowest energy isomer. [d] ΔE_0 are the zero-point corrected relative energies.

Table S1 (cont.). Predicted spectroscopic rotational parameters and relative energies for fenchone-$(H_2O)_4$ at the B3LYP-D3BJ/6-311++G(d,p) level of theory.

Parameter	11	12	13	14	15	16	17	18	19	20
A^a (MHz)	821.0	667.4	827.5	645.5	771.1	799.5	792.1	713.1	797.9	789.8
B (MHz)	276.4	400.1	281.2	393.4	327.5	342.2	343.2	378.1	338.0	339.3
C (MHz)	266.5	363.5	259.8	358.7	306.0	299.7	301.5	331.9	297.1	299.2
$\|\mu_a\|^b$ (D)	2.4	3.4	2.4	3.3	1.6	4.2	3.4	3.1	4.3	3.5
$\|\mu_b\|$	2.7	1.5	2.1	0.3	1.7	0.2	0.1	1.4	0.1	0.1
$\|\mu_c\|$	0.0	3.1	1.7	3.2	2.4	0.0	1.6	3.0	0.0	1.6
ΔE^c (cm^{-1})	416.8	377.4	430.7	370.1	480.5	321.1	357.4	458.8	340.9	380.5
ΔE_0^d (cm^{-1})	180.0	191.4	191.6	250.2	262.1	357.5	368.5	368.5	395.7	410.2

[a] *A*, *B*, *C* are the rotational constants. [b] $|\mu_a|$, $|\mu_b|$, $|\mu_c|$ are the absolute values of the electric dipole moment components along the principal inertial axes *a*, *b* and *c*. [c] ΔE are the energies relative to the lowest energy isomer. [d] ΔE_0 are the zero-point corrected relative energies.

Table S2. Predicted spectroscopic rotational parameters and relative energies for fenchone-$(H_2O)_4$ at the B3LYP-D3BJ/def2-TZVP level of theory.

Parameter	1	2	3	5	4	6	7	12	14	8
A^a (MHz)	749.2	751.4	746.4	748.6	702.2	700.3	654.5	666.0	666.1	717.6
B (MHz)	360.9	360.0	360.8	359.9	363.4	363.2	406.7	402.7	402.6	330.0
C (MHz)	323.0	322.0	323.5	322.4	348.5	347.9	369.9	366.1	366.0	323.9
$\|\mu_a\|^b$ (D)	3.8	3.8	3.2	3.2	1.1	1.3	2.9	3.2	3.2	1.7
$\|\mu_b\|$	0.2	0.1	0.2	0.0	0.2	0.6	1.6	1.1	1.1	0.3
$\|\mu_c\|$	0.3	0.3	1.7	1.7	2.8	2.9	2.8	3.1	3.1	2.6
ΔE^c (cm^{-1})	0.0	18.0	82.0	105.0	347.9	383.9	290.5	350.4	350.4	479.9
ΔE_0^d (cm^{-1})	0.0	2.6	40.8	44.3	110.8	126.0	136.1	161.5	161.5	225.2

[a] *A*, *B*, *C* are the rotational constants. [b] $|\mu_a|$, $|\mu_b|$, $|\mu_c|$ are the absolute values of the electric dipole moment components along the principal inertial axes *a*, *b* and *c*. [c] ΔE are the energies relative to the lowest energy isomer. [d] ΔE_0 are the zero-point corrected relative energies.

Table S2 (cont.). Predicted spectroscopic rotational parameters and relative energies for fenchone-$(H_2O)_4$ at the B3LYP-D3BJ/def2-TZVP level of theory.

Parameter	9	10	11	15	13	16	18	17	19	20
A^a (MHz)	736.6	781.4	819.9	778.5	827.4	804.1	717.2	798.1	803.2	796.7
B (MHz)	343.9	325.1	280.8	327.7	285.5	344.7	380.3	345.4	341.2	342.4
C (MHz)	319.3	305.9	270.5	306.3	263.7	301.3	333.9	302.8	299.1	300.9
$\|\mu_a\|^b$ (D)	1.6	1.4	2.3	1.6	2.3	4.1	2.8	3.2	4.1	3.3
$\|\mu_b\|$	0.7	1.6	2.5	1.6	1.9	0.1	1.3	0.1	0.1	0.1
$\|\mu_c\|$	2.6	2.2	0.1	2.3	1.6	0.1	3.0	1.9	0.1	1.8
ΔE^c (cm^{-1})	523.9	461.8	561.8	532.0	574.1	301.6	453.1	364.4	332.9	403.7
ΔE_0^d (cm^{-1})	228.3	248.2	290.4	296.5	301.6	316.9	329.4	335.6	362.8	394.6

[a] *A*, *B*, *C* are the rotational constants. [b] $|\mu_a|$, $|\mu_b|$, $|\mu_c|$ are the absolute values of the electric dipole moment components along the principal inertial axes *a*, *b* and *c*. [c] ΔE are the energies relative to the lowest energy isomer. [d] ΔE_0 are the zero-point corrected relative energies.

Table S3. Predicted spectroscopic rotational parameters and relative energies for fenchone-$(H_2O)_4$ at the MP2/6-311++G(d,p) level of theory.

Parameter	4	6	9	10	8	7	1	3	12	15
A^a (MHz)	695.6	691.5	744.8	777.2	707.8	650.0	745.0	738.6	662.9	767.7
B (MHz)	362.3	362.9	328.0	320.2	325.4	400.7	354.1	355.1	395.2	326.8
C (MHz)	346.3	346.6	308.0	302.0	318.3	366.3	318.4	320.3	359.6	305.9
$\|\mu_a\|^b$ (D)	1.0	1.0	1.5	1.3	1.6	2.8	3.5	2.9	3.0	1.4
$\|\mu_b\|$	0.1	0.5	0.7	1.7	0.2	1.6	0.2	0.2	1.4	1.7
$\|\mu_c\|$	2.8	2.9	2.5	2.0	2.7	2.7	0.2	1.3	2.9	2.2
ΔE^c (cm^{-1})	0.0	68.8	218.5	176.3	206.4	250.9	186.8	207.6	295.7	297.5
$\Delta E_0{}^d$ (cm^{-1})	0.0	54.9	155.2	176.9	192.9	227.2	255.2	259.4	274.1	277.9

[a] *A*, *B*, *C* are the rotational constants. [b] $|\mu_a|$, $|\mu_b|$, $|\mu_c|$ are the absolute values of the electric dipole moment components along the principal inertial axes *a*, *b* and *c*. [c] ΔE are the energies relative to the lowest energy isomer. [d] ΔE_0 are the zero-point corrected relative energies.

Table S3 (cont.). Predicted spectroscopic rotational parameters and relative energies for fenchone-(H_2O)$_4$ at the MP2/6-311++G(d,p) level of theory.

Parameter	2	5	14	13	11	20	19	17	16	18
A^a (MHz)	747.5	741.2	660.6	816.4	801.3	800.4	810.8	796.9	807.6	721.8
B (MHz)	352.9	353.8	380.3	283.4	280.9	340.1	338.9	341.6	340.1	373.3
C (MHz)	317.0	318.9	350.5	261.4	271.1	299.1	297.1	299.7	297.3	328.6
$\|\mu_a\|^b$ (D)	3.5	3.0	2.9	2.2	2.2	2.9	3.6	3.0	3.6	1.6
$\|\mu_b\|$	0.1	0.0	1.3	1.9	2.6	0.4	0.1	0.0	0.2	3.8
$\|\mu_c\|$	0.2	1.4	2.7	1.8	0.5	1.6	0.0	1.5	0.0	0.9
ΔE^c (cm^{-1})	218.6	245.7	430.0	370.2	387.1	467.5	467.1	486.9	490.5	513.1
ΔE_0^d (cm^{-1})	281.6	283.6	412.2	418.3	418.5	430.0	437.6	449.9	461.8	462.4

[a] *A*, *B*, *C* are the rotational constants. [b] $|\mu_a|$, $|\mu_b|$, $|\mu_c|$ are the absolute values of the electric dipole moment components along the principal inertial axes *a*, *b* and *c*. [c] ΔE are the energies relative to the lowest energy isomer. [d] ΔE_0 are the zero-point corrected relative energies.

Table S4. Experimental spectroscopic parameters of the observed isotopic species of isomer **4w-1** of fenchone-$(H_2O)_4$. Oxygen numbering is given in Figure S2.

**Parameters**[a]	$^{18}O_1$	$^{18}O_2$	$^{18}O_3$	$^{18}O_4$
A (MHz)	732.7193(74)[b]	738.155(14)	732.8445(71)	729.9037(81)
B (MHz)	343.93191(20)	336.70803(21)	343.96625(18)	343.46318(18)
C (MHz)	308.35873(20)	303.79634(27)	308.41043(19)	310.75508(18)
Δ_J (kHz)	0.02499(69)	0.02205(90)	0.02524(63)	0.02420(62)
Δ_{JK} (kHz)	[0.0169][c]	[0.0169]	[0.0169]	[0.0169]
δ_J (kHz)	[0.00246]	[0.00246]	[0.00246]	[0.00246]
σ(kHz)	5.7	5.7	5.0	5.1
N	45	37	43	47

[a] Rotational constants *A*, *B* and *C*; quartic centrifugal distortion constants Δ_J, Δ_{JK}, and δ_J; σ is the rms deviation of the fit; and *N* is the number of fitted transitions. [b] Standard error in parentheses in units of the last digit. [c] Parameters in square brackets were fixed to the values of the parent species.

Figure S2. Overlay of the MP2/6-311++G(d,p) structure of the **4w-1** isomer of fenchone-$(H_2O)_4$ with the experimental substitution coordinates of the water oxygens, represented as yellow spheres. The O···O distances are shown on the left figure in blue (experimental) and black (MP2), while the MP2 O–H···O hydrogen bonds are shown on the right figure in black.

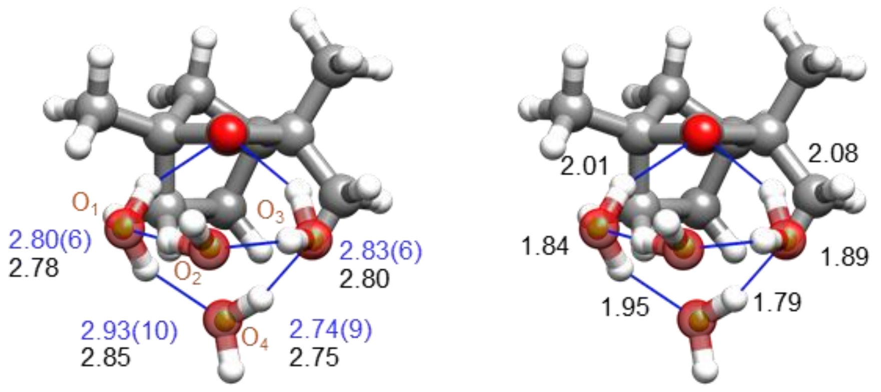

Table S5. Experimental r_s coordinates (in Å) in the principal axis frame of the water oxygen atoms of the fenchone **4w-1** complex, along with those obtained by *ab initio* calculations.

		**|*a*|**[b]	**|*b*|**	**|*c*|**
O_1	r_s	2.64146(73)[c]	1.7534(11)	0.054(37)
	MP2[a]	2.644	1.739	0.062
O_2	r_s	4.73480(51)	0.042(58)	0.6974(35)
	MP2	4.714	0.011	0.653
O_3	r_s	2.62727(72)	1.7351(11)	0.075(26)
	MP2	2.593	1.705	0.077
O_4	r_s	1.9110(10)	0.059(33)	2.09585(94)
	MP2	1.874	0.031	2.072

[a] MP2/6-311++G(d,p). [b] Absolute values of the *a*, *b*, *c* coordinates in Å. [c] Errors in parentheses, including Costain's error, in units of the last digit.

Figure S3. Overlays of the MP2/6-311++G(d,p) structures of the observed isomers of fenchone-$(H_2O)_4$ with NCI plots. The NCI isosurfaces (s = 0.5) are shown, for values of sign(λ2)ρ ranging from −0.025 to +0.025 au.

	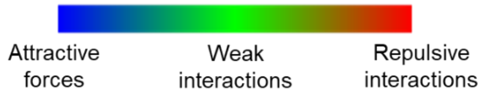	
	**4w-1**	**4w-4**
front view	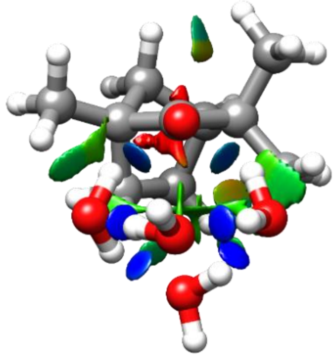	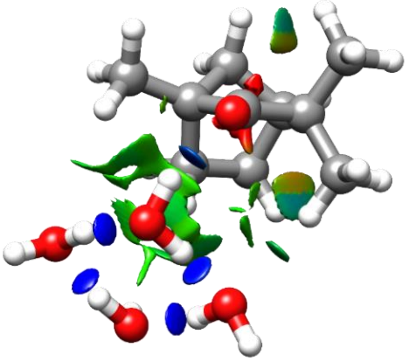
side view	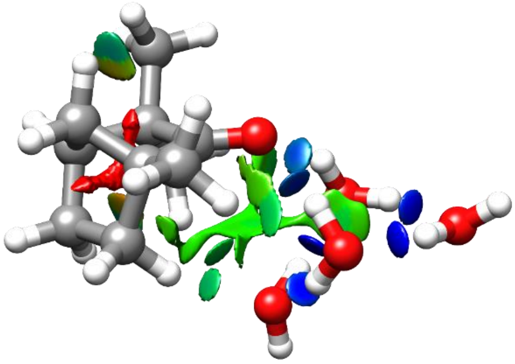	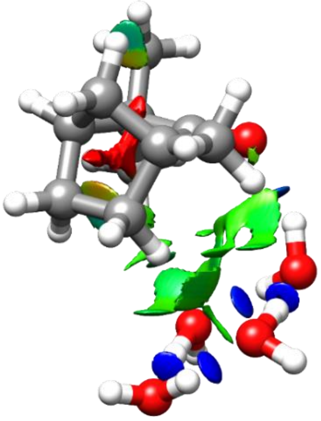
	**4w-7**	**4w-16**
front view	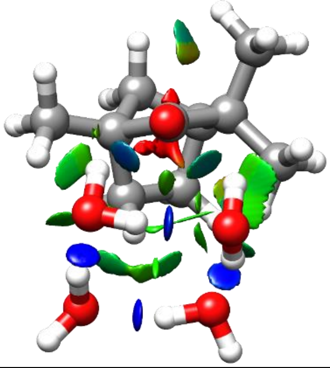	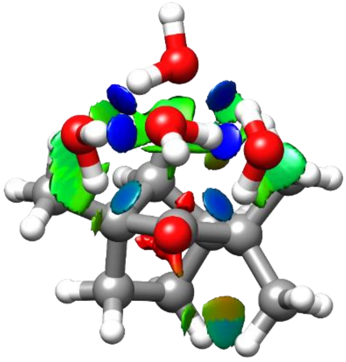
side view	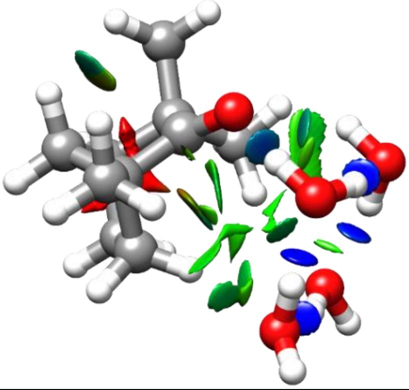	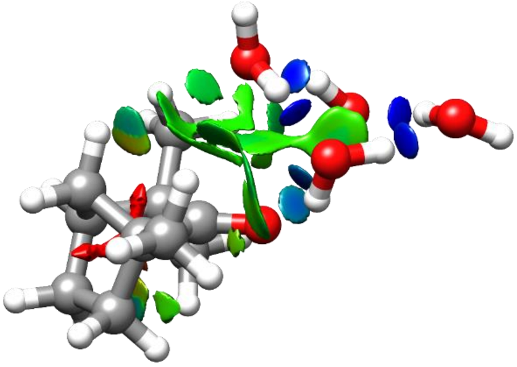

4. Fenchone-(H_2O)$_5$

Figure S4. The lower-energy isomers of fenchone-(H_2O)$_5$ within 6 kJ mol^{-1}.

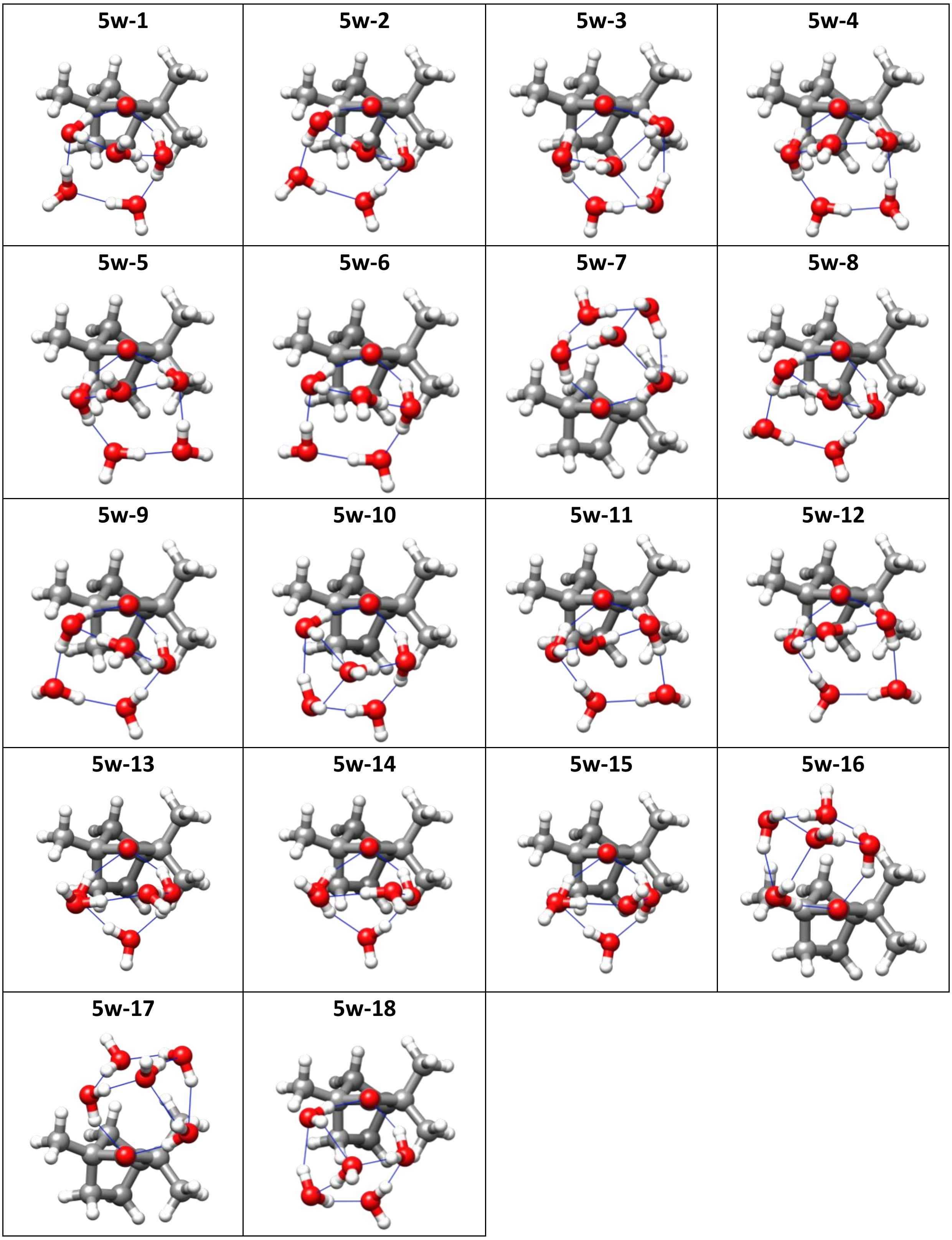

Table S6. Predicted spectroscopic rotational parameters and relative energies for fenchone-$(H_2O)_5$ at the B3LYP-D3BJ/6-311++G(d,p) level of theory.

Parameter	1	2	3	4	5	6	7	8	9	10
A^a (MHz)	581.7	593.4	626.2	571.6	577.8	580.3	673.4	589.0	585.9	627.1
B (MHz)	313.9	316.4	306.2	310.0	307.4	313.0	304.6	314.4	312.1	301.9
C (MHz)	289.7	286.0	282.7	289.0	286.6	290.2	271.1	286.4	286.4	280.1
$\|\mu_a\|^b$ (D)	3.2	3.3	3.6	2.5	4.9	4.0	3.3	5.2	4.7	3.5
$\|\mu_b\|$	0.3	0.4	1.6	0.0	0.3	1.3	1.7	0.6	1.4	1.6
$\|\mu_c\|$	1.2	1.3	0.5	2.8	1.0	2.7	0.7	0.9	2.6	0.7
ΔE^c (cm^{-1})	104.9	208.9	0.0	337.6	324.9	405.7	149.8	443.9	509.8	167.9
ΔE_0^d (cm^{-1})	0.0	70.9	96.1	146.0	177.3	195.3	196.9	257.0	284.2	288.6

[a] *A*, *B*, *C* are the rotational constants. [b] $|\mu_a|$, $|\mu_b|$, $|\mu_c|$ are the absolute values of the electric dipole moment components along the principal inertial axes *a*, *b* and *c*. [c] ΔE are the energies relative to the lowest energy isomer. [d] ΔE_0 are the zero-point corrected relative energies.

Table S6 (cont.). Predicted spectroscopic rotational parameters and relative energies for fenchone-$(H_2O)_5$ at the B3LYP-D3BJ/6-311++G(d,p) level of theory.

Parameter	11	12	13	14	15	16	17	18
A^a (MHz)	580.5	580.9	660.3	660.3	661.3	674.7	678.2	633.7
B (MHz)	305.0	303.7	266.6	266.3	266.0	294.9	302.3	298.9
C (MHz)	283.0	282.6	237.6	237.5	237.1	266.7	268.5	276.7
$\|\mu_a\|^b$ (D)	5.4	4.8	3.0	2.9	2.9	3.6	3.4	3.6
$\|\mu_b\|$	0.1	0.3	0.1	0.4	0.1	1.6	1.6	1.6
$\|\mu_c\|$	1.0	3.0	0.2	0.3	0.4	1.2	0.6	0.6
ΔE^c (cm^{-1})	467.2	491.9	427.4	434.8	482.8	379.6	409.8	490.9
ΔE_0^d (cm^{-1})	304.0	338.2	361.0	388.7	399.2	436.8	464.4	623.1

[a] *A*, *B*, *C* are the rotational constants. [b] $|\mu_a|$, $|\mu_b|$, $|\mu_c|$ are the absolute values of the electric dipole moment components along the principal inertial axes *a*, *b* and *c*. [c] ΔE are the energies relative to the lowest energy isomer. [d] ΔE_0 are the zero-point corrected relative energies.

Table S7. Predicted spectroscopic rotational parameters and relative energies for fenchone-$(H_2O)_5$ at the B3LYP-D3BJ/def2-TZVP level of theory.

Parameter	3	1	7	2	10	8	4	6	9	5
A^a (MHz)	628.9	588.5	676.7	594.2	629.9	592.1	577.4	588.5	591.3	585.0
B (MHz)	307.5	316.4	306.4	316.4	302.9	316.1	310.5	313.8	313.6	309.3
C (MHz)	283.7	290.7	272.6	287.6	280.9	287.5	288.8	289.5	287.5	286.4
$\|\mu_a\|^b$ (D)	3.4	3.0	3.1	3.1	3.4	5.2	2.5	4.3	4.7	5.1
$\|\mu_b\|$	1.7	0.3	1.8	0.2	1.6	0.4	0.0	1.4	1.3	0.4
$\|\mu_c\|$	0.6	1.2	0.7	1.2	0.7	0.7	3.2	2.7	2.7	0.7
ΔE^c (cm^{-1})	0.0	272.1	102.8	307.3	163.3	457.5	544.1	464.8	500.3	425.3
ΔE_0^d (cm^{-1})	0.0	42.8	57.5	58.6	175.4	206.1	229.8	250.0	254.6	257.0

[a] *A*, *B*, *C* are the rotational constants. [b] $|\mu_a|$, $|\mu_b|$, $|\mu_c|$ are the absolute values of the electric dipole moment components along the principal inertial axes *a*, *b* and *c*. [c] ΔE are the energies relative to the lowest energy isomer. [d] ΔE_0 are the zero-point corrected relative energies.

Table S7 (cont.). Predicted spectroscopic rotational parameters and relative energies for fenchone-$(H_2O)_5$ at the B3LYP-D3BJ/def2-TZVP level of theory.

Parameter	17	16	11	12	13	15	14	18
A^a (MHz)	678.7	679.1	589.3	594.5	662.3	663.2	661.9	635.0
B (MHz)	305.8	296.6	308.0	306.6	268.6	267.8	267.9	300.8
C (MHz)	270.4	267.9	283.0	283.1	239.5	238.7	239.0	277.9
$\|\mu_a\|^b$ (D)	3.1	3.4	5.5	4.9	2.8	2.8	2.8	3.4
$\|\mu_b\|$	1.6	1.7	0.3	1.0	0.1	0.1	0.4	1.6
$\|\mu_c\|$	0.7	1.2	0.8	3.1	0.2	0.4	0.4	0.6
ΔE^c (cm^{-1})	263.3	337.5	488.6	463.5	538.2	600.4	583.2	401.7
ΔE_0^d (cm^{-1})	271.3	298.0	341.1	354.0	371.4	420.7	435.9	456.5

[a] *A*, *B*, *C* are the rotational constants. [b] $|\mu_a|$, $|\mu_b|$, $|\mu_c|$ are the absolute values of the electric dipole moment components along the principal inertial axes *a*, *b* and *c*. [c] ΔE are the energies relative to the lowest energy isomer. [d] ΔE_0 are the zero-point corrected relative energies.

Table S8. Predicted spectroscopic rotational parameters and relative energies for fenchone-$(H_2O)_5$ at the MP2/6-311++G(d,p) level of theory.

Parameter	2	1	3	7	4	6	8	5	9	14
A^a (MHz)	590.5	581.8	621.5	670.5	570.4	575.3	586.3	577.4	581.6	656.1
B (MHz)	311.9	311.5	301.7	301.9	306.0	308.7	310.7	302.0	309.8	262.9
C (MHz)	281.7	286.9	279.3	269.2	284.0	286.1	281.9	281.1	282.3	235.2
$\|\mu_a\|^b$ (D)	3.0	2.9	3.2	2.9	2.2	3.8	4.8	4.8	4.2	2.6
$\|\mu_b\|$	0.6	0.3	1.6	1.7	0.5	1.3	0.6	0.5	1.4	0.4
$\|\mu_c\|$	1.2	1.2	0.4	0.6	2.5	2.2	0.7	0.8	2.2	0.3
ΔE^c (cm^{-1})	3.7	0.0	28.3	88.3	232.0	268.7	298.9	261.1	342.5	307.7
ΔE_0^d (cm^{-1})	0.0	1.5	111.9	120.3	142.9	204.8	210.7	229.6	232.9	293.0

^{a} A, B, C are the rotational constants. ^{b} $|\mu_a|$, $|\mu_b|$, $|\mu_c|$ are the absolute values of the electric dipole moment components along the principal inertial axes a, b and c. ^{c} ΔE are the energies relative to the lowest energy isomer. $^d\Delta E_0$ are the zero-point corrected relative energies.

Table S8 (cont.). Predicted spectroscopic rotational parameters and relative energies for fenchone-$(H_2O)_5$ at the MP2/6-311++G(d,p) level of theory.

Parameter	13	15	10	12	11	16	17	18
A^a (MHz)	656.8	657.6	622.4	578.8	581.4	675.7	674.2	632.2
B (MHz)	263.0	262.3	297.2	300.6	301.2	292.5	298.8	291.2
C (MHz)	235.3	234.6	277.3	277.8	276.8	265.3	266.5	271.5
$\|\mu_a\|^b$ (D)	2.6	2.6	3.1	4.3	5.1	3.2	2.9	3.3
$\|\mu_b\|$	0.1	0.0	1.6	1.2	0.5	1.6	1.7	1.6
$\|\mu_c\|$	0.1	0.3	0.6	2.4	0.8	1.2	0.5	0.4
ΔE^c (cm^{-1})	302.5	364.7	248.7	414.3	400.6	442.2	439.6	653.9
ΔE_0^d (cm^{-1})	297.4	319.8	335.8	354.2	371.6	459.8	461.8	724.0

^{a} A, B, C are the rotational constants. ^{b} $|\mu_a|$, $|\mu_b|$, $|\mu_c|$ are the absolute values of the electric dipole moment components along the principal inertial axes a, b and c. ^{c} ΔE are the energies relative to the lowest energy isomer. $^d\Delta E_0$ are the zero-point corrected relative energies.

Figure S5. Overlays of the MP2/6-311++G(d,p) structures of the observed isomers of fenchone-$(H_2O)_5$ with NCI plots. The NCI isosurfaces (*s* = 0.5) are shown, for values of sign(λ2)ρ ranging from −0.025 to +0.025 au.

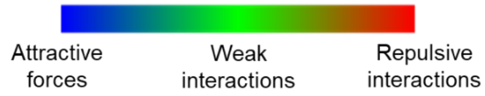

	**5w-1**	**5w-3**
front view	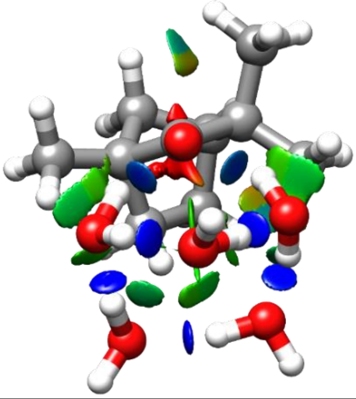	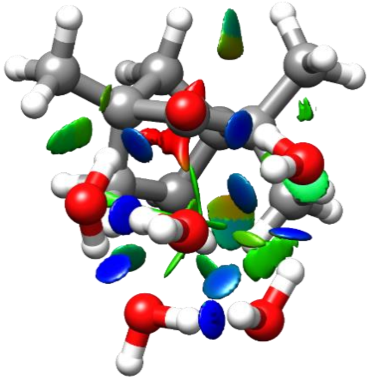
side view	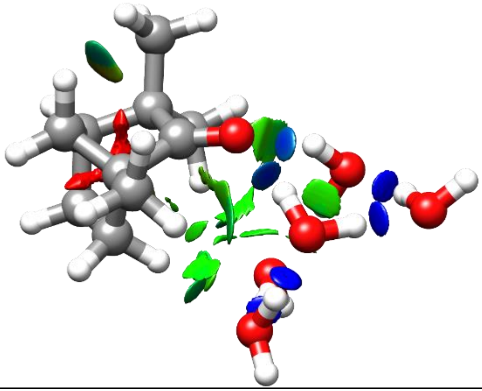	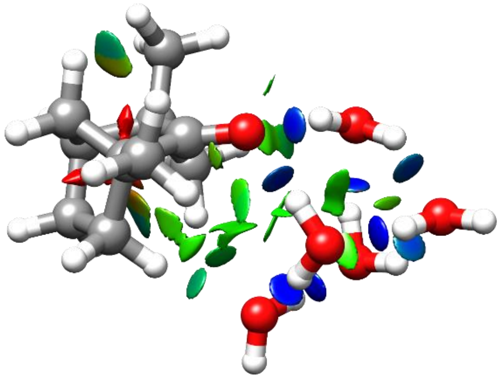
	**5w-7**	
front view	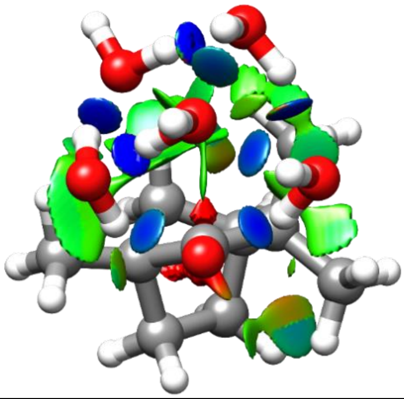	
side view	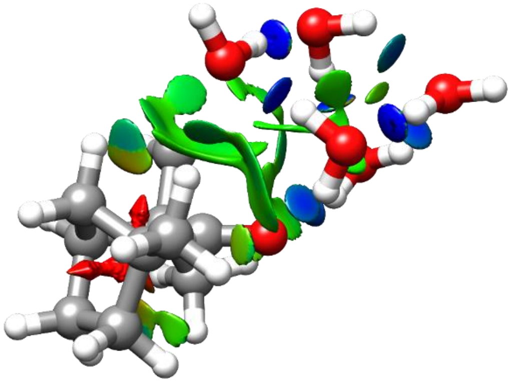	

5. Fenchone-(H_2O)$_6$

Figure S6. The lower-energy isomers of fenchone-(H_2O)$_6$ within 7 kJ mol^{-1}.

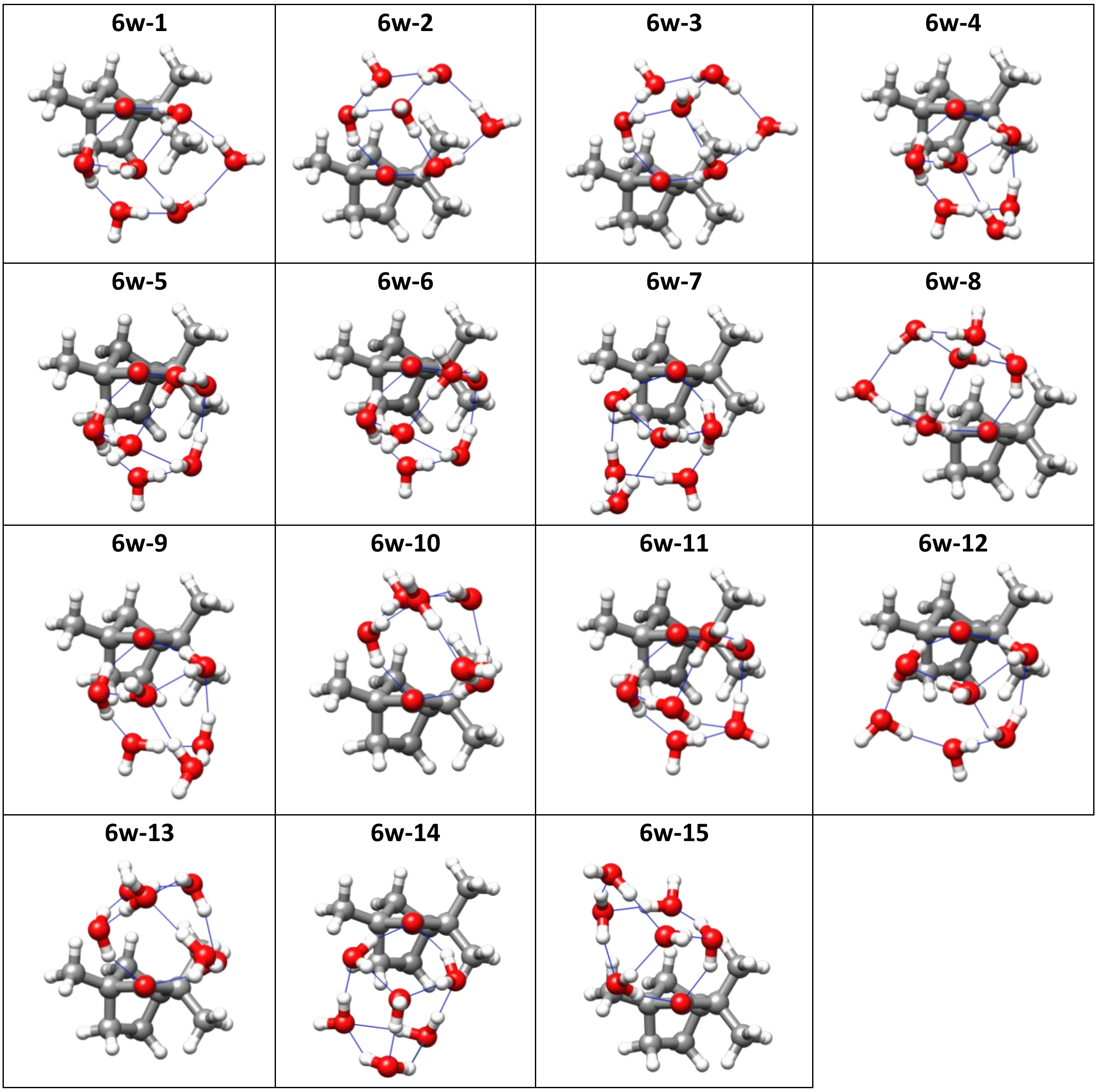

Table S9. Predicted spectroscopic rotational parameters and relative energies for fenchone-$(H_2O)_6$ at the B3LYP-D3BJ/6-311++G(d,p) level of theory.

**Parameter**	**1**	**2**	**3**	**4**	**5**	**6**	**7**	**8**	**9**	**10**
A^a (MHz)	508.0	538.6	542.0	583.1	551.9	580.2	584.0	548.9	542.4	482.3
B (MHz)	272.0	265.5	266.5	232.6	243.8	238.0	231.9	245.8	259.6	286.7
C (MHz)	242.0	232.8	231.6	220.8	227.5	225.9	220.0	228.9	226.6	253.6
$\|\mu_a\|^b$ (D)	2.8	2.8	2.8	2.6	2.1	2.1	3.1	3.2	2.9	2.7
$\|\mu_b\|$	0.1	0.1	0.1	0.9	1.9	0.9	2.8	2.6	0.3	2.4
$\|\mu_c\|$	0.3	0.6	0.7	0.8	0.1	0.5	0.3	1.4	0.5	1.4
ΔE^c (cm^{-1})	0.0	186.9	217.0	363.5	432.9	466.5	510.5	461.6	484.0	659.6
$\Delta E_0{}^d$ (cm^{-1})	0.0	117.4	164.6	303.5	368.5	382.8	397.7	416.8	421.4	421.6

[a] *A*, *B*, *C* are the rotational constants. [b] $|\mu_a|$, $|\mu_b|$, $|\mu_c|$ are the absolute values of the electric dipole moment components along the principal inertial axes *a*, *b* and *c*. [c] ΔE are the energies relative to the lowest energy isomer. [d] ΔE_0 are the zero-point corrected relative energies.

Table S9 (cont.). Predicted spectroscopic rotational parameters and relative energies for fenchone-$(H_2O)_6$ at the B3LYP-D3BJ/6-311++G(d,p) level of theory.

Parameter	11	12	13	14	15
A^a (MHz)	583.7	546.6	633.0	577.7	562.2
B (MHz)	237.5	243.8	225.1	242.1	248.7
C (MHz)	218.9	231.3	208.1	219.2	233.6
$\|\mu_a\|^b$ (D)	2.1	4.1	2.7	3.0	4.7
$\|\mu_b\|$	1.4	1.5	1.4	2.6	2.6
$\|\mu_c\|$	0.1	3.1	0.1	1.2	1.4
ΔE^c (cm^{-1})	568.4	620.5	688.1	666.5	686.3
ΔE_0^d (cm^{-1})	459.1	482.4	589.7	594.3	708.5

[a] A, B, C are the rotational constants. [b] $|\mu_a|$, $|\mu_b|$, $|\mu_c|$ are the absolute values of the electric dipole moment components along the principal inertial axes a, b and c. [c] ΔE are the energies relative to the lowest energy isomer. [d] ΔE_0 are the zero-point corrected relative energies.

Table S10. Predicted spectroscopic rotational parameters and relative energies for fenchone-$(H_2O)_6$ at the B3LYP-D3BJ/def2-TZVP level of theory.

Parameter	1	2	3	4	6	5	9	11	7	8
A^a (MHz)	511.4	541.1	543.7	591.2	583.9	555.2	547.1	587.3	592.5	549.5
B (MHz)	272.3	268.5	269.8	232.8	238.4	244.4	261.1	238.8	232.5	247.6
C (MHz)	242.7	234.1	233.2	220.9	226.3	228.0	228.2	219.3	220.4	231.0
$\|\mu_a\|^b$ (D)	2.6	2.5	2.5	2.7	2.1	2.0	2.6	1.9	3.4	3.3
$\|\mu_b\|$	0.2	0.1	0.1	1.1	0.9	1.9	0.5	1.4	2.9	2.8
$\|\mu_c\|$	0.3	0.6	0.7	0.7	0.5	0.4	0.5	0.2	0.7	1.3
ΔE^c (cm^{-1})	0.0	114.8	153.8	323.4	408.4	384.1	403.3	446.2	478.6	408.3
ΔE_0^d (cm^{-1})	0.0	42.6	78.6	243.2	337.6	341.3	359.3	360.8	363.9	385.0

[a] A, B, C are the rotational constants. [b] $|\mu_a|$, $|\mu_b|$, $|\mu_c|$ are the absolute values of the electric dipole moment components along the principal inertial axes a, b and c. [c] ΔE are the energies relative to the lowest energy isomer. [d] ΔE_0 are the zero-point corrected relative energies.

Table S10 (cont.). Predicted spectroscopic rotational parameters and relative energies for fenchone-$(H_2O)_6$ at the B3LYP-D3BJ/def2-TZVP level of theory.

Parameter	12	10	15	14	13
A^a (MHz)	549.4	484.1	563.8	579.2	638.2
B (MHz)	244.9	287.7	250.7	243.8	225.8
C (MHz)	232.6	255.0	235.0	220.9	208.6
$\|\mu_a\|^b$ (D)	3.9	2.5	4.4	3.0	2.6
$\|\mu_b\|$	1.6	2.3	2.5	2.8	1.4
$\|\mu_c\|$	3.2	1.5	1.4	1.1	0.2
ΔE^c (cm^{-1})	550.7	672.7	433.6	568.7	608.1
ΔE_0^d (cm^{-1})	436.3	459.8	480.6	515.8	526.5

[a] A, B, C are the rotational constants. [b] $|\mu_a|$, $|\mu_b|$, $|\mu_c|$ are the absolute values of the electric dipole moment components along the principal inertial axes a, b and c. [c] ΔE are the energies relative to the lowest energy isomer. [d] ΔE_0 are the zero-point corrected relative energies.

Table S11. Predicted spectroscopic rotational parameters and relative energies for fenchone-$(H_2O)_6$ at the MP2/6-311++G(d,p) level of theory.

Parameter	1	2	3	10	9	4	5	11	8	7
A^a (MHz)	502.2	528.9	535.7	476.5	539.6	586.9	543.9	581.0	541.4	587.0
B (MHz)	269.8	267.5	266.6	285.4	259.5	227.4	241.0	235.4	242.7	227.4
C (MHz)	239.7	233.5	230.5	251.8	225.9	216.4	225.5	216.4	226.5	215.8
$\|\mu_a\|^b$ (D)	2.5	2.3	2.4	2.3	2.4	2.5	1.7	1.8	2.9	2.7
$\|\mu_b\|$	0.0	0.0	0.1	2.5	0.3	1.2	1.7	1.5	2.5	2.7
$\|\mu_c\|$	0.2	0.7	0.6	1.3	0.4	0.7	0.2	0.1	1.5	0.6
ΔE^c (cm^{-1})	0.0	165.9	247.8	470.5	459.4	431.8	429.7	540.0	459.3	543.8
$\Delta E_0{}^d$ (cm^{-1})	0.0	118.3	172.7	327.9	410.4	413.7	439.4	467.9	473.8	475.6

^{a} A, B, C are the rotational constants. ^{b} $|\mu_a|$, $|\mu_b|$, $|\mu_c|$ are the absolute values of the electric dipole moment components along the principal inertial axes a, b and c. ^{c} ΔE are the energies relative to the lowest energy isomer. ^{d} ΔE_0 are the zero-point corrected relative energies.

Table S11 (cont.). Predicted spectroscopic rotational parameters and relative energies for fenchone-$(H_2O)_6$ at the MP2/6-311++G(d,p) level of theory.

Parameter	14	12	13	6	15
A^a (MHz)	578.3	537.3	635.3	580.2	560.9
B (MHz)	239.7	241.3	221.3	238.0	245.6
C (MHz)	216.6	229.6	204.5	225.9	232.1
$\|\mu_a\|^b$ (D)	2.5	3.8	2.4	1.8	3.6
$\|\mu_b\|$	2.5	1.4	1.5	1.0	2.3
$\|\mu_c\|$	1.2	3.1	0.1	0.6	1.7
ΔE^c (cm^{-1})	582.8	604.5	777.1	836.1	926.0
$\Delta E_0{}^d$ (cm^{-1})	533.1	570.6	731.5	930.6	960.0

^{a} A, B, C are the rotational constants. ^{b} $|\mu_a|$, $|\mu_b|$, $|\mu_c|$ are the absolute values of the electric dipole moment components along the principal inertial axes a, b and c. ^{c} ΔE are the energies relative to the lowest energy isomer. ^{d} ΔE_0 are the zero-point corrected relative energies.

Figure S7. Overlays of the MP2/6-311++G(d,p) structures of the observed isomers of fenchone-$(H_2O)_6$ with NCI plots. The NCI isosurfaces (*s* = 0.5) are shown, for values of sign(λ2)ρ ranging from −0.025 to +0.025 au.

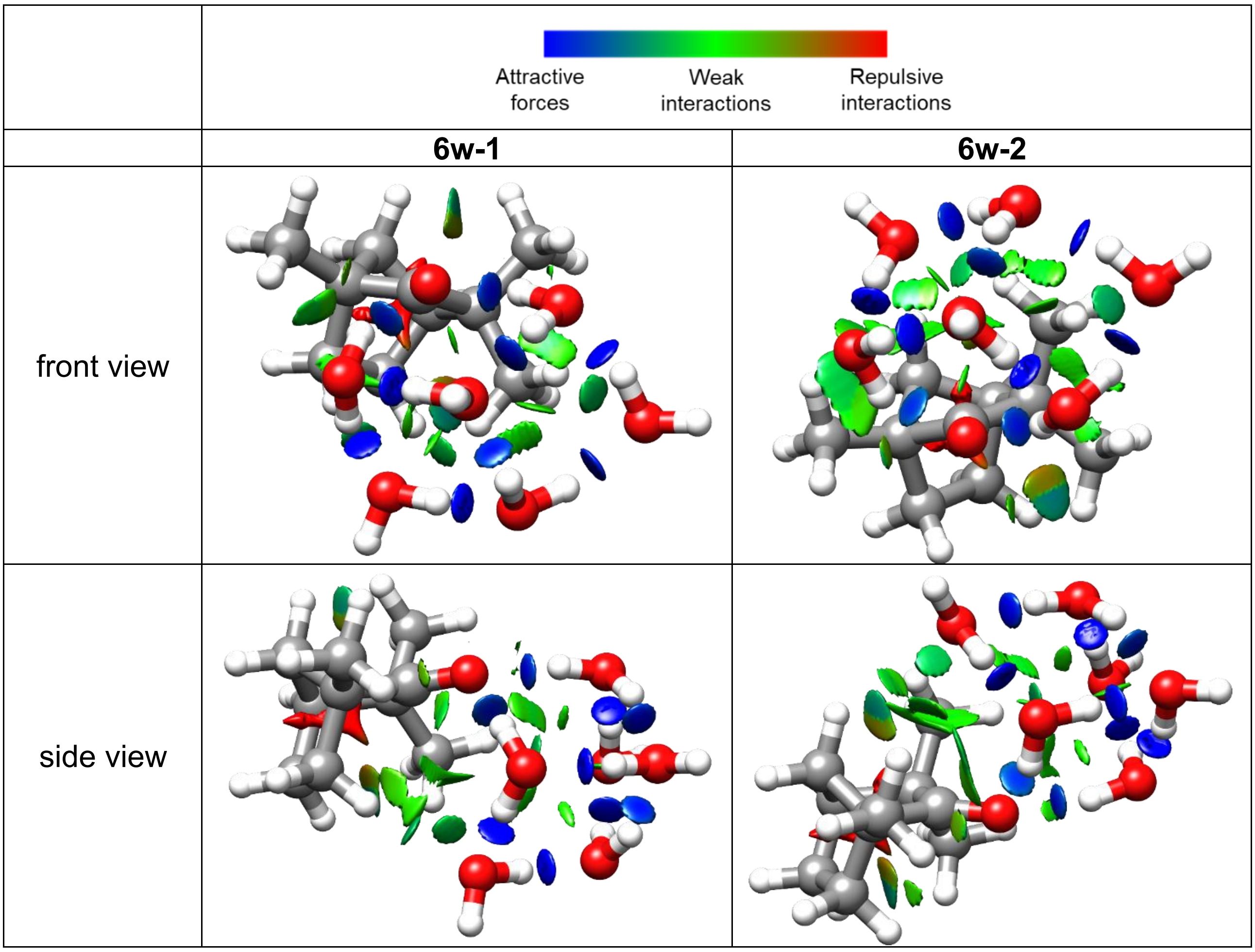

6. Fenchone-$(H_2O)_7$

Figure S8. The lower-energy isomers of fenchone-$(H_2O)_7$ within 7 kJ mol^{-1}.

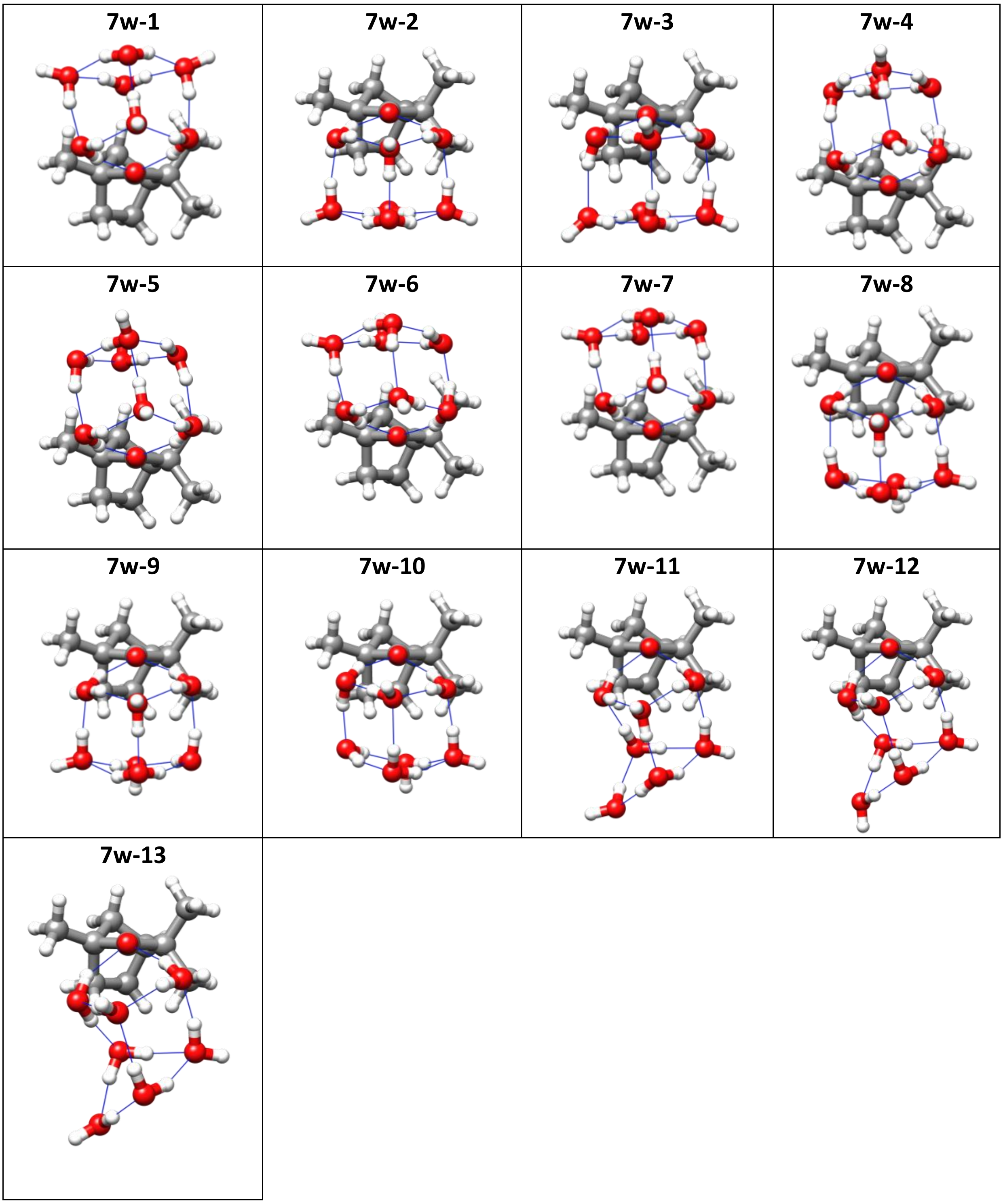

Table S12. Predicted spectroscopic rotational parameters and relative energies for fenchone-$(H_2O)_7$ at the B3LYP-D3BJ/6-311++G(d,p) level of theory.

Parameter	1	2	3	4	5	6	7	8	9	10
A^a (MHz)	503.1	476.5	477.0	501.6	506.5	489.6	494.3	467.7	466.7	462.9
B (MHz)	222.9	220.4	218.1	217.3	215.9	221.6	219.5	218.6	220.0	221.0
C (MHz)	204.0	206.5	206.0	205.4	203.1	206.7	204.4	209.5	211.1	213.0
$\|\mu_a\|^b$ (D)	2.3	2.6	2.7	1.6	1.5	5.1	4.7	5.0	4.8	5.2
$\|\mu_b\|$	0.2	0.1	0.1	0.2	0.1	3.5	3.9	3.9	3.9	3.4
$\|\mu_c\|$	0.6	1.1	1.0	0.2	0.6	0.2	0.1	0.3	0.2	0.3
ΔE^c (cm^{-1})	0.0	188.5	276.3	297.8	300.9	473.6	474.9	545.8	532.9	554.4
ΔE_0^d (cm^{-1})	0.0	170.8	251.1	323.9	338.2	520.4	522.3	546.7	554.2	554.6

[a] A, B, C are the rotational constants. [b] $|\mu_a|$, $|\mu_b|$, $|\mu_c|$ are the absolute values of the electric dipole moment components along the principal inertial axes a, b and c. [c] ΔE are the energies relative to the lowest energy isomer. [d] ΔE_0 are the zero-point corrected relative energies.

Table S12 (cont.). Predicted spectroscopic rotational parameters and relative energies for fenchone-$(H_2O)_7$ at the B3LYP-D3BJ/6-311++G(d,p) level of theory.

Parameter	11	12	13
A^a (MHz)	489.4	486.7	485.2
B (MHz)	201.0	202.0	201.8
C (MHz)	189.6	191.7	191.9
$\|\mu_a\|^b$ (D)	5.1	4.8	5.0
$\|\mu_b\|$	0.8	1.0	0.6
$\|\mu_c\|$	0.1	1.4	0.5
ΔE^c (cm^{-1})	556.6	595.0	629.0
ΔE_0^d (cm^{-1})	571.5	597.8	613.0

[a] A, B, C are the rotational constants. [b] $|\mu_a|$, $|\mu_b|$, $|\mu_c|$ are the absolute values of the electric dipole moment components along the principal inertial axes a, b and c. [c] ΔE are the energies relative to the lowest energy isomer. [d] ΔE_0 are the zero-point corrected relative energies.

Table S13. Predicted spectroscopic rotational parameters and relative energies for fenchone-$(H_2O)_7$ at the B3LYP-D3BJ/def2-TZVP level of theory.

Parameter	1	5	2	4	3	7	6	11	10	9
A^a (MHz)	505.0	508.2	478.6	506.1	479.9	496.9	494.4	492.0	465.6	467.1
B (MHz)	224.1	218.0	221.4	218.6	218.5	221.7	221.9	202.7	221.5	222.1
C (MHz)	204.9	204.5	207.4	205.7	206.5	205.6	206.9	191.4	213.8	213.1
$\|\mu_a\|^b$ (D)	2.1	1.3	2.4	1.4	2.5	4.5	5.0	4.9	5.1	4.5
$\|\mu_b\|$	0.2	0.1	0.1	0.3	0.1	3.9	3.4	1.0	3.4	3.8
$\|\mu_c\|$	0.6	0.5	1.1	0.2	1.0	0.1	0.2	0.4	0.4	0.0
ΔE^c (cm^{-1})	0.0	199.7	260.7	236.6	313.4	338.0	346.8	458.7	480.9	476.0
ΔE_0^d (cm^{-1})	0.0	242.3	242.7	249.8	286.6	379.5	392.9	441.8	466.8	477.1

[a] A, B, C are the rotational constants. [b] $|\mu_a|$, $|\mu_b|$, $|\mu_c|$ are the absolute values of the electric dipole moment components along the principal inertial axes a, b and c. [c] ΔE are the energies relative to the lowest energy isomer. [d] ΔE_0 are the zero-point corrected relative energies.

Table S13 (cont.). Predicted spectroscopic rotational parameters and relative energies for fenchone-$(H_2O)_7$ at the B3LYP-D3BJ/def2-TZVP level of theory.

Parameter	8	13	12
A^a (MHz)	470.3	487.7	488.1
B (MHz)	220.4	203.3	203.0
C (MHz)	210.4	193.5	193.4
$\|\mu_a\|^b$ (D)	4.7	5.0	4.7
$\|\mu_b\|$	3.9	0.7	1.2
$\|\mu_c\|$	0.2	0.8	1.4
ΔE^c (cm^{-1})	477.6	577.7	567.1
ΔE_0^d (cm^{-1})	477.4	539.5	558.3

[a] A, B, C are the rotational constants. [b] $|\mu_a|$, $|\mu_b|$, $|\mu_c|$ are the absolute values of the electric dipole moment components along the principal inertial axes a, b and c. [c] ΔE are the energies relative to the lowest energy isomer. [d] ΔE_0 are the zero-point corrected relative energies.

Table S14. Predicted spectroscopic rotational parameters and relative energies for fenchone-$(H_2O)_7$ at the MP2/6-311++G(d,p) level of theory.

Parameter	1	2	3	4	5	7	6	12	10	11
A^a (MHz)	499.9	469.8	476.0	496.0	503.6	490.4	483.9	480.3	453.6	482.7
B (MHz)	221.5	217.7	214.5	214.9	213.1	216.9	220.5	199.9	220.9	198.0
C (MHz)	202.0	204.4	201.8	204.2	200.4	201.6	204.6	191.8	212.7	189.1
$\|\mu_a\|^b$ (D)	1.9	2.2	2.2	1.2	1.3	4.4	4.6	4.1	4.5	4.5
$\|\mu_b\|$	0.1	0.0	0.2	0.2	0.1	3.9	3.5	1.2	3.3	0.7
$\|\mu_c\|$	0.4	1.1	0.8	0.2	0.4	0.2	0.5	1.0	0.9	0.3
ΔE^c (cm^{-1})	0.0	239.6	335.9	411.2	459.8	609.1	652.2	672.9	679.4	703.6

[a] A, B, C are the rotational constants. [b] $|\mu_a|$, $|\mu_b|$, $|\mu_c|$ are the absolute values of the electric dipole moment components along the principal inertial axes a, b and c. [c] ΔE are the energies relative to the lowest energy isomer.

Table S14 (cont.). Predicted spectroscopic rotational parameters and relative energies for fenchone-$(H_2O)_7$ at the MP2/6-311++G(d,p) level of theory.

Parameter	13	8	9
A^a (MHz)	479.1	459.0	458.8
B (MHz)	199.1	216.1	217.5
C (MHz)	191.9	209.0	209.9
$\|\mu_a\|^b$ (D)	4.4	4.7	4.3
$\|\mu_b\|$	0.5	3.9	3.8
$\|\mu_c\|$	0.6	0.2	0.3
ΔE^c (cm^{-1})	704.8	705.0	714.1

[a] A, B, C are the rotational constants. [b] $|\mu_a|$, $|\mu_b|$, $|\mu_c|$ are the absolute values of the electric dipole moment components along the principal inertial axes a, b and c. [c] ΔE are the energies relative to the lowest energy isomer.

Figure S9. Overlays of the MP2/6-311++G(d,p) structures of the observed isomers of fenchone$(H_2O)_7$ with NCI plots. The NCI isosurfaces (s = 0.5) are shown, for values of sign(λ2)ρ ranging from −0.025 to +0.025 au.

	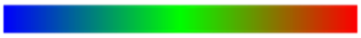<br>Attractive forces<br>Weak interactions<br>Repulsive interactions
	**7w-1**
front view	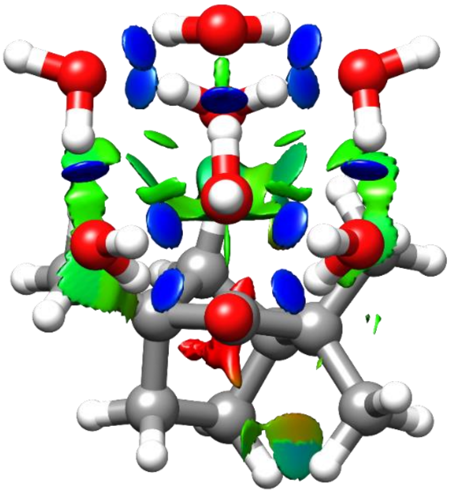
side view	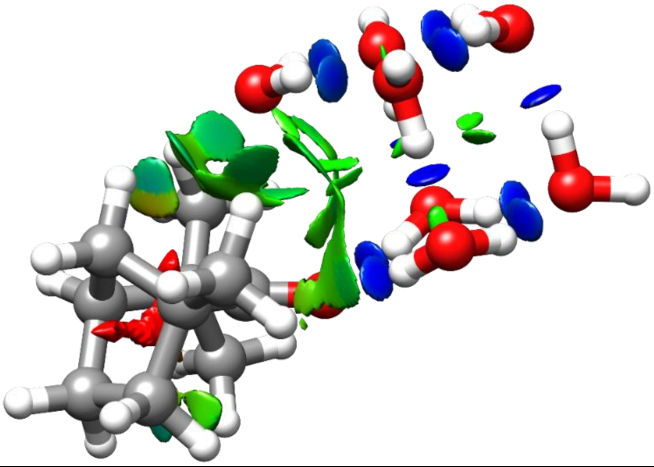

7. SAPT and BSSE energy calculations

Table S15. Energy decomposition analysis calculated at the SAPT2+/aug-cc-pDVZ level of theory for the observed isomers of fenchone-$(H_2O)_{4-7}$ on MP2/6-311++G(d,p) geometries, and BSSE corrected interaction energies at the MP2/6-311++G(d,p) level. All values in kJ mol^{-1}.

	ΔE_{elec}	ΔE_{exch}	ΔE_{ind}	ΔE_{disp}	ΔE_{total}	BSSE
**4w-1**	-71.2	81.3	-24.2	-42.1	-56.2	-125.9
**4w-4**	-42.7	58.7	-15.0	-36.6	-35.7	-124.5
**4w-7**	-56.3	64.1	-17.7	-39.2	-49.3	-123.2
**4w-16**	-65.2	73.9	-20.9	-41.1	-53.2	-123.1
**5w-1**	-75.5	87.6	-26.9	-46.5	-61.3	-164.9
**5w-3**	-80.8	94.0	-29.4	-45.9	-62.1	-165.8
**5w-7**	-79.1	93.4	-27.5	-49.2	-62.4	-164.4
**6w-1**	-83.1	97.7	-31.4	-49.2	-66.0	-206.7
**6w-3**	-79.1	92.1	-28.4	-50.1	-65.6	-205.6
**7w-1**	-103.1	126.1	-42.4	-60.6	-80.0	-249.6

8. Measured rotational transitions

8.1. Fenchone-$(H_2O)_4$

Table S16. Measured frequencies (ν_{obs}) and residuals ($\nu_{obs} - \nu_{cal}$) of the rotational transitions of isomer **4w-1** of fenchone-$(H_2O)_4$.

J'	*K'*$_{-1}$	*K'*$_{+1}$	*J"*	*K"*$_{-1}$	*K"*$_{+1}$	ν_{obs}	$\nu_{obs} - \nu_{cal}$
4	1	4	3	1	3	2563.1951	0.0020
4	0	4	3	0	3	2615.6147	-0.0007
4	2	3	3	2	2	2635.8246	-0.0012
4	3	2	3	3	1	2641.8228	0.0050
4	3	1	3	3	0	2642.3660	0.0070
4	2	2	3	2	1	2657.7851	-0.0004
4	1	3	3	1	2	2702.9375	-0.0024
5	1	5	4	1	4	3200.3485	0.0005
5	0	5	4	0	4	3254.5201	-0.0003
5	2	4	4	2	3	3291.9692	0.0000
5	4	2	4	4	1	3301.9783	0.0027
5	4	1	4	4	0	3301.9783	0.0027
5	3	3	4	3	2	3303.7938	0.0002
5	3	2	4	3	1	3305.6755	-0.0015
5	2	3	4	2	2	3334.3919	-0.0009
5	1	4	4	1	3	3373.8210	-0.0026
6	1	6	5	1	5	3835.5136	0.0004
6	0	6	5	0	5	3886.1199	-0.0011
6	2	5	5	2	4	3946.2586	0.0001
6	5	1	5	5	0	3961.9851	-0.0005
6	5	2	5	5	1	3961.9851	-0.0005
6	4	3	5	4	2	3963.9585	-0.0091
6	4	2	5	4	1	3964.0929	0.0173
6	3	3	5	3	2	3971.3136	-0.0011
6	2	4	5	2	3	4016.3850	0.0006
6	1	5	5	1	4	4040.9692	-0.0024
7	1	7	6	1	6	4468.6709	-0.0007
7	0	7	6	0	6	4511.9404	-0.0015
7	2	6	6	2	5	4598.3605	-0.0007
7	6	1	6	6	0	4622.0336	-0.0076
7	6	2	6	6	1	4622.0336	-0.0076
7	5	2	6	5	1	4623.7335	-0.0003
7	5	3	6	5	2	4623.7335	-0.0003
7	4	4	6	4	3	4626.7984	0.0001
7	4	3	6	4	2	4627.1725	0.0161
7	3	5	6	3	4	4629.2036	-0.0007
7	3	4	6	3	3	4640.1909	-0.0012
7	2	5	6	2	4	4701.7986	-0.0005
7	1	6	6	1	5	4703.1756	-0.0025
8	1	8	7	1	7	5099.9383	0.0003
8	0	8	7	0	7	5134.2430	-0.0012
8	2	7	7	2	6	5247.9849	-0.0007
8	7	1	7	7	0	5282.1303	0.0139
8	7	2	7	7	1	5282.1303	0.0139
8	6	2	7	6	1	5283.6137	0.0040
8	6	3	7	6	2	5283.6137	0.0040
8	5	3	7	5	2	5286.1403	0.0006
8	5	4	7	5	3	5286.1403	0.0006
8	4	5	7	4	4	5290.5199	0.0000
8	4	4	7	4	3	5291.4969	0.0000
8	3	6	7	3	5	5291.9744	-0.0017
8	3	5	7	3	4	5313.3466	0.0000
8	1	7	7	1	6	5359.1130	-0.0029
8	2	6	7	2	5	5387.8792	-0.0017
9	1	9	8	1	8	5729.5268	0.0013
9	0	9	8	0	8	5755.1095	0.0004

Table S16 (cont.). Measured frequencies (ν_{obs}) and residuals ($\nu_{obs} - \nu_{cal}$) in MHz of the rotational transitions of isomer **4w-1** of fenchone-$(H_2O)_4$.

J'	*K'*$_{-1}$	*K'*$_{+1}$	*J"*	*K"*$_{-1}$	*K"*$_{+1}$	ν_{obs}	$\nu_{obs} - \nu_{cal}$
9	2	8	8	2	7	5894.8991	-0.0012
9	8	1	8	8	0	5942.1991	-0.0004
9	8	2	8	8	1	5942.1991	-0.0004
9	7	2	8	7	1	5943.5633	-0.0002
9	7	3	8	7	2	5943.5633	-0.0002
9	6	3	8	6	2	5945.6957	0.0048
9	6	4	8	6	3	5945.6957	0.0048
9	5	5	8	5	4	5949.2467	-0.0235
9	5	4	8	5	3	5949.3494	0.0151
9	3	7	8	3	6	5954.1661	-0.0013
9	4	6	8	4	5	5955.1366	0.0007
9	4	5	8	4	4	5957.4522	-0.0011
9	3	6	8	3	5	5991.7277	-0.0011
9	1	8	8	1	7	6007.5344	-0.0018
9	2	7	8	2	6	6071.9603	-0.0031
10	1	10	9	1	9	6357.6885	-0.0146
10	0	10	9	0	9	6375.8942	-0.0002
10	2	9	9	2	8	6538.9562	0.0013
10	9	1	9	9	0	6602.2817	-0.0026
10	9	2	9	9	1	6602.2817	-0.0026
10	8	2	9	8	1	6603.5589	0.0011
10	8	3	9	8	2	6603.5589	0.0011
10	7	3	9	7	2	6605.4268	-0.0022
10	7	4	9	7	3	6605.4268	-0.0022
10	6	4	9	6	3	6608.3501	-0.0011
10	6	5	9	6	4	6608.3501	-0.0011
10	5	6	9	5	5	6613.2364	0.0012
10	5	5	9	5	4	6613.4110	-0.0027
10	3	8	9	3	7	6615.2359	0.0000
10	4	7	9	4	6	6620.5683	0.0006
10	4	6	9	4	5	6625.5044	-0.0012
10	1	9	9	1	8	6647.6454	0.0087
10	3	7	9	3	6	6675.8231	-0.0039
10	2	8	9	2	7	6751.9758	-0.0023
11	0	11	10	0	10	6997.2297	0.0016
11	2	10	10	2	9	7180.0995	0.0019
11	8	3	10	8	2	7265.2632	-0.0055
11	8	4	10	8	3	7265.2632	-0.0055
11	6	5	10	6	4	7271.6586	0.0001
11	6	6	10	6	5	7271.6586	0.0001
11	3	9	10	3	8	7274.6384	0.0010
11	5	7	10	5	6	7278.0992	0.0046
11	5	6	10	5	5	7278.5356	-0.0010
11	1	10	10	1	9	7279.5077	-0.0018
11	4	8	10	4	7	7286.6378	0.0003
11	4	7	10	4	6	7296.2881	0.0012
11	3	8	10	3	7	7365.2106	0.0019
11	2	9	10	2	8	7426.4296	-0.0006
12	1	12	11	1	11	7610.9435	-0.0004
12	0	12	11	0	11	7619.2681	0.0036
12	2	11	11	2	10	7818.3879	0.0033
12	1	11	11	1	10	7904.3939	-0.0014
12	8	4	11	8	3	7927.3681	0.0000
12	8	5	11	8	4	7927.3681	0.0000
12	7	5	11	7	4	7930.6043	-0.0032
12	7	6	11	7	5	7930.6043	-0.0032
12	6	6	11	6	5	7935.6823	-0.0010
12	6	7	11	6	6	7935.6823	-0.0010

Table S16 (cont.). Measured frequencies (ν_{obs}) and residuals ($\nu_{obs} - \nu_{cal}$) in MHz of the rotational transitions of isomer **4w-1** of fenchone-$(H_2O)_4$.

J'	K'_{-1}	K'_{+1}	*J"*	$K"_{-1}$	$K"_{+1}$	ν_{obs}	$\nu_{obs} - \nu_{cal}$
12	5	8	11	5	7	7943.8980	0.0025
12	5	7	11	5	6	7944.8949	0.0014
12	4	9	11	4	8	7953.0599	0.0014
12	4	8	11	4	7	7970.5905	0.0026

Table S17. Measured frequencies (ν_{obs}) and residuals ($\nu_{obs} - \nu_{cal}$) of the rotational transitions of the $^{18}O_1$ isotopologue of isomer **4w-1** of fenchone-$(H_2O)_4$.

J'	K'_{-1}	K'_{+1}	*J''*	K''_{-1}	K''_{+1}	ν_{obs}	$\nu_{obs} - \nu_{cal}$
5	1	5	4	1	4	3163.2616	-0.0040
5	0	5	4	0	4	3217.5581	-0.0106
5	2	4	4	2	3	3256.2578	-0.0042
5	3	2	4	3	1	3270.4847	-0.0007
6	1	6	5	1	5	3790.8676	0.0036
6	0	6	5	0	5	3841.2821	0.0060
6	3	4	5	3	3	3924.0311	0.0088
6	3	3	5	3	2	3929.2749	-0.0030
6	1	5	5	1	4	3999.0421	-0.0003
7	0	7	6	0	6	4459.1995	0.0050
7	2	6	6	2	5	4548.0153	0.0119
7	3	5	6	3	4	4579.8514	0.0042
7	3	4	6	3	3	4591.4560	-0.0005
7	1	6	6	1	5	4653.8016	0.0022
8	1	8	7	1	7	5040.0352	0.0088
8	0	8	7	0	7	5073.6773	-0.0028
8	2	7	7	2	6	5190.1740	-0.0082
8	3	6	7	3	5	5235.5404	-0.0075
8	3	5	7	3	4	5258.1060	0.0080
8	1	7	7	1	6	5302.0253	-0.0048
8	2	6	7	2	5	5333.7235	0.0030
9	1	9	8	1	8	5661.9409	-0.0038
9	0	9	8	0	8	5686.8352	-0.0056
9	2	8	8	2	7	5829.5655	-0.0009
9	3	7	8	3	6	5890.6151	0.0067
9	3	6	8	3	5	5930.1680	0.0033
9	1	8	8	1	7	5942.4609	-0.0012
9	2	7	8	2	6	6010.7555	-0.0100
10	1	10	9	1	9	6282.4434	-0.0017
10	0	10	9	0	9	6300.0040	-0.0051
10	1	9	9	1	8	6574.3336	0.0035
10	3	7	9	3	6	6608.0851	-0.0100
10	2	8	9	2	7	6683.4787	0.0036
11	1	11	10	1	10	6901.8167	-0.0041
11	0	11	10	0	10	6913.7705	-0.0034
11	2	10	10	2	9	7099.4652	-0.0012
11	3	9	10	3	8	7196.5531	-0.0016
11	1	10	10	1	9	7197.8434	-0.0006
11	3	8	10	3	7	7291.3319	0.0087
11	2	9	10	2	8	7350.3459	0.0003
12	1	12	11	1	11	7520.3502	0.0066
12	2	11	11	2	10	7730.0175	0.0074
12	1	11	11	1	10	7814.4139	-0.0019
12	3	10	11	3	9	7846.3671	0.0046
12	3	9	11	3	8	7978.0257	-0.0088

Table S18. Measured frequencies (ν_{obs}) and residuals ($\nu_{obs} - \nu_{cal}$) of the rotational transitions of the $^{18}O_2$ isotopologue of isomer **4w-1** of fenchone-$(H_2O)_4$.

J'	K'_{-1}	K'_{+1}	*J''*	K''_{-1}	K''_{+1}	ν_{obs}	$\nu_{obs} - \nu_{cal}$
5	1	5	4	1	4	3112.4599	0.0021
5	0	5	4	0	4	3165.5567	-0.0096
5	3	3	4	3	2	3208.4698	0.0088
5	3	2	4	3	1	3209.9734	0.0163
6	1	6	5	1	5	3730.6366	0.0015
6	0	6	5	0	5	3781.4340	0.0060
6	3	4	5	3	3	3851.7741	-0.0084
6	3	3	5	3	2	3855.7288	-0.0083
6	1	5	5	1	4	3924.1413	-0.0044
7	1	7	6	1	6	4346.9951	-0.0060
7	0	7	6	0	6	4391.6588	0.0020
7	3	5	6	3	4	4495.4493	-0.0013
7	3	4	6	3	3	4504.2198	0.0018
7	2	5	6	2	4	4560.0729	0.0027
7	2	5	6	2	4	4560.0729	0.0027
7	1	6	6	1	5	4568.6031	-0.0024
8	1	8	7	1	7	4961.6282	0.0012
8	0	8	7	0	7	4998.1266	0.0044
8	2	7	7	2	6	5100.5149	-0.0133
8	3	5	7	3	4	5156.2795	-0.0011
8	1	7	7	1	6	5207.7678	0.0008
9	1	9	8	1	8	5574.6748	0.0006
9	0	9	8	0	8	5602.7508	0.0041
9	2	8	8	2	7	5730.1950	0.0024
9	3	6	8	3	5	5812.7970	-0.0008
9	1	8	8	1	7	5840.5029	0.0003
9	2	7	8	2	6	5890.2259	0.0035
10	1	10	9	1	9	6186.3550	-0.0072
10	2	9	9	2	8	6357.3345	0.0085
10	3	7	9	3	6	6474.3725	-0.0044
10	2	8	9	2	7	6551.7128	0.0016
11	1	11	10	1	10	6796.9372	0.0029
11	0	11	10	0	10	6811.4624	-0.0044
11	3	9	10	3	8	7066.1664	-0.0070
11	3	8	10	3	7	7141.0013	0.0037
12	1	12	11	1	11	7406.6322	0.0035
12	0	12	11	0	11	7416.6016	-0.0003

Table S19. Measured frequencies (ν_{obs}) and residuals ($\nu_{obs} - \nu_{cal}$) of the rotational transitions of the $^{18}O_3$ isotopologue of isomer **4w-1** of fenchone-$(H_2O)_4$.

J'	K'_{-1}	K'_{+1}	*J"*	K''_{-1}	K''_{+1}	ν_{obs}	$\nu_{obs} - \nu_{cal}$
5	0	5	4	0	4	3218.0491	0.0022
5	2	4	4	2	3	3256.6899	-0.0083
5	2	3	4	2	2	3300.4952	-0.0009
5	1	4	4	1	3	3339.5387	0.0016
6	0	6	5	0	5	3841.8665	-0.0005
6	2	5	5	2	4	3903.7970	0.0050
6	3	4	5	3	3	3924.5276	-0.0002
6	3	3	5	3	2	3929.7747	0.0010
6	2	4	5	2	3	3976.0464	-0.0005
6	1	5	5	1	4	3999.5432	0.0098
7	1	7	6	1	6	4417.0941	-0.0032
7	2	6	6	2	5	4548.6282	0.0019
7	3	5	6	3	4	4580.4346	-0.0016
7	3	4	6	3	3	4592.0216	-0.0029
7	2	5	6	2	4	4654.9468	0.0026
8	1	8	7	1	7	5040.8274	0.0077
8	2	7	7	2	6	5190.9088	0.0063
8	3	6	7	3	5	5236.2215	-0.0002
8	1	7	7	1	6	5302.7210	-0.0034
8	2	6	7	2	5	5334.3174	-0.0001
9	1	9	8	1	8	5662.8436	0.0005
9	0	9	8	0	8	5687.7548	-0.0061
9	2	8	8	2	7	5830.3830	-0.0039
9	3	7	8	3	6	5891.3713	0.0025
9	3	6	8	3	5	5930.8612	0.0034
9	1	8	8	1	7	5943.2723	0.0004
9	2	7	8	2	6	6011.4460	0.0002
10	1	10	9	1	9	6283.4465	-0.0024
10	0	10	9	0	9	6301.0293	-0.0034
10	2	9	9	2	8	6466.9330	0.0009
10	3	8	9	3	7	6545.3143	-0.0001
10	1	9	9	1	8	6575.2582	-0.0053
10	2	8	9	2	7	6684.2429	-0.0047
11	2	10	10	2	9	7100.4990	0.0039
11	3	9	10	3	8	7197.5071	0.0099
11	1	10	10	1	9	7198.8951	-0.0099
11	3	8	10	3	7	7292.1095	-0.0149
11	2	9	10	2	8	7351.2212	0.0017
12	1	12	11	1	11	7521.5554	-0.0022
12	2	11	11	2	10	7731.1530	0.0069
12	1	11	11	1	10	7815.6068	0.0041
12	3	10	11	3	9	7847.4028	0.0015
12	3	9	11	3	8	7978.8987	0.0031

Table S20. Measured frequencies (ν_{obs}) and residuals ($\nu_{obs} - \nu_{cal}$) of the rotational transitions of the $^{18}O_4$ isotopologue of isomer **4w-1** of fenchone-$(H_2O)_4$.

J'	K'_{-1}	K'_{+1}	*J''*	K''_{-1}	K''_{+1}	ν_{obs}	$\nu_{obs} - \nu_{cal}$
5	1	5	4	1	4	3181.3671	0.0018
5	0	5	4	0	4	3233.3405	0.0042
5	3	3	4	3	2	3277.1570	0.0029
5	3	2	4	3	1	3278.7435	0.0095
5	2	3	4	2	2	3304.3866	0.0042
6	1	6	5	1	5	3813.2505	0.0032
6	0	6	5	0	5	3862.5438	0.0028
6	2	5	5	2	4	3916.3610	0.0026
6	3	4	5	3	3	3934.2190	-0.0046
6	3	3	5	3	2	3938.3989	0.0013
6	1	5	5	1	4	4005.3171	0.0001
7	1	7	6	1	6	4443.3032	0.0008
7	0	7	6	0	6	4486.2145	0.0018
7	2	6	6	2	5	4564.1179	-0.0006
7	3	5	6	3	4	4591.6210	0.0023
7	3	4	6	3	3	4600.8595	-0.0040
7	2	5	6	2	4	4657.1176	0.0022
7	1	6	6	1	5	4662.9931	-0.0110
8	0	8	7	0	7	5106.3019	-0.0023
8	2	7	7	2	6	5209.6684	-0.0055
8	3	6	7	3	5	5249.0018	-0.0045
8	3	5	7	3	4	5267.0394	-0.0013
8	1	7	7	1	6	5315.2235	0.0039
8	2	6	7	2	5	5336.2848	-0.0035
9	1	9	8	1	8	5698.3515	-0.0047
9	0	9	8	0	8	5724.7353	-0.0092
9	2	8	8	2	7	5852.8011	-0.0071
9	3	7	8	3	6	5905.9784	0.0141
9	3	6	8	3	5	5937.7993	-0.0085
9	1	8	8	1	7	5960.8293	0.0061
9	2	7	8	2	6	6014.1078	-0.0020
10	1	10	9	1	9	6323.7674	0.0084
10	0	10	9	0	9	6342.8881	-0.0031
10	2	9	9	2	8	6493.3723	0.0010
10	3	8	9	3	7	6562.0204	0.0008
10	1	9	9	1	8	6598.9820	0.0005
10	3	7	9	3	6	6613.7249	0.0008
11	1	11	10	1	10	6948.0751	0.0027
11	0	11	10	0	10	6961.4328	-0.0081
11	2	10	10	2	9	7131.3044	0.0097
11	3	9	10	3	8	7216.6895	0.0016
11	3	8	10	3	7	7294.6385	-0.0059
11	2	9	10	2	8	7358.3938	-0.0006
12	1	12	11	1	11	7571.5379	0.0000
12	2	11	11	2	10	7766.6062	0.0047
12	1	11	11	1	10	7853.3303	-0.0041
12	3	9	11	3	8	7979.4256	0.0031

Table S21. Measured frequencies (ν_{obs}) and residuals ($\nu_{obs} - \nu_{cal}$) of the rotational transitions of isomer **4w-4** of fenchone-$(H_2O)_4$.

J'	K'_{-1}	K'_{+1}	*J"*	K''_{-1}	K''_{+1}	ν_{obs}	$\nu_{obs} - \nu_{cal}$
3	1	2	2	0	2	2447.0805	-0.0010
4	1	4	3	1	3	2704.6996	0.0020
4	0	4	3	0	3	2731.2368	0.0047
4	1	3	3	1	2	2765.7168	-0.0009
3	2	1	2	1	1	3078.5149	-0.0096
3	2	2	2	1	2	3121.8377	0.0011
4	1	3	3	0	3	3162.6362	-0.0009
5	1	5	4	1	4	3379.9877	-0.0034
5	0	5	4	0	4	3410.3946	0.0037
5	1	4	4	1	3	3456.1135	-0.0112
4	2	2	3	1	2	3744.5776	0.0051
3	3	0	2	2	0	3796.5983	0.0097
3	3	1	2	2	1	3797.0619	-0.0175
4	2	3	3	1	3	3828.7158	0.0068
5	1	4	4	0	4	3887.5223	-0.0074
6	1	6	5	1	5	4054.7644	0.0133
6	0	6	5	0	5	4087.3031	-0.0155
6	1	5	5	1	4	4145.7860	-0.0063
5	2	3	4	1	3	4407.8576	0.0040
4	3	1	3	2	1	4479.6688	-0.0002
4	3	2	3	2	2	4482.0914	-0.0075
6	1	5	5	0	5	4622.9350	0.0039
7	0	7	6	0	6	4761.8784	0.0069
7	1	6	6	1	5	4834.5271	0.0121
6	2	4	5	1	4	5070.7253	0.0006
5	3	3	4	2	3	5168.1471	0.0000
4	4	0	3	3	0	5178.7239	-0.0087
4	4	1	3	3	1	5178.7239	-0.0087
6	2	5	5	1	5	5265.1930	-0.0001
7	1	6	6	0	6	5370.1261	-0.0015
8	1	8	7	1	7	5402.4539	-0.0165
8	1	7	7	1	6	5522.0556	-0.0019
7	2	5	6	1	5	5735.7757	-0.0001
6	3	3	5	2	3	5839.2949	0.0043
6	3	4	5	2	4	5855.7704	0.0001
5	4	1	4	3	1	5862.8305	-0.0198
5	4	2	4	3	2	5862.9419	0.0212
7	2	6	6	1	6	5994.8650	-0.0004
9	0	9	8	0	8	6104.4272	0.0072
8	1	7	7	0	7	6130.3148	0.0012
9	1	8	8	1	7	6208.1575	0.0031
8	2	6	7	1	6	6405.6241	-0.0006
7	3	4	6	2	4	6513.4329	0.0020
7	3	5	6	2	5	6545.5914	0.0022
6	4	2	5	3	2	6546.7766	0.0162
6	4	3	5	3	3	6547.0349	-0.0051
5	5	0	4	4	0	6560.6031	-0.0059
5	5	1	4	4	1	6560.6031	-0.0059
8	2	7	7	1	7	6732.1767	0.0053
9	2	7	8	1	7	7082.7099	-0.0032

Table S21 (cont.).Measured frequencies (ν_{obs}) and residuals ($\nu_{obs} - \nu_{cal}$) of the rotational transitions of isomer **4w-4** of fenchone-$(H_2O)_4$.

J'	K'_{-1}	K'_{+1}	*J"*	K''_{-1}	K''_{+1}	ν_{obs}	$\nu_{obs} - \nu_{cal}$
7	4	3	6	3	3	7230.2442	-0.0003
8	3	6	7	2	6	7238.2694	-0.0044
6	5	1	5	4	1	7244.7883	0.0057
6	5	2	5	4	2	7244.7883	0.0057
10	2	8	9	1	8	7769.1612	-0.0069
8	4	4	7	3	4	7912.9979	0.0097
8	4	5	7	3	5	7915.0384	-0.0072
7	5	2	6	4	2	7928.8835	0.0049
7	5	3	6	5	3	7928.8835	0.0049
6	6	0	5	5	0	7942.4652	-0.0031
6	6	1	5	5	1	7942.4652	-0.0031

Table S22. Measured frequencies (ν_{obs}) and residuals ($\nu_{obs} - \nu_{cal}$) of the rotational transitions of isomer **4w-7** of fenchone-$(H_2O)_4$.

J'	K'_{-1}	K'_{+1}	J''	K''_{-1}	K''_{+1}	ν_{obs}	$\nu_{obs} - \nu_{cal}$
4	0	4	3	0	3	2966.7928	-0.0088
3	3	0	2	2	0	3601.1705	-0.0006
3	3	1	2	2	1	3604.1395	-0.0067
5	1	5	4	1	4	3649.4642	-0.0025
5	0	5	4	0	4	3689.7930	0.0069
5	2	4	4	2	3	3739.0779	0.0135
5	2	3	4	2	2	3795.3006	0.0071
5	1	4	4	1	3	3814.8309	0.0011
4	3	1	3	2	1	4344.8337	0.0069
6	1	6	5	1	5	4373.1804	-0.0023
6	2	5	5	2	4	4481.1690	0.0152
5	2	3	4	1	3	4491.0678	-0.0022
6	3	4	5	3	3	4507.9646	0.0159
6	3	3	5	3	2	4517.9212	0.0014
6	1	5	5	1	4	4566.2272	-0.0073
6	2	4	5	2	3	4569.9957	-0.0125
5	3	2	4	2	2	5079.3063	-0.0014
7	0	7	6	0	6	5118.9481	0.0060
7	2	6	6	2	5	5220.2772	0.0044
6	2	4	5	1	4	5246.2465	-0.0019
7	3	5	6	3	4	5260.5432	-0.0100
7	1	6	6	1	5	5309.9909	-0.0068
7	2	5	6	2	4	5344.7616	-0.0076
6	3	3	5	2	3	5801.9510	0.0171
8	1	8	7	1	7	5814.4310	-0.0042
8	0	8	7	0	7	5830.8591	0.0007
6	3	4	5	2	4	5888.7580	-0.0041
8	2	7	7	2	6	5956.1288	0.0000
8	3	6	7	3	5	6012.2160	0.0012
8	4	4	7	4	3	6017.5874	0.0019
8	1	7	7	1	6	6044.5221	-0.0086
8	3	5	7	3	4	6052.7405	0.0047
8	2	6	7	2	5	6116.0980	-0.0028
5	5	0	4	4	0	6184.8469	0.0118
5	5	1	4	4	1	6184.8469	0.0118
6	4	2	5	3	2	6390.3614	-0.0052
6	4	3	5	3	3	6395.0800	-0.0018
9	1	9	8	1	8	6532.7039	0.0002
9	0	9	8	0	8	6543.1980	-0.0089
7	3	5	6	2	5	6668.1541	-0.0074
9	1	8	8	1	7	6769.3275	-0.0033
9	4	5	8	4	4	6777.1824	0.0027
9	3	6	8	3	5	6829.7886	-0.0064
9	2	7	8	2	6	6881.5636	0.0070
10	2	9	9	2	8	7417.6640	-0.0034
8	3	6	7	2	6	7460.0898	-0.0138
10	1	9	9	1	8	7485.7068	-0.0003
10	4	7	9	4	6	7526.7236	-0.0024
10	4	6	9	4	5	7540.6985	-0.0006
10	3	7	9	3	6	7611.4901	0.0128
10	2	8	9	2	7	7639.3824	-0.0017
9	2	7	8	1	7	7667.9178	0.0058
8	4	4	7	3	4	7868.7609	-0.0118
8	4	5	7	3	5	7901.1195	0.0114
11	0	11	10	0	10	7970.2380	0.0068

Table S23. Measured frequencies (ν_{obs}) and residuals ($\nu_{obs} - \nu_{cal}$) of the rotational transitions of isomer **4w-16** of fenchone-$(H_2O)_4$.

J'	K'_{-1}	K'_{+1}	J''	K''_{-1}	K''_{+1}	ν_{obs}	$\nu_{obs} - \nu_{cal}$
5	1	5	4	1	4	3016.7252	-0.0074
5	0	5	4	0	4	3079.8098	0.0056
5	2	4	4	2	3	3122.5785	-0.0055
5	2	3	4	2	2	3171.0308	-0.0092
5	1	4	4	1	3	3217.3052	0.0043
6	1	6	5	1	5	3614.4668	-0.0023
6	0	6	5	0	5	3673.6229	0.0026
6	2	5	5	2	4	3742.4229	0.0020
6	4	3	5	4	2	3762.6109	-0.0041
6	3	4	5	3	3	3765.3518	-0.0100
6	3	3	5	3	2	3770.9531	-0.0048
6	2	4	5	2	3	3822.6223	0.0025
6	1	5	5	1	4	3852.1010	0.0030
7	1	7	6	1	6	4209.8874	-0.0131
7	2	6	6	2	5	4359.7797	0.0166
7	3	4	6	3	3	4407.4032	0.0121
7	1	6	6	1	5	4481.2712	-0.0098
8	1	8	7	1	7	4803.1573	0.0055
8	0	8	7	0	7	4843.6174	-0.0159
8	2	7	7	2	6	4974.2790	0.0036
8	3	6	7	3	5	5024.5967	0.0076
8	1	7	7	1	6	5103.3368	-0.0015
9	1	9	8	1	8	5394.4410	-0.0207
9	0	9	8	0	8	5424.8038	0.0016
9	2	8	8	2	7	5585.6981	0.0079
9	3	7	8	3	6	5653.5402	0.0085
9	4	6	8	4	5	5654.4459	0.0066
10	1	10	9	1	9	5984.1322	-0.0024
10	0	10	9	0	9	6005.8200	0.0057
10	1	9	9	1	8	6320.7994	-0.0041
10	3	7	9	3	6	6349.7478	0.0110
10	2	8	9	2	7	6439.0422	0.0073
11	0	11	10	0	10	6587.4480	0.0176
11	2	10	10	2	9	6798.6276	-0.0012
11	1	10	10	1	9	6915.2886	0.0079
11	4	8	10	4	7	6920.3501	-0.0069
11	4	7	10	4	6	6931.0873	-0.0065
11	3	8	10	3	7	7009.6818	-0.0066
12	1	12	11	1	11	7159.8549	0.0102
12	0	12	11	0	11	7169.8454	-0.0080
12	2	11	11	2	10	7400.1378	-0.0036
12	2	10	11	2	9	7718.4912	-0.0125

8.2. Fenchone-$(H_2O)_5$

Table S24. Measured frequencies (ν_{obs}) and residuals ($\nu_{obs} - \nu_{cal}$) of the rotational transitions of isomer **5w-3** of fenchone-$(H_2O)_5$.

J'	*K'*$_{-1}$	*K'*$_{+1}$	*J''*	*K''*$_{-1}$	*K''*$_{+1}$	ν_{obs}	$\nu_{obs} - \nu_{cal}$
4	1	4	3	1	3	2234.5740	0.0038
4	0	4	3	0	3	2268.9969	-0.0039
4	2	3	3	2	2	2278.3031	0.0009
4	2	2	3	2	1	2288.3999	-0.0001
4	1	3	3	1	2	2319.5301	0.0031
5	1	5	4	1	4	2791.4921	0.0020
5	0	5	4	0	4	2829.0723	-0.0013
5	2	4	4	2	3	2846.5955	0.0003
5	4	2	4	4	1	2851.1567	0.0100
5	4	1	4	4	0	2851.1567	0.0100
5	3	3	4	3	2	2852.0849	0.0092
5	3	2	4	3	1	2852.7164	-0.0036
5	2	3	4	2	2	2866.3858	0.0001
5	1	4	4	1	3	2897.2776	0.0058
6	1	6	5	1	5	3347.4172	0.0001
6	0	6	5	0	5	3385.1564	0.0002
6	2	5	5	2	4	3414.0382	-0.0003
6	5	1	5	5	0	3421.1952	-0.0032
6	5	2	5	5	1	3421.1952	-0.0032
6	4	3	5	4	2	3422.1207	-0.0024
6	4	2	5	4	1	3422.1207	-0.0024
6	3	3	5	3	2	3425.1487	-0.0012
6	2	4	5	2	3	3447.4924	-0.0009
6	1	5	5	1	4	3473.4173	-0.0015
7	1	7	6	1	6	3902.2935	-0.0008
7	0	7	6	0	6	3937.4969	0.0000
7	2	6	6	2	5	3980.4711	-0.0012
7	6	1	6	6	0	3991.2725	0.0032
7	6	2	6	6	1	3991.2725	0.0032
7	5	2	6	5	1	3992.0374	-0.0010
7	5	3	6	5	2	3992.0374	-0.0010
7	3	5	6	3	4	3995.0828	-0.0005
7	3	4	6	3	3	3998.8881	-0.0013
7	2	5	6	2	4	4031.2863	-0.0011
7	1	6	6	1	5	4047.4796	-0.0008
8	1	8	7	1	7	4456.1221	0.0001
8	0	8	7	0	7	4486.8678	0.0009
8	2	7	7	2	6	4545.7459	-0.0015
8	7	1	7	7	0	4561.3550	0.0076
8	7	2	7	7	1	4561.3550	0.0076
8	5	3	7	5	2	4563.1741	-0.0008
8	5	4	7	5	3	4563.1741	-0.0008
8	4	5	7	4	4	4565.2526	0.0014
8	4	4	7	4	3	4565.5015	-0.0011
8	3	6	7	3	5	4566.8711	0.0007
8	3	5	7	3	4	4574.3661	-0.0019
8	2	6	7	2	5	4616.8196	0.0251
8	1	7	7	1	6	4618.8967	0.0005
9	1	9	8	1	8	5008.9569	0.0046
9	0	9	8	0	8	5034.3111	0.0024
9	2	8	8	2	7	5109.7302	-0.0010
9	6	3	8	6	2	5133.0127	-0.0010
9	6	4	8	6	3	5133.0127	-0.0010
9	5	4	8	5	3	5134.6508	-0.0006
9	5	5	8	5	4	5134.6508	-0.0006
9	4	6	8	4	5	5137.5006	-0.0012

Table S24 (cont.). Measured frequencies (ν_{obs}) and residuals ($\nu_{obs} - \nu_{cal}$) of the rotational transitions of isomer **5w-3** of fenchone-$(H_2O)_5$.

J'	*K'*$_{-1}$	*K'*$_{+1}$	*J''*	*K''*$_{-1}$	*K''*$_{+1}$	ν_{obs}	$\nu_{obs} - \nu_{cal}$
9	4	5	8	4	4	5138.0997	-0.0015
9	3	7	8	3	6	5138.6211	-0.0014
9	3	6	8	3	5	5152.0583	-0.0015
9	1	8	8	1	7	5187.0587	-0.0012
9	2	7	8	2	6	5202.7451	-0.0007
10	1	10	9	1	9	5560.8745	-0.0022
10	0	10	9	0	9	5580.8194	0.0104
10	2	9	9	2	8	5672.3150	0.0006
10	7	3	9	7	2	5702.9316	-0.0032
10	7	3	9	7	3	5702.9316	-0.0032
10	6	4	9	6	3	5704.2624	0.0011
10	6	5	9	6	4	5704.2624	0.0011
10	5	5	9	5	4	5706.5138	0.0014
10	5	6	9	5	5	5706.5138	0.0014
10	3	8	9	3	7	5710.1101	-0.0104
10	4	7	9	4	6	5710.2314	0.0112
10	4	6	9	4	5	5711.5047	-0.0025
10	3	7	9	3	6	5732.4129	-0.0013
10	1	9	9	1	8	5751.3851	-0.0011
10	2	8	9	2	7	5787.8916	-0.0021
11	0	11	10	0	10	6127.0835	0.0001
11	1	11	10	1	11	6163.2755	-0.0051
11	2	10	10	2	9	6233.4171	-0.0010
11	8	3	10	8	2	6272.8840	-0.0154
11	8	4	10	8	3	6272.8840	-0.0154
11	7	4	10	7	3	6274.0272	-0.0042
11	7	4	10	7	4	6274.0272	-0.0042
11	6	5	10	6	4	6275.7982	-0.0006
11	6	6	10	6	5	6275.7982	-0.0006
11	5	6	10	5	5	6278.7960	-0.0082
11	5	7	10	5	6	6278.7960	-0.0082
11	3	9	10	3	8	6281.1202	-0.0014
11	4	8	10	4	7	6283.3804	-0.0007
11	4	7	10	4	6	6285.9204	-0.0024
11	1	10	10	1	9	6311.4296	-0.0011
11	3	8	10	3	7	6315.7332	-0.0013
11	2	9	10	2	8	6371.2138	-0.0009
12	1	12	11	1	11	6662.4852	-0.0012
12	0	12	11	0	11	6673.5370	0.0006
12	2	11	11	2	10	6792.9962	-0.0037
12	8	4	11	8	3	6843.8956	0.0076
12	8	5	11	8	4	6843.8956	0.0076
12	7	5	11	7	4	6845.3599	0.0016
12	7	6	11	7	5	6845.3599	0.0016
12	6	6	11	6	5	6847.6546	-0.0020
12	6	7	11	6	6	6847.6546	-0.0020
12	3	10	11	3	9	6851.3698	-0.0067
12	5	8	11	5	7	6851.4852	0.0074
12	5	7	11	5	6	6851.6713	-0.0008
12	4	9	11	4	8	6856.9200	-0.0017
12	4	8	11	4	7	6861.6062	-0.0016
12	1	11	11	1	10	6867.0447	-0.0014
12	3	9	11	3	8	6902.0172	-0.0005
12	2	10	11	2	9	6951.9364	-0.0019
13	1	13	12	1	12	7212.4264	0.0009
13	0	13	12	0	12	7220.3341	0.0011
13	2	12	12	2	11	7351.0602	0.0012

Table S24 (cont.). Measured frequencies (ν_{obs}) and residuals ($\nu_{obs} - \nu_{cal}$) of the rotational transitions of isomer **5w-3** of fenchone-$(H_2O)_5$.

J'	K'_{-1}	K'_{+1}	*J"*	$K"_{-1}$	$K"_{+1}$	ν_{obs}	$\nu_{obs} - \nu_{cal}$
13	8	5	12	8	4	7415.0672	0.0008
13	8	6	12	8	5	7415.0672	0.0008
13	7	6	12	7	5	7416.9356	-0.0017
13	7	7	12	7	6	7416.9356	-0.0017
13	1	12	12	1	11	7418.5140	-0.0022
13	6	7	12	6	6	7419.8643	-0.0016
13	6	8	12	6	7	7419.8643	-0.0016
13	3	11	12	3	10	7420.6422	-0.0021
13	5	9	12	5	8	7424.6710	-0.0007
13	5	8	12	5	7	7425.0796	-0.0012
13	4	10	12	4	9	7430.7285	-0.0094
13	4	9	12	4	8	7438.8849	-0.0006
13	3	10	12	3	9	7490.8124	-0.0039
13	2	11	12	2	10	7529.4771	0.0091
14	1	14	13	1	13	7761.9453	0.0003
14	0	14	13	0	13	7767.4937	-0.0022
14	2	13	13	2	12	7907.6357	-0.0017
14	1	13	13	1	12	7966.5767	0.0001
14	8	6	13	8	5	7986.4633	0.0126
14	8	7	13	8	6	7986.4633	0.0126
14	3	12	13	3	11	7988.7313	0.0262
14	6	8	13	6	7	7992.4620	0.0029
14	6	9	13	6	8	7992.4620	0.0029
14	5	10	13	5	9	7998.3427	-0.0165
14	5	9	13	5	8	7999.1724	0.0041

Table S25. Measured frequencies (ν_{obs}) and residuals ($\nu_{obs} - \nu_{cal}$) of the rotational transitions of isomer **5w-1** of fenchone-$(H_2O)_5$.

J'	K'_{-1}	K'_{+1}	J''	K''_{-1}	K''_{+1}	ν_{obs}	$\nu_{obs} - \nu_{cal}$
4	1	4	3	1	3	2289.8648	0.0033
4	0	4	3	0	3	2325.7293	-0.0043
4	2	3	3	2	2	2339.1313	-0.0007
4	2	2	3	2	1	2353.6900	-0.0007
4	1	3	3	1	2	2384.7497	-0.0017
5	1	5	4	1	4	2859.9062	0.0009
5	0	5	4	0	4	2897.1718	-0.0086
5	2	4	4	2	3	2922.0527	0.0007
5	4	2	4	4	1	2928.6802	0.0164
5	4	1	4	4	0	2928.6802	0.0012
5	3	3	4	3	2	2929.8931	-0.0006
5	3	2	4	3	1	2931.1152	0.0046
5	2	3	4	2	2	2950.2096	-0.0115
5	1	4	4	1	3	2977.7356	-0.0014
6	1	6	5	1	5	3428.6220	0.0025
6	0	6	5	0	5	3463.6928	-0.0006
6	2	5	5	2	4	3503.7446	0.0011
6	5	1	5	5	0	3514.1391	-0.0057
6	5	2	5	5	1	3514.1391	-0.0057
6	4	2	5	4	1	3515.5347	0.0019
6	3	4	5	3	3	3517.0793	0.0009
6	3	3	5	3	2	3520.2906	-0.0009
6	2	4	5	2	3	3550.4179	0.0028
6	1	5	5	1	4	3568.2638	0.0006
7	1	7	6	1	6	3995.9883	0.0020
7	0	7	6	0	6	4026.2309	0.0001
7	2	6	6	2	5	4083.9854	0.0006
7	6	1	6	6	0	4099.6466	0.0018
7	6	2	6	6	1	4099.6466	0.0018
7	5	2	6	5	1	4100.7753	0.0014
7	5	3	6	5	2	4100.7753	0.0014
7	4	4	6	4	3	4102.8069	-0.0083
7	4	3	6	4	2	4103.0430	0.0021
7	3	5	6	3	4	4104.4870	0.0007
7	3	4	6	3	3	4111.5959	-0.0002
7	2	5	6	2	4	4153.0284	0.0011
7	1	6	6	1	5	4155.5396	0.0004
8	1	8	7	1	7	4562.0740	-0.0016
8	0	8	7	0	7	4586.2756	-0.0001
8	2	7	7	2	6	4662.5800	-0.0003
8	7	1	7	7	0	4685.1560	0.0025
8	7	2	7	7	1	4685.1560	0.0025
8	6	2	7	6	1	4686.1508	-0.0030
8	6	3	7	6	2	4686.1508	-0.0030
8	5	3	7	5	2	4687.8385	0.0000
8	5	4	7	5	3	4687.8385	0.0000
8	4	5	7	4	4	4690.7615	0.0011
8	4	4	7	4	3	4691.3754	-0.0010
8	3	6	7	3	5	4691.8610	0.0002
8	3	5	7	3	4	4705.7085	-0.0003
8	1	7	7	1	6	4738.6862	-0.0025
8	2	6	7	2	5	4756.2527	0.0022
9	1	9	8	1	8	5127.0239	0.0005
9	0	9	8	0	8	5145.2393	-0.0002
9	2	8	8	2	7	5239.3717	-0.0006
9	7	2	8	7	1	5271.5719	-0.0087
9	7	3	8	7	2	5271.5719	-0.0087

Table S25 (cont.).Measured frequencies (ν_{obs}) and residuals ($\nu_{obs} - \nu_{cal}$) of the rotational transitions of isomer **5w-1** of fenchone-($H_2O)_5$.

J'	*K'*$_{-1}$	*K'*$_{+1}$	*J"*	*K"*$_{-1}$	*K"*$_{+1}$	ν_{obs}	$\nu_{obs} - \nu_{cal}$
9	6	3	8	6	2	5273.0034	0.0012
9	6	4	8	6	3	5273.0034	0.0012
9	5	4	8	5	3	5275.4064	0.0027
9	5	5	8	5	4	5275.4064	0.0027
9	3	7	8	3	6	5278.8836	0.0035
9	4	6	8	4	5	5279.3090	0.0020
9	4	5	8	4	4	5280.7723	0.0033
9	3	6	8	3	5	5303.2727	-0.0010
9	1	8	8	1	7	5316.8706	0.0001
9	2	7	8	2	6	5358.2921	0.0000
10	1	10	9	1	9	5691.0054	0.0001
10	0	10	9	0	9	5704.0754	-0.0013
10	2	9	9	2	8	5814.2563	0.0010
10	8	2	9	8	1	5857.0274	-0.0019
10	8	3	9	8	2	5857.0274	-0.0019
10	7	3	9	7	2	5858.2838	-0.0010
10	7	4	9	7	3	5858.2838	-0.0010
10	6	4	9	6	3	5860.2336	-0.0003
10	6	5	9	6	4	5860.2336	-0.0003
10	5	6	9	5	5	5863.4691	-0.0135
10	5	5	9	5	4	5863.6028	0.0104
10	3	8	9	3	7	5865.1895	0.0021
10	4	7	9	4	6	5868.4101	0.0009
10	4	6	9	4	5	5871.5270	-0.0002
10	3	7	9	3	6	5904.6610	-0.0004
10	2	8	9	2	7	5957.7357	-0.0010
11	1	11	10	1	10	6254.2087	-0.0003
11	0	11	10	0	10	6263.2551	0.0034
11	2	10	10	2	9	6387.1885	0.0016
11	8	3	10	8	2	6443.6338	0.0044
11	8	4	10	8	3	6443.6338	0.0044
11	7	4	10	7	3	6445.2964	-0.0014
11	7	5	10	7	4	6445.2964	-0.0014
11	6	5	10	6	4	6447.8954	0.0017
11	6	6	10	6	5	6447.8954	0.0017
11	5	7	10	5	6	6452.1772	0.0014
11	5	6	10	5	5	6452.4385	-0.0093
11	1	10	10	1	9	6456.5789	-0.0002
11	4	8	10	4	7	6457.9565	-0.0007
11	4	7	10	4	6	6464.0570	-0.0007
11	3	8	10	3	7	6509.6709	0.0006
11	2	9	10	2	8	6553.5580	-0.0002
12	1	12	11	1	11	6816.8118	-0.0009
12	0	12	11	0	11	6822.8932	-0.0029
12	1	11	11	1	10	7018.8070	-0.0019
12	7	5	11	7	4	7032.6617	0.0103
12	7	6	11	7	5	7032.6617	0.0103
12	3	10	11	3	9	7034.2363	-0.0050
12	6	6	11	6	5	7036.0260	-0.0022
12	6	7	11	6	6	7036.0260	-0.0022
12	5	8	11	5	7	7041.4945	-0.0025
12	5	7	11	5	6	7042.1070	-0.0044
12	4	9	11	4	8	7047.7735	0.0030
12	4	8	11	4	7	7058.8750	0.0030
12	3	9	11	3	8	7117.3293	0.0029
12	2	10	11	2	9	7144.9625	0.0063
13	0	13	12	0	12	7382.9779	0.0012

Table S25 (cont.). Measured frequencies (ν_{obs}) and residuals ($\nu_{obs} - \nu_{cal}$) of the rotational transitions of isomer **5w-1** of fenchone-$(H_2O)_5$.

J'	*K'*$_{-1}$	*K'*$_{+1}$	*J"*	*K"*$_{-1}$	*K"*$_{+1}$	ν_{obs}	$\nu_{obs} - \nu_{cal}$
13	2	12	12	2	11	7527.3794	0.0025
13	3	11	12	3	10	7616.3534	-0.0009
13	7	6	12	7	5	7620.3793	0.0006
13	7	7	12	7	6	7620.3793	0.0006
13	5	8	12	5	7	7632.7387	-0.0052
13	4	9	12	4	8	7656.5549	-0.0022
13	3	10	12	3	9	7725.9728	-0.0013
13	2	11	12	2	10	7731.2156	0.0012
14	1	14	13	1	13	7940.8131	0.0016
14	0	14	13	0	13	7943.4030	-0.0008

Table S26. Measured frequencies (ν_{obs}) and residuals ($\nu_{obs} - \nu_{cal}$) of the rotational transitions of isomer **5w-7** of fenchone-$(H_2O)_5$.

J'	*K'*$_{-1}$	*K'*$_{+1}$	*J"*	*K"*$_{-1}$	*K"*$_{+1}$	ν_{obs}	$\nu_{obs} - \nu_{cal}$
4	0	4	3	0	3	2213.5141	-0.0060
4	1	3	3	1	2	2288.3999	0.0099
5	1	5	4	1	4	2705.6470	-0.0037
5	0	5	4	0	4	2754.6281	0.0058
5	2	4	4	2	3	2785.0591	-0.0054
5	3	2	4	3	1	2796.0613	0.0083
5	2	3	4	2	2	2819.5131	-0.0047
5	1	4	4	1	3	2856.6202	0.0041
6	1	6	5	1	5	3242.7635	0.0032
6	0	6	5	0	5	3289.4875	-0.0037
6	2	5	5	2	4	3338.7677	0.0002
6	5	1	5	5	0	3351.4482	0.0021
6	5	2	5	5	1	3351.4482	0.0021
6	3	4	5	3	3	3355.0887	0.0009
6	3	3	5	3	2	3358.8121	0.0015
6	2	4	5	2	3	3396.1024	-0.0050
6	1	5	5	1	4	3421.8865	0.0017
7	1	7	6	1	6	3778.1840	-0.0021
7	0	7	6	0	6	3819.1597	-0.0006
7	2	6	6	2	5	3890.7023	0.0007
7	4	4	6	4	3	3913.6493	0.0016
7	4	3	6	4	2	3913.8863	-0.0098
7	3	5	6	3	4	3915.8516	0.0037
7	3	4	6	3	3	3924.0996	0.0003
7	2	5	6	2	4	3976.0105	0.0006
7	1	6	6	1	5	3983.2529	0.0002
8	1	8	7	1	7	4311.9961	-0.0012
8	0	8	7	0	7	4345.3829	-0.0020
8	2	7	7	2	6	4440.6229	0.0004
8	5	3	7	5	2	4471.4119	0.0025
8	5	4	7	5	3	4471.4119	0.0025
8	4	5	7	4	4	4474.9848	0.0009
8	4	4	7	4	3	4475.6604	-0.0018
8	3	6	7	3	5	4476.6283	0.0001
8	3	5	7	3	4	4492.7421	-0.0014
8	1	7	7	1	6	4539.6649	0.0018
8	2	6	7	2	5	4557.0853	0.0013
9	1	9	8	1	8	4844.3473	0.0005
9	0	9	8	0	8	4869.9485	-0.0024
9	2	8	8	2	7	4988.3264	-0.0026

Table S26 (cont.). Measured frequencies (ν_{obs}) and residuals ($\nu_{obs} - \nu_{cal}$) of the rotational transitions of isomer **5w-7** of fenchone-$(H_2O)_5$.

J'	*K'*$_{-1}$	*K'*$_{+1}$	*J"*	*K"*$_{-1}$	*K"*$_{+1}$	ν_{obs}	$\nu_{obs} - \nu_{cal}$
9	3	7	8	3	6	5037.0504	0.0008
9	1	8	8	1	7	5090.0682	0.0018
9	2	7	8	2	6	5137.0970	0.0018
10	1	10	9	1	9	5375.4417	0.0007
10	0	10	9	0	9	5394.1538	0.0004
10	2	9	9	2	8	5533.6793	0.0013
10	7	3	9	7	2	5587.4571	-0.0069
10	7	3	9	7	3	5587.4571	-0.0069
10	6	4	9	6	3	5589.8137	0.0004
10	6	5	9	6	4	5589.8137	0.0004
10	3	8	9	3	7	5596.6836	-0.0005
10	4	7	9	4	6	5599.8750	0.0003
10	4	6	9	4	5	5603.3255	0.0080
10	1	9	9	1	8	5633.6620	-0.0003
10	3	7	9	3	6	5643.0798	-0.0050
10	2	8	9	2	7	5714.2091	-0.0008
11	1	11	10	1	10	5905.5090	0.0018
11	0	11	10	0	10	5918.6854	0.0016
11	2	10	10	2	9	6076.6065	0.0074
11	7	4	10	7	3	6147.6571	0.0037
11	7	4	10	7	4	6147.6571	0.0037
11	6	5	10	6	4	6150.7857	-0.0010
11	6	6	10	6	5	6150.7857	-0.0010
11	3	9	10	3	8	6155.0868	-0.0029
11	5	7	10	5	6	6155.9995	0.0079
11	5	6	10	5	5	6156.2650	-0.0116
11	4	7	10	4	6	6170.0261	0.0006
11	1	10	10	1	9	6170.2333	-0.0009
11	3	8	10	3	7	6225.2844	-0.0005
11	2	9	10	2	8	6287.0811	-0.0027
12	1	12	11	1	11	6434.7711	0.0036
12	0	12	11	0	11	6443.7833	-0.0016
12	2	11	11	2	10	6617.1057	0.0021
12	3	10	11	3	9	6711.8409	-0.0034
12	6	6	11	6	5	6712.3391	0.0022
12	6	7	11	6	6	6712.3391	0.0022
12	5	7	11	5	6	6719.6509	0.0018
12	4	9	11	4	8	6727.0677	-0.0072
12	4	8	11	4	7	6739.4004	0.0008
12	2	10	11	2	9	6854.6934	0.0003
13	1	13	12	1	12	6963.4238	0.0042
13	0	13	12	0	12	6969.4587	0.0030
13	2	12	12	2	11	7155.2862	-0.0007
13	1	12	12	1	11	7225.7129	0.0017
13	5	9	12	5	8	7282.8160	0.0007
13	5	8	12	5	7	7284.1587	-0.0074
13	4	10	12	4	9	7290.9932	-0.0021
13	4	9	12	4	8	7312.1304	-0.0055
13	3	10	12	3	9	7399.0738	0.0008
13	2	11	12	2	10	7416.1476	0.0004
14	1	14	13	1	13	7491.6208	-0.0071
14	2	13	13	2	12	7691.3239	0.0026
14	1	13	13	1	12	7748.0214	-0.0029
14	3	12	13	3	11	7818.9613	-0.0016
14	4	11	13	4	10	7854.6785	-0.0001
14	4	10	13	4	9	7888.9667	0.0135

8.3. Fenchone-$(H_2O)_6$

Table S27. Measured frequencies (ν_{obs}) and residuals ($\nu_{obs} - \nu_{cal}$) of the rotational transitions of isomer **6w-1** of fenchone-$(H_2O)_6$.

J'	*K'*$_{-1}$	*K'*$_{+1}$	*J"*	*K"*$_{-1}$	*K"*$_{+1}$	ν_{obs}	$\nu_{obs} - \nu_{cal}$
5	1	5	4	1	4	2408.8808	0.0007
5	0	5	4	0	4	2445.4279	0.0011
5	2	4	4	2	3	2485.1085	-0.0023
5	3	3	4	3	2	2497.9323	-0.0040
5	3	2	4	3	1	2500.7514	-0.0046
5	2	3	4	2	2	2530.3090	0.0019
5	1	4	4	1	3	2550.4033	0.0012
6	1	6	5	1	5	2885.6408	-0.0019
6	0	6	5	0	5	2916.4032	-0.0160
6	2	5	5	2	4	2977.5979	0.0049
6	4	3	5	4	2	2997.2470	0.0033
6	3	4	5	3	3	2999.0730	-0.0018
6	3	3	5	3	2	3006.4478	-0.0004
6	2	4	5	2	3	3049.7584	0.0017
6	1	5	5	1	4	3051.4757	0.0002
7	1	7	6	1	6	3360.5810	0.0005
7	0	7	6	0	6	3383.9716	0.0041
7	2	6	6	2	5	3467.6902	-0.0132
7	6	1	6	6	0	3493.9688	0.0126
7	6	2	6	6	1	3493.9688	0.0126
7	4	4	6	4	3	3499.1180	0.0012
7	4	3	6	4	2	3499.8485	0.0066
7	3	5	6	3	4	3500.1498	-0.0006
7	3	4	6	3	3	3516.1988	0.0039
7	1	6	6	1	5	3546.5985	0.0015
7	2	5	6	2	4	3569.9407	-0.0002
8	1	8	7	1	7	3833.9408	0.0045
8	0	8	7	0	7	3850.3345	0.0011
8	2	7	7	2	6	3955.1878	0.0004
8	6	2	7	6	1	3994.5353	0.0121
8	6	3	7	6	2	3994.5353	0.0121
8	3	6	7	3	5	4000.6074	-0.0021
8	4	5	7	4	4	4001.8506	0.0003
8	4	4	7	4	3	4003.8135	-0.0015
8	1	7	7	1	6	4034.4056	0.0007
8	2	6	7	2	5	4087.9459	0.0019
9	1	9	8	1	8	4306.0195	0.0011
9	0	9	8	0	8	4316.8482	0.0013
9	7	2	8	7	1	4493.2902	-0.0008
9	7	3	8	7	2	4493.2902	-0.0008
9	6	3	8	6	2	4495.6660	-0.0003
9	6	4	8	6	3	4495.6660	-0.0003
9	5	5	8	5	4	4499.6230	-0.0012
9	3	7	8	3	6	4499.8423	0.0002
9	4	6	8	4	5	4505.3301	-0.0061
9	4	5	8	4	4	4509.9467	-0.0013
9	1	8	8	1	7	4514.1944	0.0011
9	3	6	8	3	5	4551.2972	0.0019
10	1	10	9	1	9	4777.1432	0.0033
10	0	10	9	0	9	4783.9859	-0.0002
10	2	9	9	2	8	4921.8277	0.0037

Table S27 (cont.). Measured frequencies (ν_{obs}) and residuals ($\nu_{obs} - \nu_{cal}$) of the rotational transitions of isomer **6w-1** of fenchone-$(H_2O)_6$.

J'	*K'*-1	*K'*+1	*J''*	*K''*-1	*K''*+1	ν_{obs}	$\nu_{obs} - \nu_{cal}$
10	7	3	9	7	2	4994.1991	0.0040
10	7	4	9	7	3	4994.1991	0.0040
10	3	8	9	3	7	4997.2516	-0.0004
10	6	4	9	6	3	4997.4582	-0.0041
10	6	5	9	6	4	4997.4582	-0.0041
10	5	6	9	5	5	5002.8096	-0.0013
10	5	5	9	5	4	5003.2913	-0.0054
10	4	7	9	4	6	5009.3379	0.0054
10	4	6	9	4	5	5019.0060	-0.0034
10	3	7	9	3	6	5076.0407	-0.0004
10	2	8	9	2	7	5109.4425	-0.0015
11	1	11	10	1	10	5247.5746	-0.0032
11	0	11	10	0	10	5251.7713	0.0014
11	2	10	10	2	9	5401.0904	0.0005
11	1	10	10	1	9	5453.3851	-0.0004
11	3	9	10	3	8	5492.3140	-0.0001
11	8	3	10	8	2	5492.8412	0.0002
11	8	4	10	8	3	5492.8412	0.0002
11	7	4	10	7	3	5495.6266	0.0020
11	7	5	10	7	4	5495.6266	0.0020
11	6	5	10	6	4	5499.9775	-0.0139
11	6	6	10	6	5	5499.9775	-0.0139
11	5	7	10	5	6	5506.9274	0.0090
11	5	6	10	5	5	5508.1045	-0.0075
11	4	8	10	4	7	5513.4575	0.0015
11	4	7	10	4	6	5531.9493	0.0010
11	3	8	10	3	7	5602.8398	-0.0003
11	2	9	10	2	8	5610.2002	0.0028
12	1	12	11	1	11	5717.5527	0.0003
12	0	12	11	0	11	5720.0584	0.0001
12	2	11	11	2	10	5877.9519	0.0012
12	1	11	11	1	10	5917.3936	0.0010
12	3	10	11	3	9	5984.6175	0.0023
12	8	4	11	8	3	5994.0114	-0.0017
12	8	5	11	8	4	5994.0114	-0.0017
12	7	5	11	7	4	5997.6410	0.0048
12	7	6	11	7	5	5997.6410	0.0048
12	6	7	11	6	6	6003.2656	-0.0162
12	6	6	11	6	5	6003.3970	0.0005
12	5	8	11	5	7	6011.9276	0.0036
12	5	7	11	5	6	6014.5911	0.0019
12	4	9	11	4	8	6017.1964	-0.0018
12	4	8	11	4	7	6049.7345	0.0021
12	2	10	11	2	9	6102.7616	-0.0026
12	3	9	11	3	8	6128.4866	-0.0082
13	0	13	12	0	12	6188.6972	0.0009
13	2	12	12	2	11	6352.7483	-0.0035
13	1	12	12	1	11	6380.8002	-0.0030
13	3	11	12	3	10	6473.8816	0.0008
13	8	5	12	8	4	6495.6647	-0.0009
13	8	6	12	8	5	6495.6647	-0.0009
13	7	6	12	7	5	6500.2896	0.0007
13	7	7	12	7	6	6500.2896	0.0007
13	6	8	12	6	7	6507.4491	-0.0042
13	6	7	12	6	6	6507.7465	0.0025
13	5	9	12	5	8	6517.7195	-0.0027
13	4	10	12	4	9	6519.9670	0.0024
13	5	8	12	5	7	6523.2118	-0.0025

Table S27 (cont.). Measured frequencies (ν_{obs}) and residuals ($\nu_{obs} - \nu_{cal}$) of the rotational transitions of isomer **6w-1** of fenchone-$(H_2O)_6$.

J'	*K'*$_{-1}$	*K'*$_{+1}$	*J"*	*K"*$_{-1}$	*K"*$_{+1}$	ν_{obs}	$\nu_{obs} - \nu_{cal}$
13	4	9	12	4	8	6572.9657	0.0044
13	2	11	12	2	10	6586.3909	-0.0035
13	3	10	12	3	9	6650.0482	-0.0004
14	1	14	13	1	13	6656.7070	-0.0040
14	0	14	13	0	13	6657.5563	-0.0044
14	2	13	13	2	12	6825.8922	0.0096
14	1	13	13	1	12	6844.9153	-0.0016
14	3	12	13	3	11	6959.9973	0.0071
14	8	6	13	8	5	6997.8450	0.0029
14	8	7	13	8	6	6997.8450	0.0029
14	4	11	13	4	10	7021.1381	0.0070
14	2	12	13	2	11	7061.2082	-0.0026
14	4	10	13	4	9	7101.2935	0.0024
14	3	11	13	3	10	7165.3123	0.0019
15	2	14	14	2	13	7297.7272	-0.0007
15	3	13	14	3	12	7442.9814	0.0013
15	6	10	14	6	9	7518.5448	-0.0033
15	2	13	14	2	12	7528.6334	0.0031
15	3	12	14	3	11	7672.7489	-0.0027

Table S28. Measured frequencies (ν_{obs}) and residuals ($\nu_{obs} - \nu_{cal}$) of the rotational transitions of isomer **6w-2** of fenchone-$(H_2O)_6$.

J'	*K'*$_{-1}$	*K'*$_{+1}$	*J"*	*K"*$_{-1}$	*K"*$_{+1}$	ν_{obs}	$\nu_{obs} - \nu_{cal}$
5	1	5	4	1	4	2355.5056	-0.0054
5	0	5	4	0	4	2396.9175	-0.0116
5	2	4	4	2	3	2455.8173	-0.0092
5	1	4	4	1	3	2539.1647	-0.0026
6	1	6	5	1	5	2819.3150	-0.0016
6	0	6	5	0	5	2851.8118	0.0017
6	2	5	5	2	4	2940.0204	0.0004
6	3	4	5	3	3	2972.2814	-0.0028
6	3	3	5	3	2	2985.5457	0.0002
6	1	5	5	1	4	3032.5722	0.0010
6	2	4	5	2	3	3045.0217	-0.0003
7	1	7	6	1	6	3280.6837	0.0029
7	0	7	6	0	6	3303.5638	0.0005
7	2	6	6	2	5	3420.6343	0.0029
7	3	5	6	3	4	3468.8145	-0.0046
7	4	4	6	4	3	3468.8145	0.0013
7	4	3	6	4	2	3470.3623	-0.0032
7	3	4	6	3	3	3497.2713	0.0073
7	1	6	6	1	5	3516.4740	-0.0004
7	2	5	6	2	4	3565.6764	0.0019
8	1	8	7	1	7	3740.0504	0.0016
8	0	8	7	0	7	3754.9254	0.0033
8	2	7	7	2	6	3897.3545	-0.0001
8	6	2	7	6	1	3957.6852	0.0032
8	6	3	7	6	2	3957.6852	0.0032
8	3	6	7	3	5	3963.9569	-0.0065
8	4	5	7	4	4	3968.5131	0.0024
8	4	4	7	4	3	3972.6951	0.0047
8	1	7	7	1	6	3989.2280	-0.0012
8	3	5	7	3	4	4016.6817	0.0135

Table S28 (cont.). Measured frequencies (ν_{obs}) and residuals ($\nu_{obs} - \nu_{cal}$) of the rotational transitions of isomer **6w-2** of fenchone-$(H_2O)_6$.

J'	*K'*$_{-1}$	*K'*$_{+1}$	*J"*	*K"*$_{-1}$	*K"*$_{+1}$	ν_{obs}	$\nu_{obs} - \nu_{cal}$
8	2	6	7	2	5	4081.2791	-0.0024
9	1	9	8	1	8	4197.9049	-0.0077
9	0	9	8	0	8	4207.0438	0.0002
9	1	8	8	1	7	4450.9352	-0.0011
9	3	7	8	3	6	4456.7734	-0.0022
9	5	5	8	5	4	4461.2820	0.0016
9	5	4	8	5	3	4461.7132	-0.0112
9	4	6	8	4	5	4469.0726	-0.0010
9	4	5	8	4	4	4478.7940	0.0023
10	1	10	9	1	9	4654.7247	0.0041
10	0	10	9	0	9	4660.1097	0.0065
10	2	9	9	2	8	4838.9809	0.0006
10	3	8	9	3	7	4946.4063	0.0007
10	7	3	9	7	2	4948.7322	-0.0014
10	7	4	9	7	3	4948.7322	-0.0014
10	6	4	9	6	3	4953.7828	-0.0067
10	6	5	9	6	4	4953.7828	-0.0067
10	5	6	9	5	5	4961.8926	0.0106
10	5	5	9	5	4	4963.1053	0.0022
10	4	7	9	4	6	4969.9676	-0.0015
10	4	6	9	4	5	4990.0716	0.0049
10	3	7	9	3	6	5073.3439	-0.0011
10	2	8	9	2	7	5087.5047	-0.0015
11	1	11	10	1	10	5110.8301	-0.0017
11	0	11	10	0	10	5113.9237	0.0118
11	2	10	10	2	9	5304.3848	0.0046
11	1	10	10	1	9	5352.6173	-0.0059
11	3	9	10	3	8	5432.1793	0.0017
11	4	8	10	4	7	5470.4488	0.0051
12	1	12	11	1	11	5566.4998	-0.0062
12	0	12	11	0	11	5568.2363	0.0068
11	2	9	10	2	8	5574.3938	0.0055
11	3	8	10	3	7	5602.6633	-0.0071
12	2	11	11	2	10	5766.8155	0.0004
12	1	11	11	1	10	5800.1402	-0.0009
12	3	10	11	3	9	5913.6415	-0.0020
12	5	8	11	5	7	5966.8352	0.0116
12	4	9	11	4	8	5969.5831	-0.0055
12	5	7	11	5	6	5973.3972	-0.0101
13	0	13	12	0	12	6022.8581	-0.0072
12	4	8	11	4	7	6033.6857	0.0005
12	2	10	11	2	9	6048.6396	0.0071
12	3	9	11	3	8	6126.4246	0.0011
13	3	11	12	3	10	6390.6065	-0.0054
13	6	7	12	6	6	6457.3734	-0.0042
13	4	10	12	4	9	6466.4342	-0.0040
13	5	9	12	5	8	6470.6843	-0.0108
14	0	14	13	0	13	6477.6916	0.0006
13	5	8	12	5	7	6484.0709	-0.0021
13	2	11	12	2	10	6510.4105	0.0030
13	4	9	12	4	8	6566.5353	0.0044
14	2	13	13	2	12	6685.1956	0.0061
14	1	13	13	1	12	6698.8307	-0.0079
15	4	12	14	4	11	7449.7574	0.0077

8.4. Fenchone-$(H_2O)_7$

Table S29. Measured frequencies (ν_{obs}) and residuals ($\nu_{obs} - \nu_{cal}$) of the rotational transitions of isomer **7w-1** of fenchone-$(H_2O)_7$.

J'	*K'*$_{-1}$	*K'*$_{+1}$	*J''*	*K''*$_{-1}$	*K''*$_{+1}$	ν_{obs}	$\nu_{obs} - \nu_{cal}$
6	1	6	5	1	5	2419.5668	0.0045
6	0	6	5	0	5	2452.3813	-0.0005
6	2	5	5	2	4	2478.0288	0.0070
6	5	2	5	5	1	2484.4070	0.0102
6	5	1	5	5	0	2484.4070	0.0102
6	3	4	5	3	3	2486.3958	0.0180
6	2	4	5	2	3	2507.7306	0.0034
6	1	5	5	1	4	2530.0440	0.0074
7	1	7	6	1	6	2820.1421	-0.0020
7	0	7	6	0	6	2850.6516	0.0067
7	2	6	6	2	5	2888.7426	0.0014
7	5	3	6	5	2	2899.0363	0.0001
7	5	2	6	5	1	2899.0363	0.0001
7	4	3	6	4	2	2900.3888	0.0051
7	3	5	6	3	4	2901.7238	0.0060
7	3	4	6	3	3	2905.1473	-0.0024
7	2	5	6	2	4	2933.8128	0.0060
7	1	6	6	1	5	2947.3976	0.0027
8	1	8	7	1	7	3219.8033	0.0018
8	0	8	7	0	7	3246.3277	0.0002
8	2	7	7	2	6	3298.4312	-0.0013
8	5	4	7	5	3	3313.9491	0.0080
8	5	3	7	5	2	3313.9491	0.0080
8	4	5	7	4	4	3315.7819	0.0000
8	4	4	7	4	3	3316.0091	-0.0028
8	3	6	7	3	5	3317.1780	-0.0035
8	3	5	7	3	4	3323.9353	-0.0036
8	2	6	7	2	5	3361.3513	0.0013
8	1	7	7	1	6	3362.3907	-0.0015
9	1	9	8	1	8	3618.5750	-0.0080
9	0	9	8	0	8	3640.3630	0.0018
9	2	8	8	2	7	3706.9768	-0.0019
9	6	4	8	6	3	3727.6878	-0.0104
9	6	3	8	6	2	3727.6878	-0.0104
9	5	5	8	5	4	3729.1529	0.0025
9	5	4	8	5	3	3729.1529	0.0025
9	4	6	8	4	5	3731.6717	-0.0040
9	4	5	8	4	4	3732.2205	-0.0035
9	3	7	8	3	6	3732.6068	0.0006
9	1	8	8	1	7	3774.4912	0.0026
9	2	7	8	2	6	3789.2233	0.0003
10	0	10	9	0	9	4033.6088	-0.0039
10	2	9	9	2	8	4114.2803	-0.0041
10	7	4	9	7	3	4141.5323	-0.0004
10	7	3	9	7	2	4141.5323	-0.0004
10	6	5	9	6	4	4142.7076	0.0010
10	6	4	9	6	3	4142.7076	0.0010
10	5	6	9	5	5	4144.7092	0.0050
10	5	5	9	5	4	4144.7092	0.0050
10	3	8	9	3	7	4147.7957	0.0002
10	4	7	9	4	6	4147.9855	0.0009
10	4	6	9	4	5	4149.1619	0.0006

Table S29 (cont.). Measured frequencies (ν_{obs}) and residuals ($\nu_{obs} - \nu_{cal}$) of the rotational transitions of isomer **7w-1** of fenchone-$(H_2O)_7$.

J'	K'_{-1}	K'_{+1}	*J"*	K''_{-1}	K''_{+1}	ν_{obs}	$\nu_{obs} - \nu_{cal}$
10	3	7	9	3	6	4167.8454	-0.0092
10	1	9	9	1	8	4183.1671	-0.0007
10	2	8	9	2	7	4216.3265	0.0014
11	1	11	10	1	10	4413.8664	-0.0071
11	0	11	10	0	10	4426.7020	0.0020
11	2	10	10	2	9	4520.2841	0.0027
11	6	6	10	6	5	4557.9938	0.0184
11	6	5	10	6	4	4557.9938	0.0184
11	5	7	10	5	6	4560.6455	0.0016
11	5	6	10	5	5	4560.6455	0.0016
11	4	8	10	4	7	4564.6684	-0.0157
11	1	10	10	1	9	4588.0481	0.0003
11	3	8	10	3	7	4593.6274	-0.0058
11	2	9	10	2	8	4641.7565	-0.0054
12	1	12	11	1	11	4810.6024	0.0005
12	0	12	11	0	11	4819.9693	0.0047
12	2	11	11	2	10	4924.9384	0.0034
12	7	6	11	7	5	4971.4891	-0.0055
12	7	5	11	7	4	4971.4891	-0.0055
12	6	7	11	6	6	4973.5244	-0.0076
12	6	6	11	6	5	4973.5244	-0.0076
12	3	10	11	3	9	4976.5971	0.0000
12	5	8	11	5	7	4976.9227	-0.0002
12	5	7	11	5	6	4977.1219	0.0189
12	4	8	11	4	7	4985.9959	-0.0015
12	2	10	11	2	9	5064.8652	0.0028
13	0	13	12	0	12	5213.5412	0.0009
13	2	12	12	2	11	5328.2390	-0.0086
13	8	6	12	8	5	5385.1405	-0.0108
13	8	5	12	8	4	5385.1405	-0.0108
13	1	12	12	1	11	5386.3767	-0.0023
13	7	7	12	7	6	5386.7998	-0.0068
13	7	6	12	7	5	5386.7998	-0.0068
13	6	8	12	6	7	5389.4052	0.0007
13	6	7	12	6	6	5389.4052	0.0007
13	3	11	12	3	10	5389.7708	-0.0040
13	5	9	12	5	8	5393.6621	-0.0057
13	4	9	12	4	8	5406.4177	-0.0063
13	3	10	12	3	9	5452.5641	-0.0075
14	0	14	13	0	13	5607.4439	0.0038
14	2	13	13	2	12	5730.2436	-0.0163
14	1	13	13	1	12	5780.7999	-0.0019
14	8	7	13	8	6	5800.2886	-0.0054
14	8	6	13	8	5	5800.2886	-0.0054
14	7	8	13	7	7	5802.3615	-0.0043
14	7	7	13	7	6	5802.3615	-0.0043
14	6	9	13	6	8	5805.6380	0.0160
14	6	8	13	6	7	5805.6380	0.0160
14	4	11	13	4	10	5816.3486	-0.0088
14	3	11	13	3	10	5884.4299	-0.0185
15	0	15	14	0	14	6001.6263	0.0044
15	2	14	14	2	13	6131.0465	-0.0022
15	1	14	14	1	13	6173.2377	0.0038
15	3	13	14	3	12	6212.7415	0.0156
15	8	8	14	8	7	6215.6504	0.0125

Table S29 (cont.). Measured frequencies (ν_{obs}) and residuals ($\nu_{obs} - \nu_{cal}$) of the rotational transitions of isomer **7w-1** of fenchone-$(H_2O)_7$.

J'	*K'*$_{-1}$	*K'*$_{+1}$	*J''*	*K''*$_{-1}$	*K''*$_{+1}$	ν_{obs}	$\nu_{obs} - \nu_{cal}$
15	8	7	14	8	6	6215.6504	0.0125
15	7	9	14	7	8	6218.1846	-0.0078
15	7	8	14	7	7	6218.1846	-0.0078
15	6	10	14	6	9	6222.2252	0.0102
15	6	9	14	6	8	6222.2252	0.0102
15	2	13	14	2	12	6315.2307	0.0030
15	3	12	14	3	11	6316.5342	0.0204
16	1	16	15	1	15	6393.8430	-0.0017
16	0	16	15	0	15	6396.0306	0.0030
16	2	15	15	2	14	6530.7046	-0.0163
16	1	15	15	1	14	6564.6518	0.0119
16	3	14	15	3	13	6622.2132	0.0090
16	4	12	15	4	11	6679.8165	0.0083
16	2	14	15	2	13	6724.2925	-0.0149

9. Cartesian coordinates of observed species

Table S30. Cartesian coordinates of isomer **4w-1** of fenchone-$(H_2O)_4$ from MP2/6-311++G(d,p) level of theory.

	X	Y	Z
C	-1.477319	-1.190651	-0.297952
C	-2.706170	-0.458508	0.300463
C	-2.334855	0.179188	1.653670
C	-1.322835	1.287548	1.243640
C	-1.345292	1.270525	-0.319682
C	-0.590833	0.003221	-0.660912
C	-2.792130	0.803468	-0.581411
C	-0.847956	2.536708	-0.984624
O	0.539682	-0.071886	-1.129074
C	-1.848320	-1.955627	-1.579800
C	-0.755817	-2.147401	0.656882
O	2.612375	1.700589	-0.075954
O	4.728062	0.003323	-0.661979
O	2.652936	-1.745274	0.047077
O	1.888503	-0.045161	2.065414
H	-1.918058	-0.533547	2.368556
H	-3.229499	0.617976	2.108179
H	-3.600470	-1.090231	0.328518
H	-0.317024	1.110326	1.637233
H	-1.650147	2.279330	1.576462
H	-2.995877	0.607731	-1.638872
H	-3.521875	1.530809	-0.206352
H	-2.449674	-2.832471	-1.314114
H	-0.939540	-2.298641	-2.085174
H	-2.422166	-1.345585	-2.283203
H	0.027997	-2.694782	0.124249
H	-1.470819	-2.877134	1.055148
H	-0.284007	-1.623243	1.491431
H	0.163038	2.787842	-0.649473
H	-1.506792	3.374421	-0.731169
H	-0.829552	2.427014	-2.074291
H	1.994648	-1.458241	-0.598809
H	3.479619	-1.365558	-0.296176
H	1.910444	1.311175	-0.615529
H	2.405277	1.344553	0.803423
H	2.199481	-0.764951	1.476001
H	2.312648	-0.220987	2.909092
H	5.282667	0.257918	-1.401884
H	4.116371	0.752724	-0.528760

Table S31. Cartesian coordinates of isomer **4w-4** of fenchone-$(H_2O)_4$ from MP2/6-311++G(d,p) level of theory.

	X	Y	Z
C	-2.106671	-0.696520	-0.122589
C	-2.117584	-0.086907	1.302572
C	-0.756500	-0.315900	1.989713
C	0.203925	0.576005	1.152217
C	-0.735367	1.333893	0.163229
C	-1.143170	0.261359	-0.830438
C	-2.022985	1.424919	1.007377
C	-0.150934	2.592980	-0.439873
O	-0.757555	0.142451	-1.984357
C	-3.493518	-0.613429	-0.780245
C	-1.592750	-2.136081	-0.228537
O	2.115880	-0.100657	-2.035786
O	3.524872	1.332205	-0.231347
O	3.583746	-0.770514	1.514747
O	2.056176	-2.262533	-0.217218
H	-0.452979	-1.365264	2.009542
H	-0.808355	0.034405	3.026509
H	-2.981204	-0.409456	1.894350
H	0.966780	0.000415	0.622956
H	0.719025	1.320217	1.771109
H	-2.872854	1.833109	0.451246
H	-1.869750	2.022558	1.914106
H	-3.413647	-0.860017	-1.844030
H	-3.942650	0.380007	-0.691481
H	-4.164411	-1.337740	-0.303856
H	-1.703769	-2.485326	-1.260391
H	-2.180742	-2.792531	0.424447
H	-0.535605	-2.222539	0.035934
H	0.778256	2.371990	-0.973265
H	0.076834	3.318610	0.348563
H	-0.851648	3.050473	-1.146456
H	2.610198	-1.864079	0.482937
H	2.402526	-3.152050	-0.322961
H	3.312814	-0.467483	2.384271
H	3.663860	0.047986	0.983624
H	3.021440	0.915409	-0.965354
H	4.280843	1.744327	-0.655817
H	2.057998	-0.962342	-1.588931
H	1.195413	0.109780	-2.245915

Table S32. Cartesian coordinates of isomer **4w-7** of fenchone-$(H_2O)_4$ from MP2/6-311++G(d,p) level of theory.

	X	Y	Z
C	1.677194	1.019402	-0.071725
C	2.463865	-0.009423	0.779663
C	1.567589	-0.557334	1.907311
C	0.500630	-1.376823	1.128091
C	0.983065	-1.328551	-0.356298
C	0.685994	0.093955	-0.784041
C	2.509834	-1.235985	-0.156010
C	0.411210	-2.410453	-1.247019
O	-0.195062	0.461603	-1.549982
C	2.583626	1.695145	-1.113757
C	0.937952	2.103255	0.720120
O	-2.946353	-0.531392	-1.615102
O	-3.071268	-1.463917	0.921815
O	-2.268700	0.980253	1.891040
O	-2.585261	2.121720	-0.706941
H	1.129355	0.225322	2.530620
H	2.158311	-1.208619	2.560487
H	3.432188	0.375474	1.117470
H	-0.513497	-0.985640	1.239502
H	0.483586	-2.426417	1.443914
H	3.058540	-1.075687	-1.089537
H	2.904647	-2.129895	0.341480
H	1.974215	2.259310	-1.827459
H	3.189041	0.976569	-1.673635
H	3.259826	2.392467	-0.605899
H	0.522891	2.846596	0.030693
H	1.638371	2.618682	1.388212
H	0.113801	1.698863	1.313636
H	-0.683248	-2.393249	-1.222260
H	0.739549	-3.396476	-0.900818
H	0.733441	-2.279888	-2.285648
H	-2.470957	1.901981	0.232145
H	-1.683738	2.107945	-1.046323
H	-3.089117	-1.239240	-0.035429
H	-3.838006	-2.024509	1.060005
H	-2.025784	-0.520998	-1.908636
H	-3.090650	0.415980	-1.440206
H	-2.698438	1.263030	2.702008
H	-2.636927	0.093143	1.711016

Table S33. Cartesian coordinates of isomer **4w-16** of fenchone-$(H_2O)_4$ from MP2/6-311++G(d,p) level of theory.

	X	Y	Z
C	1.367247	-1.216857	0.034080
C	2.627990	-0.504499	0.589437
C	3.431777	0.138363	-0.556007
C	2.474728	1.258993	-1.052207
C	1.296747	1.246909	-0.026302
C	0.526326	-0.007033	-0.390898
C	2.021778	0.754761	1.242559
C	0.488602	2.524416	0.030882
O	-0.553736	-0.057027	-0.965546
C	0.656009	-2.002841	1.149085
C	1.598953	-2.151565	-1.157118
O	-2.758026	-1.713755	0.015983
O	-4.719916	-0.088722	-1.055738
O	-2.791222	1.721405	-0.127766
O	-1.931159	0.214241	1.987435
H	3.712237	-0.567902	-1.340792
H	4.356137	0.569360	-0.156981
H	3.219262	-1.151148	1.246439
H	2.119039	1.099746	-2.076637
H	2.950796	2.245519	-1.017332
H	1.333552	0.552060	2.068264
H	2.786695	1.467385	1.574028
H	1.308995	-2.822059	1.472460
H	-0.277573	-2.435155	0.778297
H	0.416305	-1.379528	2.013478
H	0.654961	-2.628634	-1.439454
H	2.314317	-2.936733	-0.885471
H	1.979716	-1.622344	-2.035419
H	0.031731	2.739781	-0.941170
H	1.137071	3.366961	0.295180
H	-0.311302	2.453264	0.773016
H	-2.529285	-1.272260	0.849784
H	-2.020847	-1.449389	-0.550296
H	-2.107679	1.416921	-0.738002
H	-3.598882	1.307498	-0.476872
H	-5.631686	-0.353736	-0.921551
H	-4.180935	-0.813055	-0.683849
H	-2.291429	0.493009	2.832998
H	-2.279671	0.867421	1.343869

Table S34. Cartesian coordinates of isomer **5w-3** of fenchone-$(H_2O)_5$ from MP2/6-311++G(d,p) level of theory.

	X	Y	Z
C	3.186296	-0.284486	-0.559977
C	2.783155	0.741318	0.519720
C	2.506712	-0.203787	1.704846
C	1.782429	-1.400860	1.025222
C	1.868643	-1.086575	-0.503366
C	0.876717	0.038903	-0.693475
C	1.447870	1.297530	-0.040603
C	0.477848	1.857680	1.007364
C	1.691809	2.370054	-1.115652
O	-0.205724	-0.035039	-1.265177
C	1.681236	-2.279134	-1.416732
O	-2.176213	-2.010994	-0.540526
O	-4.338976	-0.386956	-0.908917
O	-1.578239	-1.109169	2.062375
O	-3.305580	0.901511	1.489355
O	-2.424771	1.826110	-1.053402
H	3.383663	0.147835	-1.546179
H	4.044004	-0.898270	-0.260328
H	3.512014	1.530802	0.731461
H	1.912947	0.257934	2.496760
H	3.456573	-0.523156	2.146854
H	0.746288	-1.511980	1.358934
H	2.304904	-2.347390	1.206243
H	0.086787	1.078984	1.667678
H	-0.371840	2.338688	0.513599
H	0.990177	2.611764	1.616559
H	2.420107	2.052463	-1.867621
H	0.753212	2.612202	-1.624862
H	2.067794	3.280197	-0.634821
H	0.715638	-2.762332	-1.234002
H	1.715962	-1.978605	-2.469230
H	2.470396	-3.017457	-1.238355
H	-2.905654	1.465053	0.804873
H	-4.005543	0.475061	0.975766
H	-3.643294	-1.076015	-0.838023
H	-5.074180	-0.818312	-1.350113
H	-1.949374	-1.903023	0.399515
H	-1.451767	-1.550942	-0.985989
H	-3.157037	1.266910	-1.347878
H	-1.647756	1.286549	-1.259216
H	-1.765818	-1.461589	2.936091
H	-2.188477	-0.342937	1.968553

Table S35. Cartesian coordinates of isomer **5w-1** of fenchone-$(H_2O)_5$ from MP2/6-311++G(d,p) level of theory.

	X	Y	Z
C	-2.907921	0.854826	-0.693112
C	-2.901745	-0.276360	0.355160
C	-2.407013	0.497287	1.593393
C	-1.327515	1.444465	0.996665
C	-1.413044	1.206028	-0.545944
C	-0.794531	-0.164075	-0.717391
C	-1.776863	-1.203025	-0.172022
C	-1.117334	-2.098681	0.882072
C	-2.272946	-2.084918	-1.331086
O	0.308758	-0.416168	-1.189738
C	-0.822882	2.309890	-1.397411
O	2.623903	1.367354	-1.177972
O	2.167830	1.960648	1.404541
O	1.670651	-0.538135	2.395951
O	4.492649	-0.686721	-1.038964
O	2.393644	-2.024210	0.098302
H	-3.179465	0.537736	-1.704821
H	-3.546061	1.697100	-0.400242
H	-3.849561	-0.805842	0.500514
H	-2.014758	-0.151925	2.379280
H	-3.235246	1.071988	2.021641
H	-0.320601	1.261938	1.381414
H	-1.564033	2.498299	1.184575
H	-0.531158	-1.529890	1.607980
H	-0.441888	-2.812371	0.399479
H	-1.889171	-2.668514	1.413367
H	-2.948501	-2.851532	-0.934578
H	-2.809690	-1.516234	-2.095690
H	-1.423394	-2.584596	-1.808234
H	-1.384125	3.238402	-1.245787
H	0.221175	2.497453	-1.129971
H	-0.861214	2.050671	-2.461029
H	3.843651	-1.284846	-0.615085
H	4.965841	-1.236868	-1.666271
H	2.397546	1.811984	0.457324
H	2.767119	2.651378	1.696366
H	1.857448	0.827603	-1.417734
H	3.373061	0.752432	-1.278062
H	2.032559	-0.666698	3.276125
H	1.909843	0.382116	2.162563
H	2.184075	-1.624366	0.960338
H	1.637841	-1.760182	-0.442372

Table S36. Cartesian coordinates of isomer **5w-7** of fenchone-$(H_2O)_5$ from MP2/6-311++G(d,p) level of theory.

	X	Y	Z
C	2.203584	0.746750	1.242683
C	2.660818	-0.655575	0.790923
C	3.665187	-0.276806	-0.313819
C	2.955248	0.911538	-1.022796
C	1.693832	1.191116	-0.142762
C	0.782119	0.029576	-0.477634
C	1.364016	-1.231772	0.165384
C	1.558366	-2.332853	-0.882231
C	0.416370	-1.754078	1.260279
O	-0.216610	0.070447	-1.187416
C	1.091158	2.569119	-0.303815
O	-2.414389	1.911309	-0.773927
O	-4.438031	0.103650	-0.936953
O	-1.501946	1.284896	1.832406
O	-3.193759	-0.828467	1.542790
O	-2.335425	-1.905260	-0.955487
H	1.413521	0.748461	1.998413
H	3.037979	1.370744	1.585223
H	3.072888	-1.303586	1.571584
H	3.900430	-1.102432	-0.989285
H	4.604820	0.052146	0.142429
H	2.683204	0.693942	-2.061943
H	3.577035	1.813968	-1.025546
H	2.098226	-1.977860	-1.765003
H	0.584178	-2.706986	-1.212679
H	2.116670	-3.169372	-0.445848
H	0.193578	-0.998083	2.016736
H	-0.525698	-2.086090	0.816342
H	0.885479	-2.614805	1.750898
H	0.746403	2.724963	-1.331882
H	0.238609	2.696521	0.368619
H	1.838845	3.336209	-0.074219
H	-1.607028	-1.322876	-1.216060
H	-3.118592	-1.428966	-1.266144
H	-1.638474	1.697243	2.689511
H	-2.089816	0.496673	1.844855
H	-2.804451	-1.411011	0.868022
H	-3.967287	-0.500973	1.064491
H	-1.639425	1.521479	-1.200565
H	-2.138489	1.919258	0.158289
H	-5.208647	0.429534	-1.407217
H	-3.796855	0.847210	-0.947608

Table S37. Cartesian coordinates of isomer **6w-1** of fenchone-$(H_2O)_6$ from MP2/6-311++G(d,p) level of theory.

	X	Y	Z
C	3.421517	0.149292	-0.511317
C	2.801083	1.096358	0.536199
C	1.389685	1.357464	-0.053022
C	1.101242	-0.000744	-0.690346
C	2.291939	-0.901348	-0.449232
C	2.239705	-1.188491	1.085990
C	2.702503	0.143164	1.742341
C	0.307434	1.715377	0.972904
C	1.426123	2.443910	-1.140306
C	2.367853	-2.128769	-1.331979
O	0.074776	-0.302743	-1.290206
O	-1.434968	-2.596691	-0.439628
O	-3.787850	-1.375800	-0.930970
O	-1.165025	-1.469253	2.102057
O	-3.363849	0.004611	1.590014
O	-3.105586	2.532663	0.371892
O	-2.460798	1.018252	-1.794158
H	2.018153	0.495428	2.517080
H	3.691304	0.028944	2.199511
H	3.350959	2.022262	0.735651
H	3.541969	0.589951	-1.506473
H	4.381059	-0.270893	-0.187215
H	2.361261	-1.852754	-2.391740
H	3.286930	-2.686518	-1.122162
H	1.517586	-2.794545	-1.149915
H	0.072058	0.878431	1.635548
H	-0.616448	2.012370	0.470148
H	0.645630	2.562779	1.581468
H	2.222049	2.278656	-1.872772
H	0.469362	2.476985	-1.671893
H	1.589521	3.418542	-0.666729
H	1.238594	-1.490551	1.408924
H	2.931700	-2.010913	1.301617
H	-0.808922	-2.027926	-0.908448
H	-1.268747	-2.375927	0.495212
H	-4.459178	-1.944769	-1.315118
H	-2.999128	-1.951569	-0.801521
H	-1.290890	-1.865613	2.968069
H	-1.936899	-0.865652	1.998280
H	-3.325398	0.943558	1.339197
H	-3.747668	-0.420416	0.809069
H	-3.011134	0.226582	-1.685675
H	-1.561793	0.668923	-1.703503
H	-3.742022	3.224239	0.178381
H	-2.898015	2.124801	-0.494389

Table S38. Cartesian coordinates of isomer **6w-2** of fenchone-$(H_2O)_6$ from MP2/6-311++G(d,p) level of theory.

	X	Y	Z
C	-2.522420	-0.567568	1.239977
C	-2.698390	0.915709	0.853524
C	-1.324710	1.248126	0.215616
C	-1.018705	-0.072329	-0.490825
C	-2.136656	-1.044235	-0.174663
C	-3.332153	-0.480573	-1.010508
C	-3.778391	0.792658	-0.237560
C	-1.313397	2.416687	-0.774508
C	-0.267098	1.509085	1.303245
C	-1.830815	-2.506856	-0.412501
O	-0.057876	-0.287951	-1.221918
O	1.578568	-2.549136	-0.429655
O	1.144179	-1.569339	2.075274
O	3.913447	-1.099010	-1.018175
O	2.471039	0.965343	-1.918369
O	2.960589	2.544689	0.311156
O	3.376773	0.166364	1.554376
H	-3.855755	1.677001	-0.874202
H	-4.756698	0.637304	0.229512
H	-2.956517	1.598980	1.669313
H	-1.735654	-0.755922	1.975753
H	-3.458470	-1.027770	1.579157
H	-1.542807	-2.677264	-1.455821
H	-1.012167	-2.850469	0.226157
H	-2.716312	-3.115368	-0.198901
H	-1.680941	3.325160	-0.283144
H	-1.930428	2.224693	-1.657375
H	-0.289870	2.604998	-1.113303
H	-0.174970	0.679770	2.007839
H	0.710980	1.680486	0.848035
H	-0.546325	2.415654	1.853154
H	-3.040178	-0.276570	-2.047048
H	-4.121121	-1.240941	-1.032596
H	1.547114	0.719638	-1.763681
H	2.636039	1.680177	-1.277962
H	4.688418	-1.279955	-1.555380
H	3.465938	-0.331711	-1.451170
H	3.615448	3.200294	0.560071
H	3.143157	1.767550	0.881647
H	2.618040	-0.348487	1.875371
H	3.734555	-0.378285	0.838272
H	2.462685	-2.250133	-0.701303
H	0.989322	-2.010367	-0.974551
H	1.184848	-2.269240	2.732118
H	1.264317	-2.031141	1.217737

Table S39. Cartesian coordinates of isomer **7w-1** of fenchone-$(H_2O)_7$ from MP2/6-311++G(d,p) level of theory.

	X	Y	Z
C	1.246411	-0.010722	-0.447605
C	1.946605	1.254392	-0.008980
C	2.573407	0.769016	1.314082
C	3.262588	-0.469928	0.705077
C	2.070545	-1.204578	0.037953
C	4.144762	0.197678	-0.366219
C	3.206231	1.298966	-0.935418
C	1.106578	2.512442	-0.015734
C	2.422325	-2.117877	-1.141852
C	1.286191	-2.022000	1.077972
O	0.240171	-0.085473	-1.145536
O	-1.765315	-2.096995	-1.041405
O	-2.521806	-1.899798	1.542505
O	-4.425515	0.020989	0.683320
O	-3.846327	-0.350104	-1.903230
O	-2.702610	2.260470	1.000717
O	-1.926697	1.738827	-1.520958
O	-0.735680	0.366995	2.058649
H	0.735518	2.731414	-1.023127
H	0.249363	2.403730	0.653116
H	1.707938	3.365402	0.317821
H	3.294112	1.496256	1.707547
H	1.816294	0.550984	2.071140
H	3.810117	-1.110687	1.404394
H	4.506352	-0.496209	-1.128799
H	5.021941	0.647479	0.110761
H	2.941667	1.140007	-1.987261
H	3.652628	2.296544	-0.854380
H	1.521350	-2.629549	-1.495746
H	3.146859	-2.876639	-0.823817
H	2.844826	-1.565210	-1.986116
H	1.901052	-2.875985	1.386163
H	0.360988	-2.407553	0.639100
H	1.029158	-1.431912	1.959179
H	-1.000997	-1.515518	-1.180488
H	-2.474943	-1.657031	-1.536797
H	-4.001614	0.850202	0.956765
H	-3.930014	-0.661505	1.163481
H	-2.224977	-2.089678	0.622789
H	-2.600150	-2.756975	1.968326
H	-1.367603	1.080384	1.893145
H	-1.295296	-0.422258	2.043440
H	-2.408959	2.190806	0.062162
H	-2.869131	3.194669	1.147883
H	-2.569692	1.110037	-1.884825
H	-1.105950	1.223752	-1.471168
H	-4.624799	-0.447886	-2.456984
H	-4.189667	-0.220705	-0.985852
